# Towards New Delivery Agents for Boron Neutron Capture Therapy: Synthesis and In Vitro Evaluation of a Set of Fluorinated Carbohydrate Derivatives

**DOI:** 10.3390/molecules29174263

**Published:** 2024-09-09

**Authors:** Jelena Matović, Juulia Järvinen, Iris K. Sokka, Surachet Imlimthan, Olli Aitio, Mirkka Sarparanta, Jarkko Rautio, Filip S. Ekholm

**Affiliations:** 1 Department of Chemistry, University of Helsinki, FI-00014 Helsinki, Finland; jelena.matovic@helsinki.fi (J.M.); iris.sokka@helsinki.fi (I.K.S.); surachet.ilmimthan@helsinki.fi (S.I.); mirkka.sarparanta@helsinki.fi (M.S.); 2 School of Pharmacy, University of Eastern Finland, FI-70211 Kuopio, Finland; juulia.jarvinen@uef.fi (J.J.); jarkko.rautio@uef.fi (J.R.); 3 Glykos Finland Ltd., FI-00790 Helsinki, Finland; olli.aitio@glykos.fi

**Keywords:** boron neutron capture therapy, cancer therapeutics, carbohydrates, chemical synthesis, structural elucidation, preclinical profiling

## Abstract

Boron Neutron Capture Therapy (BNCT) is a cancer treatment which combines tumor-selective boron delivery agents with thermal neutrons in order to selectively eradicate cancer cells. In this work, we focus on the early-stage development of carbohydrate delivery agents for BNCT. In more detail, we expand upon our previous GLUT-targeting approach by synthesizing and evaluating the potential embedded in a representative set of fluorinated carbohydrates bearing a boron cluster. Our findings indicate that these species may have advantages over the boron delivery agents in current clinical use, e.g., significantly improved boron delivery capacity at the cellular level. Simultaneously, the carbohydrate delivery agents were found to bind strongly to plasma proteins, which may be a concern requiring further action before progression to in vivo studies. Altogether, this work brings new insights into factors which need to be accounted for if attempting to develop theranostic agents for BNCT based on carbohydrates in the future.

## 1. Introduction

Boron Neutron Capture Therapy (BCNT) is a non-invasive cancer therapy on the rise globally. The functional principle of BNCT is based on preferential delivery of ^10^B atoms to cancer cells, followed by their irradiation with low-energy thermal neutrons. The neutron irradiation causes ^10^B (through an excited ^11^B* state) to undergo a fission reaction with one of the fission products, ^4^He nuclei. These ^4^He nuclei, also called high linear energy transfer (LET) alpha particles, have a destructive range of 5–9 μm, which is approximately the diameter of a single cell [1,2,3]. In theory, this may restrict the destructive effect on cancerous tissue, but in reality this only occurs if the tumor-selectivity of the ^10^B-delivery agents is sufficiently high. This is where the three boron delivery agents in current clinical use, i.e., sodium-borocaptate (BSH) [4], boronophenylalanine (BPA) [5] and decahydrododecaborate (GB-10) [6], display significant limitations, thus highlighting the need for development of alternative boron delivery strategies.

In recent work [7,8,9,10], we have explored the potential of targeting glucose transporters (GLUTs) which are overexpressed in head and neck cancers (among others) [11]. Through the synthesis and in vitro assessment of libraries of carbohydrate-based delivery agents [7,8,9,10], we have found that they have beneficial properties compared to the agents in current clinical use. Most noteworthy of these properties is their significantly improved boron delivery capacity at the cellular level. Nevertheless, the previous synthetic glycoconjugate libraries did not address one of the most important aspects of personalized clinical BNCT-treatments. This is the ability to assess in real-time when the optimal time point for the neutron irradiation is. In clinical BNCT-treatments with the BPA-fructose formulation [12,13,14,15], fluorine-18-labeled 4-borono-2-[^18^F]fluoro-L-phenylalanine (F-BPA) has been employed in imaging of BPA uptake/biodistribution via positron emission tomography (PET) [15,16]. This has provided an excellent base for patient treatment planning and contributed to positive treatment outcomes. From a carbohydrate point-of-view, 2-deoxy-2-[^18^F] fluoro-D-glucose ([^18^F]FDG) has been the gold standard employed in PET imaging of head and neck cancers for decades due to its ability to accumulate in cancer cells [13,17,18].

**Figure 1:**
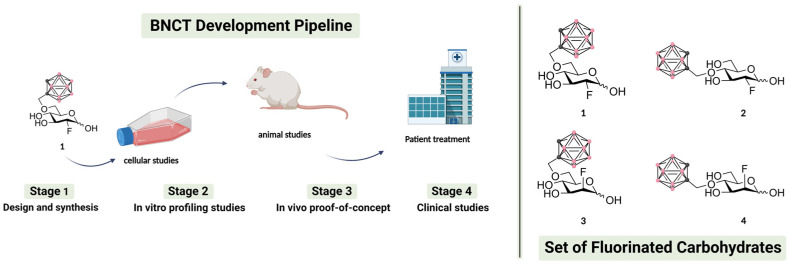
**Left panel**: a schematic representation of the four main stages of a BNCT development pipeline (figure created with Biorender.com). In this work we focus on stages 1–2. **Right panel**: chemical structures of the fluorinated carbohydrates targeted in this work. Substrates **1**–**2** are based on the FDG backbone whereas **3**–**4** are based on the FDM backbone. In the *ortho*-carboranylmethyl moiety, black/grey dots represent carbon atoms while pink dots represent boron atoms.

These factors combined with the promising findings from our earlier work on the development of GLUT-targeting agents for BNCT, provoked our interest in exploring the potential of delivery agents based on the FDG structural backbone. Instead of directly targeting radiolabeled theranostic agents, we decided to investigate the fundamental properties of FDG and 2-deoxy-2-fluoro-D-mannose (FDM) derivatives (see Figure 1) containing fluorine-19 in position 2 in order to understand whether the development of theranostic agents based on this backbone would in the future be plausible or not. In line with these thoughts, this work deals with the design, synthesis and early-stage in vitro profiling of a representative set of FDG and FDM derivatives bearing an *ortho*-carboranylmethyl substituent in either position 4 or 6. The early-stage in vitro profiling data highlight concerns which will require further considerations and action before attempting to develop a theranostic approach to BNCT based on FDM and FDG derivatives.

## 2. Results and Discussion

Carbohydrate-based delivery agents for BNCT have been developed and explored by a number of research teams over the past decades [19,20]. Nevertheless, the selection of target molecules for the current study was based on our own dedicated synthesis and screening program in which libraries of systematically modified glycoconjugates have been produced and assessed under identical experimental conditions [7,8,9,10]. Based on these studies, the preferential position for attachment of an *ortho*-carboranylmethyl substituent in D-glucose is the 6th position [7]. Thus, the main objective was to synthesize the corresponding conjugate based on the FDG structural skeleton. However, while reviewing the literature and designing the synthetic routes, we noted that both “manno” and “gluco”-isomers could be accessed with the *ortho*-carboranylmethyl substituent in positions 4 or 6 through a more carefully devised scheme. Since small structural variations in the glycoconjugate delivery agents have previously been accompanied by differences in in vitro performance, the simultaneous synthesis and screening of a representative set of four fluorinated glycoconjugates was thought to be a considerable advantage over the synthesis of only one.

### 2.1. Chemical Synthesis and Structural Characterization

**Scheme 1:**
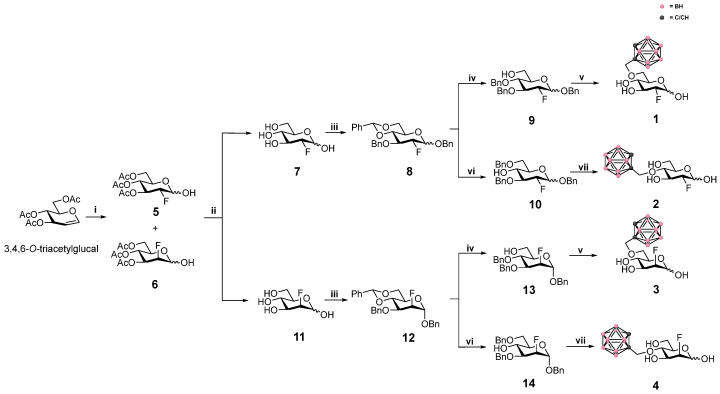
The synthetic routes and reaction conditions leading to end products **1**–**4**: (i) SelectFluor, acetone:H_2_O 5:1, RT, 21 h, 67%; (ii) (1) Ac_2_O, DMAP, pyridine, RT, 2 h, **FDG** 41%, **FDM** 54%, (2) NaOMe, MeOH, RT, 1.5 h, **7** 99%, **11** 99%; (iii) (1) C_6_H_5_CH(OCH_3_)_2_, *p*-TsOH, DMF, 60 °C, 0.2 bar, 2–3 h, **FDG** 82%, **FDM** 94%, (2) NaH, BnBr, DMF, RT, 1.5–3 h, **8** 72%, **12** 66%; (iv) borane/THF, Cu(OTf)_2_, DCM, RT, 1.5–3 h, **9** 56%, **13** 53% (v) (1) NaH, propargyl Br, DMF, RT, 1.5–3 h, **FDG** 85%, **FDM** 81%, (2) B_10_H_14_, ACN, toluene, 60 → 80 °C, **FDG** 49%, **FDM** 42%, (3) H_2_, 10% Pd/C, EtOAc:MeOH 7:1, 4 bar, 4 h, **1** 85%, **3** 72%; (vi) Et_3_SiH, TFA, DCM, RT, 2 h, **10** 89%, **14** 83%; (vii) (1) NaH, propargyl Br, DMF, RT, 1.5–3 h, **FDG** 83%, **FDM** 86%, (2) B_10_H_14_, ACN, toluene, 60 → 80 °C, **FDG** 53%, **FDM** 54%, (3) H_2_, 10% Pd/C, EtOAc:MeOH 7:1, 4 bar, 4 h, **2** 90%, **4** 71%. Please note that **5** and **6** were obtained as a mixture of epimers after step (i), and that, in the substrate-specific analytical data given in the experimental section, the data is instead given after two steps when the fully acetylated epimers have been isolated (i.e., after step (i) and step (ii) (1) in the scheme).

**Chemical synthesis:** The synthetic route leading to the four glycoconjugates (**1**–**4**) is summarized in Scheme 1. We started the synthesis from commercially available 3,4,6-tri-*O*-acetyl-D-glucal. The first step involved the site-selective fluorination of the alkene using SelectFluor. The protocol by Lebedel et al. [21] was utilized, which gave access to both C-2 epimers of the product. These will be referred to as FDG (**5**) and FDM (**6**) derivatives herein to emphasize the “gluco” or “manno” stereochemistry. While the two epimers could not be easily separated by column chromatography at this stage, acetylation of the anomeric hydroxyl groups enabled purification and isolation of the epimers. Via the synthetic protocol employed, the dominating epimer formed in the fluorination reaction was the FDM analogue (3:2 ratio observed between FDM and FDG). The overall yield for the two-step protocol was roughly 63%. This yield encompasses the combined isolated amounts of both fully acetylated FDM and FDG species. From this stage forward, the reactions were run in parallel for both species as showcased in Scheme 1.

In accordance with our previous synthetic strategies employed in the synthesis of *ortho*-carboranylmethyl bearing glycoconjugates [7,8,9,10], we sought to exchange the acetyl protective groups with protective groups, which can be removed through hydrogenolysis in a single step at the end of the synthetic pathway. We were especially interested in a protective group strategy that would offer us the possibility to generate a representative set of structural analogues simultaneously. While reviewing protective groups for this purpose, we decided to take advantage of the possibilities provided by installing a benzylidene acetal at positions 4 and 6 while protecting the other hydroxyl groups as benzyl ethers. The reason why we considered the benzylidene acetal especially appealing is that, without a hydroxyl group at position 2 in FDG and FDM, there would be no selectivity issues regarding the sites at which the benzylidene acetal is installed in the reaction. In addition, the benzylidene acetal can be selectively ring-opened in distinct ways, thus giving an excellent base for diversifying the substrate library.

We proceeded on the synthetic route by removing the acetyl protective groups under Zemplén conditions (NaOMe in MeOH) [22] in excellent yields. With access to the deprotected species **7** (FDG) and **11** (FDM), we successfully formed the benzylidene acetal between the 4-OH and 6-OH groups through the use of a previously reported protocol [23]. In more detail, the deprotected monosaccharide and benzylidene dimethyl acetal were dissolved in DMF and a catalytic amount of *p*-TsOH was added. The acid catalyzed reaction was performed on the rotavapor using a water bath temperature of 60 °C and reduced pressure of 200 mbar. The reaction resulted in 82–94% isolated yields for the FDG and FDM derivatives, respectively. The remaining hydroxyl groups were alkylated through a conventional Williamson ether synthesis protocol employing NaH for deprotonation of the hydroxy groups followed by the addition of BnBr as a potential electrophilic species for the substitution reaction. Acceptable isolated yields in the 66–72% range were observed for substrates **8** and **12**. For selective ring-opening of the benzylidene acetal to yield the 4-OBn/6-OH species **9** and **13**, we employed a similar protocol to that originally reported by Shie et al. [24]. In this protocol, the selection of Lewis acid is thought to be crucial. In more detail, the reaction outcome is determined by whether borane is activated during the reaction or whether the Lewis acid is the most electrophilic species, since the most electrophilic species will coordinate with the 6-OH group of the sugar, thus yielding the sought after stereospecificity. In accordance with the reported protocol, we performed the ring-opening with 1M borane in THF employing a catalytic amount of Cu(OTf)_2_ as the Lewis acid. The isolated yields of **9** and **13** were moderate, yet acceptable, and we did not attempt to optimize the reaction conditions further. In contrast, the selective ring-opening to yield the 4-OH/6-OBn species **10** and **14** employing Et_3_SiH and a catalytic amount of TFA [25] could be achieved in excellent yields in the 83–89% range. The free hydroxyl groups in substrates **9**, **10, 13**, and **14** were next alkylated with propargyl bromide to install a handle for attachment of the boron cluster decaborane (B_10_H_14_). The alkylation proceeded smoothly with yields of 81–86%. The synthesis of the protected glycoconjugates bearing the *ortho*-carboranylmethyl substituent was performed in the following way. First, decaborane was refluxed in ACN to yield the B_10_H_12_·2ACN complex. Next, the carbohydrate species bearing the alkyne handle were dissolved in toluene and added to the reaction mixture. After heating overnight at 80 °C, quenching of the reaction with MeOH, and purification of the reaction mixtures, the *ortho*-carboranylmethyl bearing glycoconjugates could be isolated in yields of 42–54%. These moderate yields are typical for this specific reaction [7,26]. Lastly, the benzyl protective groups were removed via our standard hydrogenolysis protocol employing a catalytic amount of Pd/C and a H_2_-pressure of 4 bar in a solvent mixture containing EtOAc and MeOH [7]. The yields in the final deprotection were 71–90%. This gave access to the four fluorinated FDG and FDM analogues (**1**, **2**, **3**, and **4**) bearing an *ortho*-carboranylmethyl substituent in either position 4 or 6.

**Structural characterization:** During the synthesis work, all intermediates, as well as end products, were carefully characterized by a combination of 1D (^1^H, TOCSY, ^13^C{H} and ^11^B{H}) and 2D (COSY, ed-HSQC, HMBC) NMR spectroscopic techniques, as well as with high-resolution mass spectrometry (HRMS). For the end products submitted for the early-stage biological profiling program, the purity was further assessed by qNMR-techniques and only samples with purity > 95% were evaluated (protocols described earlier [10]).

The structural characterization by NMR spectroscopy was especially challenging due to the presence of anomeric mixtures and the effects of ^19^F on the coupling patterns. Nevertheless, our typical NMR characterization workflow utilizing the NMR spectroscopic techniques mentioned above and quantum mechanical spectral analysis (QMSA) with the Chemadder software was found to be adequate for solving the chemical shifts and coupling constants [27]. While we have in earlier work discussed in detail the characterization workflow employed in our team [7,8,9,10], and information on the use of the QMSA is present in the literature [28,29,30], we will here give a short general overview of how the intermediates and end products were characterized before commenting on the difficulties added by ^19^F.

**Figure 2:**
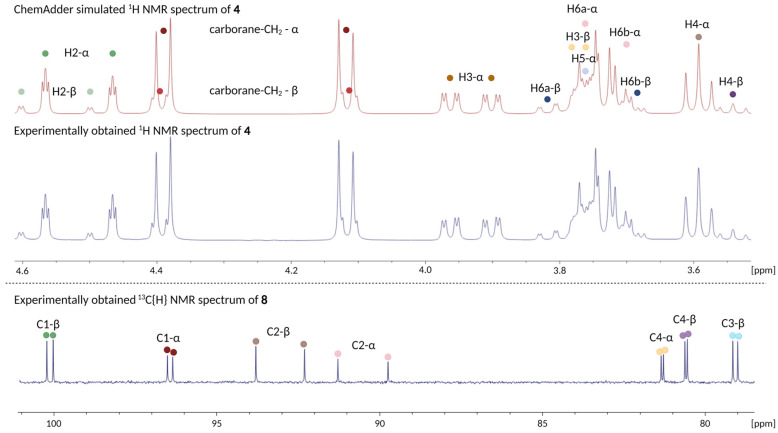
**Top**: Comparison of experimentally obtained ^1^H NMR spectrum of glycoconjugate **4** (blue) and the corresponding simulated spectrum using ChemAdder (red) (range from 4.6–3.5 ppm). **Bottom**: ^13^C{H} NMR spectrum of glycoconjugate **8** (range from 101–78 ppm), showcasing the splitting of carbon signals due to the presence of the fluorine atom.

In short, the anomeric protons and carbons and those of the 6th position in carbohydrates are easily identified in the ^1^H and ^13^C NMR spectra, respectively, through the use of Ed-HSQC. The H-1–H-2 coupling constants can be utilized to distinguish which anomer is α and β in the FDG backbone but for FDM-derivatives the situation is different. Here, in addition to analyzing the H-1–H-2 coupling constant, the location of the H-3/H-5 protons need to be considered [31,32]. We employ 1D-TOCSY on well separated signals in each anomer in order to isolate the individual ^1^H spin systems of the anomers present in the mixture. Once the locations of protons in each spin system are known, COSY can be utilized to identify the chemical shifts of neighboring protons in the backbone i.e., H-2 and H-5 from the starting point presented above. These can in turn be utilized to identify the remaining protons in the carbohydrate backbone i.e., H-3 and H-4. Ed-HSQC can then be utilized to identify the corresponding carbon atoms and, once these signals are resolved, HMBC can be utilized to identify the location of substituents, such as protective groups or the carboranylmethyl substituent. While a rough estimation, and at times complete characterization, of ^1^H and ^13^C NMR spectra is possible based on these techniques alone, in complex ^1^H NMR spectra with overlapping signals and higher order effects these techniques are not always sufficient if the aim is to obtain accurate chemical shifts and coupling constants. In such cases, the use of QMSA provides a good outlet for going beyond what is possible otherwise. As mentioned above, we have used the ChemAdder program for these purposes. A testament to the accuracy of this program is given in Figure 2 by comparing the experimental and simulated ^1^H NMR spectra of **4** (see Figure 2, top).

With the short general overview provided, we will next focus on the effects of fluorine. The replacement of the 2-OH group in D-Glc and D-Man with a fluorine atom significantly increases the spectral complexity, especially when anomeric mixtures of compounds are characterized. For example, the *J*_2,F_ coupling constants (between H-2 and fluorine) can reach absolute values up to 50 Hz, thus splitting the signals over a considerable range in the ^1^H NMR spectrum. The effects of the fluorine atom were not limited to the ^1^H NMR spectrum, as signal splitting could likewise be observed in the ^13^C{H} NMR spectra for multiple signals (see example in Figure 2, bottom). As mentioned above, despite the fact that the ^1^H and ^13^C NMR spectra recorded herein were challenging to solve, they could nevertheless be solved at a high level through the NMR characterization workflow employed in our team. Altogether, comprehensive structural characterization data were collected on each intermediate on the synthetic pathways as well as for the final glycoconjugates submitted to the early-stage in vitro screening program. The characterization data are supplied in the experimental section below and the ^1^H, ^13^C and ^11^B NMR spectra are available in the Supporting Information.

### 2.2. Early-Stage In Vitro Assessment

There are a set number of parameters which are essential to cover in the early-stage in vitro assessment of BNCT delivery agents [33,34]. These include general features, such as cellular toxicity and boron delivery capacity, and more specific properties, such as the biochemical functional basis of the approach on the whole. More importantly, these properties should be assessed under standardized experimental conditions to allow comparison of results between similar and distinct targeting strategies. In the case of the GLUT targeting strategy, our earlier studies provided an excellent base to build upon. In more detail, for the assessment of cytotoxicity, GLUT affinity and boron delivery capacity, the CAL 27 cell line was chosen. The CAL 27 cell line is a squamous carcinoma cell line of human origin and thus a sound choice for assessment of strategies targeting head and neck cancers. The most promising boron delivery agent identified was further subjected to plasma stability and plasma protein binding studies.

**Figure 3:**
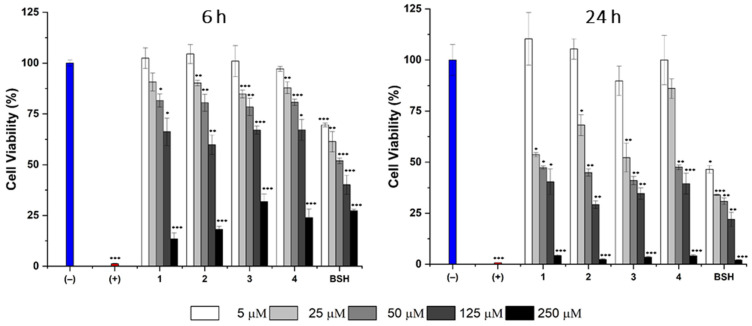
Cell cytotoxicity studies in the CAL 27 cell line measured at two different time points, 6 h (**left**) and 24 h (**right**) over a concentration range of 5–250 μM. The complete cell culture medium containing 1% *v*/*v* DMSO was used as a negative control for cytotoxicity (blue bar) and 1% *v*/*v* Triton X-100 (red bar) as a positive control for cytotoxicity. The cytotoxicity of glycoconjugates **1**–**4** as well as BSH were measured. Results are represented as the mean ± s.d. (n = 4) in comparison with the negative control. The statistical significance was analyzed using an unpaired Student’s *t*-test, where the significance was set at * *p* < 0.05, ** *p* < 0.01, and *** *p* < 0.001.

**In vitro cytotoxicity:** The cellular cytotoxicity was assessed in the CAL 27 cell line at the time points of 6 and 24 h over the concentration range of 5–250 μM. In these studies, the cell medium containing 1% *v*/*v* DMSO was utilized as a negative control for cytotoxicity, Triton X-100 (1% *v*/*v*) as a positive control for cytotoxicity, and BSH as a representative boron delivery agent approved for clinical use. The cell viability was assessed by the commercial CellTiter-Glo luminescent assay. The results of the cellular cytotoxicity studies are summarized in Figure 3.

As seen in Figure 3, the fluorinated carbohydrate delivery agents display a dose-dependent toxicity profile with the concentration of 250 μM being toxic at the 6 h mark (<40% of cells viable). In our earlier work, the corresponding glycoconjugate delivery agents without the fluorine atom were better tolerated (IC_50_-values in the 200–220 μM range) [7,8]. Thus, the introduction of a fluorine atom in the C-2 position of the carbohydrate backbone is accompanied with a slight increase in cellular cytotoxicity. Nevertheless, at the concentration range of 5–125 μM, the synthetic compounds **1**–**4** are less toxic than the clinically employed delivery agent BSH, thus suggesting that, at these concentrations, the harmful effects on the cellular level would be acceptable from the clinical perspective. Altogether, the in vitro cytotoxicity study did not yield results which would undermine the use of fluorinated carbohydrate delivery agents in BNCT. Nevertheless, a more comprehensive in vivo toxicological assessment would be required at later stages of a potential drug development campaign.

**GLUT affinity:** In a strategy targeting the GLUTs, it is important to study whether the monosaccharide delivery agents will be able to compete for the transporters with the high D-glucose levels found in blood. For these purposes, we utilized our previously developed cis-inhibition assay in the CAL 27 cell line [7]. In addition to having accurate protocols for assessment of GLUT-affinity, we have previously studied the GLUT1 expression and verified its function in the CAL 27 cell line [7]. Thus, a strong foundation was available for the current study and we employed an identical protocol herein in the assessment of GLUT affinity, i.e., in assessing to which extent the delivery agents will be able to compete for the GLUTs against the natural substrate D-glucose. In our previous studies, we found that small structural variations in the carbohydrate-boron cluster linker had a considerable effect on the affinity of the compounds [7,8,9,10]. On a general level, the *ortho*-carboranylmethyl substituent significantly enhanced the affinity to the GLUTs (compared to regular D-glucose), whereas the stereochemistry in the carbohydrate core was found to only have minor effects on the GLUT1 affinity (mannose, allose and galactose derivatives studied) [7,8,9,10]. 

As expected, the FDG and FDM analogues bearing an *ortho*-carboranylmethyl substituent did also display a substantially higher GLUT1 affinity than D-glucose (see Table 1). The IC_50_-values were found to be 78.26 μM for **1**, 57.54 μM for **2**, 62.62 μM for **3**, 59.54 μM for **4** and >1 mM for D-glucose. The replacement of the hydroxy group at position 2 with a fluorine atom does have a minor negative effect on the GLUT1 affinities displayed. In more detail, this structural modification is accompanied by a 20–30 μM decrease in the GLUT1 affinity. The difference between the FDG and FDM epimers is negligible when the *ortho*-carboranylmethyl substituent is in position 4 (~2 μM) and slightly larger when this substituent is in position 6 (~16 μM). Conclusions regarding the effect of the anomeric ratio cannot be made based on this series and are not commented on in this work. While subtle differences could be uncovered, the GLUT1 affinities displayed by the FDG and FDM analogues are all within the same range and these agents would be expected to be able to compete for the glucose-transporters against D-glucose.

**Figure 4:**
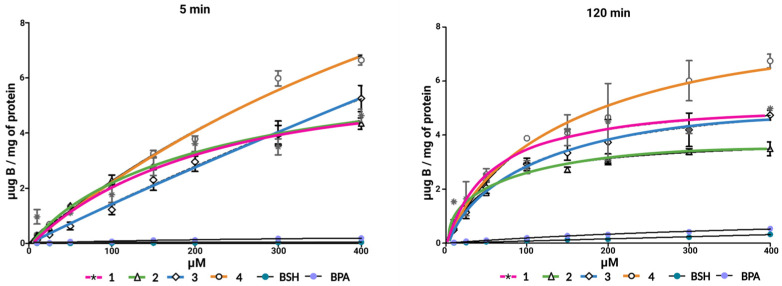
Boron delivery capacity as assessed by ICP-MS after incubation in CAL 27 cells and work-up. The graphs display the results obtained at two separate time points (5 min, 120 min) over the concentration range of 10–400 μM (n = 3 for each time point and concentration). The boron delivery capacity is given on the scale μg B/mg of protein.

**Boron delivery capacity:** A central property of boron delivery agents is their boron delivery capacity. In our previous work we have shown that most of our carbohydrate delivery agents do display superior boron delivery capacity at the cellular level compared to agents in current clinical use (BPA and BSH). Herein, we studied the boron delivery capacity at two time points (5 min, and 120 min) over the concentration range of 10–400 μM (see summary in Figure 4). Our previously employed protocol was utilized in order to be able to compare the results to those obtained earlier [7,8]. In short, after quenching of the incubation, washing, lysis, and digestion with nitric acid, the boron content could be determined by ICP-MS. While we did study the entire concentration range from 10–400 μM, the concentration range of 250 μM and above is not relevant based on the in vitro cytotoxicity assays due to the substantial toxicity displayed by the FDG and FDM delivery agents.

The same trend observed in our earlier work was identified also for the FDG and FDM based delivery agents synthesized herein. Across the concentration range studied, the carbohydrate delivery agents have a far superior boron delivery capacity compared to that of the clinically employed boron delivery agents BPA and BSH. This shows that, at the cellular level, targeting of the GLUT transporters translates into a significantly more efficient boron delivery strategy than the amino acid transporters targeted by BPA [6], or the passive transport mechanism reported for BSH [35]. Moreover, in the fluorinated series of compounds: the FDM analogue bearing the *ortho*-carboranylmethyl substituent in position 4 displays the best boron delivery capacity (although with a very small margin). This is, nevertheless, a surprising finding since our studies on the *ortho*-carboranylmethyl positional isomer library of D-glucose [8], and other studies with similar libraries [36], have indicated that the 2nd or 6th position would be optimal. Thus, an improved and more comprehensive platform would be welcomed for assessing the functional basis of GLUT-targeting agents in the future. In addition, while the results are promising, it is important to note that the excellent in vitro performance uncovered is not a direct testament to a superior in vivo performance.

**Figure 5:**
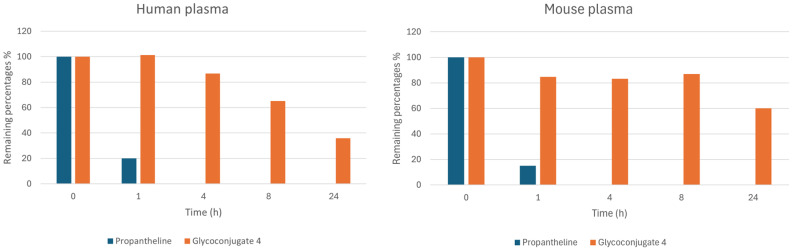
Human and mouse plasma stability of glycoconjugate **4** and propantheline over a 24-h period at a set concentration of 50 μM for **4** and 5 μM for propantheline.

**Plasma stability and plasma protein binding studies:** In the last step of the early-stage in vitro profiling program we decided to focus on the plasma stability and plasma protein binding of the best delivery agent out of the series synthesized, i.e., the FDM derivative **4**. These studies provide important insights into the stability and behavior of the delivery agent in a biological setting, which is a key factor at later stages of the development pipeline [37]. The serum stability studies were performed in both human and mouse plasma at a set concentration of 50 μM over a time period of 24 h. Propantheline (5 μM) was utilized as a control substrate for validating the assay. The results are summarized in Figure 5. In mouse plasma, the stability of **4** was acceptable with 84.7% of the conjugate remaining intact at 1 h and 60% at the 24 h mark. In human plasma, the stability profile was distinct. Here, the glycoconjugate remained stable for 1 h, after which it slowly decomposed and, at the 4 h and 24 h marks, respectively, 86.7% and 39.5% remained intact. While the boron delivery agent **4** does decompose in human plasma over time, the rate at which this occurs is acceptable when taking into account that the recommended guidelines for FDG imaging, thus tumor delivery, is between 55–75 min post administration [38]. Nevertheless, the *ortho*-carboranylmethyl-substituent is likely to affect the pharmacokinetic properties of the delivery agents and thus comprehensive biodistribution studies will be needed in the future to assess these factors in more detail.

Through the plasma protein binding assay, we sought to provide a baseline for future assessment of the ADME-properties of FDM derivative **4**. In more detail, this assay would let us evaluate to which extent the FDM derivative **4** would remain in an unbound state in the presence of plasma proteins. These studies were performed through the standard equilibrium dialysis protocol at the time points of 0 and 6 h employing a concentration of 1 μM. While the studies were performed in both human and mouse plasma, the results were similar in both cases. Being a relatively lipophilic species, it was unsurprising that the FDM derivative **4** bound in significant amounts to the plasma proteins (~97% bound vs. 3% unbound). The binding was, however, surprisingly strong and, even at the 6 h mark, ~80–87% remained in the bound state. The high-level binding observed would significantly limit the amount of free delivery agent in a biological environment and is a factor that needs to be accounted for if these agents were to be advanced to the in vivo screening stage.

## 3. Conclusions

In recent years, we have explored the potential embedded in a GLUT-targeting strategy to BNCT. We have focused on the construction of compound libraries and mapping of the biochemical foundations of the approach on the whole and found that these agents display promising properties in vitro when compared to the boron delivery agents in current clinical use. However, in order for GLUT-targeting agents to become relevant for clinical use, imaging of boron tumor content is crucial. In the clinics, fluorine-18 labeled FDG is one of the golden standards employed in tumor imaging (PET) in head and neck cancers. Thus, we became interested in exploring the suitability of FDG based delivery agents within a BNCT context. Instead of developing a theranostic package featuring radiolabeled derivatives, we herein focused on understanding the fundamental properties of such species utilizing their fluorine-19 counterparts.

In the present work, we set out to synthesize a representative library of fluorinated FDG and FDM analogues bearing a carboranylmethyl substituent in either position 4 or 6 and assess their fundamental properties through an in vitro screening program encompassing cytotoxicity, GLUT-affinity, boron delivery capacity, plasma stability and plasma protein binding studies.

The synthetic strategy devised was successful and four representative FDM and FDG derivatives were produced. The structural characterization of the intermediates and end products was performed at a high level and only samples with a purity > 95% were supplied for the in vitro screening program.

To our surprise, the in vitro screening program revealed that the FDM derivative **4**, bearing an *ortho*-carboranylmethyl substituent in position 4, had the best overall profile in terms of cytotoxicity, GLUT-affinity and boron delivery capacity (although the margins are very small across the series of compounds studied). Only glycoconjugate **4** was assessed in the plasma stability and protein binding assays. The plasma stability assay revealed that the stability would be sufficient if the pharmacokinetic profile would resemble that of FDG; however, the pharmacokinetic profile was not assessed in this work and thus it is too early to draw definite conclusions on this subject. The plasma protein binding study revealed a surprisingly strong and long-lasting binding of the glycoconjugate to the proteins, thus diminishing the amounts which would be available in a biological environment. Whether this is a feature of the glycoconjugates, or a consequence of using hydrophobic boron clusters on a general level remains a question. What is certain, however, is that the glycoconjugates studied herein will likely require the development of a separate carrier strategy and a considerable formulation campaign before progression to in vivo studies, or attempting to develop a theranostic package based on the FDG/FDM backbones.

## 4. Materials and Methods

Starting materials as well as all reagents used in the reactions were ordered from a commercial source and used as such (Merck, BLDpharm, Fluorochem, Rahway, NJ, USA). Solvents, bought from commercial sources (Merck, VWR), were purified with the VAC vacuum solvent purification system (VAC, Hawthorne, CA, USA) and additionally dried over 4 Å molecular sieves when deemed necessary. Sensitive (moisture, air, etc.) reactions were carried out under an argon atmosphere. NMR spectra were measured with a Bruker Avance III spectrometer (Bruker, Billerica, MA, USA,) operating at ^1^H: 499.83 MHz, ^13^C: 125.69 MHz, ^11^B: 160.36 MHz and the probe temperature was kept at 25 °C. Each compound synthesized, as well as the final glycoconjugates, were fully characterized using 1D (^1^H, 1D-TOCSY, ^13^C{1H}, and ^11^B{1H}) and 2D (COSY, ed-HSQC, and HMBC) NMR experiments with pulse sequences provided by the instrument manufacturer. The ChemAdder software v. 0.8.7 (Spin Discoveries Ltd., Kuopio, Finland) was used for performing spectral simulations leading to more precise chemical shifts and coupling constants. The coupling constants are reported in Hz and provided when first encountered. Coupling patterns are given as s (singlet), d (doublet), dd (doublet of a doublet), etc. Chemical shifts are expressed on the δ scale (in ppm). The following NMR reference signals were used: TMS (tetramethylsilane), residual chloroform, methanol, or a standard of 15% BF_3_ in CDCl_3_. Certain carbon signals in glycoconjugates containing a fluorine atom are split in two and these are here reported as two separate chemical shifts instead of the mid-point of the signal and the coupling constant. HRMS were recorded in the positive mode with a Bruker Micro Q-TOF (Bruker, Billerica, MA, USA) using electrospray ionization. Note that the majority of HRMS-data in the substrate-specific analytical data section are within the typical 5 ppm error limit and all data are within a 20-ppm error limit. The purity of substrates **1**–**4** was determined to be >95% by qNMR using maleic acid as the internal standard. TLC was recorded on silica gel coated aluminum sheets 60 F254 (Merck), and spots were visualized by spraying with cc. H_2_SO_4_:MeOH (1:5) solution followed by heating. All synthesized compounds were purified by flash chromatography using silica gel 40 as the stationary phase.

The CAL 27 (ATCC CRL-2095) cells used in all assays were acquired from ATCC (Manassas, VA, USA) and cultured in Dulbecco’s Modified Eagle Medium (DMEM) supplemented with L-glutamine (2.0 mM), heat-inactivated fetal bovine serum (10%), and penicillin (50 U/mL)-streptomycin (50 μg/mL) at 37 °C with 5% CO_2_ and 95% relative humidity.

### 4.1. Synthetic Protocols

**General procedure for Fluorination:** To a solution of 3,4,6-tri-*O*-acetyl-D-glucal (1.0 equiv.) in acetone:H_2_O (1:5, 20 mL/1 g of starting material), SelectFluor (1.7 equiv.) was added. The reaction mixture was stirred at room temperature (RT) for 21 h. The mixture was concentrated under reduced pressure and dissolved in DCM (10 mL/1 g of starting material). The mixture was washed with sat. aq. solution of NaHCO_3_ (20 mL/1 g of starting material). The organic phase was separated, and the aqueous phase was extracted with DCM (3 × 10 mL/1 g of starting material). The combined organic phases were dried over anhydrous Na_2_SO_4_, filtered, and concentrated under reduced pressure. The crude product containing was purified by column chromatography, although separation of epimers was not possible at this stage (EtOAc/hexane 1:1).

**General procedure for Acetylation of Free Hydroxyl Groups:** A solution of the partially protected monosaccharide (1.0 equiv.) in dry pyridine (13 mL/g starting material) was treated with acetic anhydride (5.2 equiv.) and DMAP (0.1 equiv.). The reaction was allowed to proceed at RT for 2–3 h. After this period, DCM (15 mL/g starting material) was added to the mixture, and it was subsequently washed with a saturated aqueous solution of NaHCO_3_ (15 mL/g starting material). The organic layer was separated, and the aqueous layer was extracted three times with DCM (3 × 15 mL/g starting material). The combined organic layers were washed with brine (15 mL/g starting material), dried over anhydrous sodium sulfate (Na_2_SO_4_), filtered, and concentrated under reduced pressure. Purification of the crude product was conducted by column chromatography, using a gradient elution system of EtOAc/hexane (1:3 → 1:2 → 1:1) and the epimers could at this stage be separated.

**General procedure for Deacetylation:** To a methanol solution (7 mL/g starting material) of the acetylated monosaccharide (1.0 equiv.), cooled in an ice-water bath, was added NaOMe (1.0 equiv.) until the pH reached approximately 10. The mixture was then stirred at RT for 1–3 h. Dowex 50 H^+^-form resin was added to adjust the pH to neutral. The mixture was filtered and concentrated under reduced pressure. The resulting crude product was purified by column chromatography using a DCM/MeOH gradient (5:1 → 2:1).

**General procedure for Installation of 4,6-*O*-benzylidene Acetal:** To a solution of the deacetylated monosaccharide (1 equiv.) in dry DMF (1 mL/70 mg starting material), *p*-TsOH (10 mol %) and benzaldehyde dimethyl acetal (1.5 equiv.) were added. The mixture was stirred at 60 °C and 200 mbar for 2–3 h, then concentrated. The resulting crude product was purified by column chromatography (DCM/MeOH 12:1).

**General procedure for Alkylation of Free Hydroxyl Groups:** A solution of the partially protected sugar (1.0 equiv.) in dry DMF (2 mL/100 mg starting material) was cooled on an ice-water bath. NaH (2 equiv. per free OH group) was added in portions. The reaction mixture was then allowed to reach RT, and the appropriate alkyl bromide (1.9 equiv. per free OH group) was added. The mixture was stirred for 1.5–3 h, then quenched with MeOH (0.4 mL/1 mmol starting material), diluted with DCM (50 mL/g starting material), and washed with a saturated aqueous NaHCO_3_ (30 mL/g starting material). The organic phase was separated, and the aqueous phase was extracted with DCM (3 × 30 mL/g starting material). The combined organic extracts were washed with brine (30 mL/g starting material), dried over anhydrous Na_2_SO_4_, filtered, and concentrated under reduced pressure. Purification of the crude product was performed using column chromatography (EtOAC/hexane 1:4).

**General procedure for Selective Ring-Opening of the Benzylidene Acetal to yield the 4-OH/6-OBn Substrate:** A solution of the 4,6-O-benzylidene acetal protected monosaccharide (1 equiv.) in DCM (12 mL/g starting material) was prepared, to which Et_3_SiH (5 equiv.) was added. Trifluoroacetic acid (TFA, 5 equiv.) was then added dropwise at 0 °C. The mixture was allowed to warm to RT and stirred for 2 h. It was then diluted with EtOAc (20 mL/0.5 g starting material) and washed sequentially with saturated aqueous NaHCO_3_ (20 mL/0.5 g starting material) and brine (20 mL/0.5 g starting material). The organic layer was separated, dried over anhydrous Na_2_SO_4_, filtered, and concentrated. Purification of the crude product was carried out by column chromatography (EtOAc/hexane 1:4).

**General procedure for Selective Ring-Opening of the Benzylidene Acetal to yield the 4-OBn/6-OH Substrate:** To a solution containing the corresponding 4,6-*O*-benzylidene acetal protected monosaccharide (1 equiv.) in dry DCM (1.5 mL/100 mg of starting material), 1 M borane/tetrahydrofuran complex in THF (5.7 equiv.) was added. The resulting mixture was stirred for 10 min at RT, Cu(OTf)_2_ (0.13 equiv.) was added and stirring was continued for 1.5–3 h. The reaction mixture was brought to 0 °C and the reaction was quenched by the addition of Et_3_N and MeOH. The resulting mixture was concentrated to give the crude product, which was purified by column chromatography (EtOAc/hexane 1:2).

**General procedure for Attachment of Decaborane:** Decaborane (B_10_H_14_, 1.7 equiv.) was dissolved in dry acetonitrile (5 mL/150 mg starting material) and stirred under reflux at 60 °C for 1 h to form the B_10_H_12_·2ACN adduct. In a separate vessel, the propargylated monosaccharide (1.0 equiv.) was dissolved in dry toluene (5 mL/150 mg starting material) and then added to the decaborane solution. This mixture was refluxed at 80 °C for 17 h. Following this, dry MeOH (1.5 mL/150 mg starting material) was added to quench the reaction, and the mixture was refluxed again at 80 °C for 30 min. The reaction was brought to RT and concentrated under reduced pressure. The crude product was then purified by column chromatography (EtOAc/hexane 1:3).

**General procedure for Deprotection of Benzyl Groups:** The protected monosaccharide bearing an *ortho*-carboranylmethyl substituent (1.0 equiv.) was dissolved in a mixture of dry ethyl acetate and methanol (EtOAc/MeOH, 7:1, 1 mL/10–15 mg starting material). Pd/C (10% Pd, 1.0 mass equiv.) was added to the solution. The reaction was carried out in an autoclave under hydrogen pressure (4 bar) for 3–4 h. The mixture was then filtered through Celite, and the Celite was washed with an EtOAc/MeOH solution (5:1). The combined filtrate was concentrated under reduced pressure. The crude product was purified by column chromatography (DCM/MeOH 7:1).

### 4.2. Substrate-Specific Analytical Data

**1,3,4,6-*O*-tetra-*O*-acetyl-2-deoxy-2-fluoro-D-glucopyranose.** Synthesized over two steps, starting from 3,4,6-tri-*O*-acetyl-D-glucal (7.90 g, 29.0 mmol) and SelectFluor (17.49 g, 49.4 mmol) according to the general procedure for fluorination. The intermediate product received was subjected to a further reaction with Ac_2_O (10.30 g, 100.5 mmol) and DMAP (0.28 g, 2.3 mmol) according to the general procedure for acetylation of free hydroxyl groups. This reaction gave the title compound as a colorless oil (2.77 g, 27%, α/β 70:30). TLC: R_f_: 0.60 (EtOAc/hexane 1:1).

*α* anomer: ^1^H NMR (499.83 MHz, CDCl_3_, 25 °C): δ = 6.42 (d, 1H, *J*_1,2_ = 3.9 Hz, H-1), 5.55 (ddd, 1H, *J*_3,2_ = 9.4, *J*_3,4_ = 10.0, *J*_3,F_ = −12.5 Hz, H-3), 5.09 (dd, 1H, *J*_4,5_ = 10.7 Hz, H-4), 4.65 (ddd, 1H, *J*_2,F_ = −48.5 Hz, H-2), 4.28 (dd, 1H, *J*_6a,5_ = 4.2, *J*_6a,6b_ = −12.6 Hz, H-6a), 4.09 (ddd, 1H, *J*_5,6b_ = 2.3 Hz, H-5), 4.07 (dd, 1H, H-6b), 2.21 (s, 3H, 1-OCOC**H_3_**), 2.09 (s, 3H, 3-OCOC**H_3_**), 2.08 (s, 3H, 6-OCOC**H_3_**) and 2.05 (s, 3H, 4-OCOC**H_3_**) ppm.

^13^C NMR (125.69 MHz, CDCl_3_, 25 °C): δ = 170.7 (6-O**C**OCH_3_), 170.2 (3-O**C**OCH_3_), 169.6 (4-O**C**OCH_3_), 168.8 (1-O**C**OCH_3_), 88.5 and 88.3 (C-1), 87.1 and 85.2 (C-2), 70.8 and 70.6 (C-3), 69.6 (C-5), 67.6 and 67.5 (C-4), 61.4 (C-6), 21.0, 20.82, 20.80 and 20.7 (1-OCO**C**H_3_, 3-OCO**C**H_3_, 4-OCO**C**H_3_ and 6-OCO**C**H_3_) ppm.

*β* anomer: ^1^H NMR (499.83 MHz, CDCl_3_, 25 °C): δ = 5.78 (dd, 1H, *J*_1,2_ = 8.2, *J*_1,F_ = 3.1 Hz, H-1), 5.37 (ddd, 1H, *J*_3,2_ = 9.0, *J*_3,4_ = 10.0, *J*_3,F_ = 14.3 Hz, H-3), 5.07 (dd, 1H, *J*_4,5_ = 10.1 Hz, H-4), 4.45 (ddd, 1H, *J*_2,F_ = −50.8 Hz, H-2), 4.30 (dd, 1H, *J*_6a,5_ = 2.1, *J*_6a,6b_ = −12.6 Hz, H-6a), 4.11 (dd, 1H, *J*_6b,5_ = 4.4 Hz, H-6b), 3.86 (ddd, 1H, H-5), 2.18 (s, 3H, 1-OCOC**H_3_**), 2.09 (s, 3H, 3-OCOC**H_3_**), 2.04 (s, 3H, 4-OCOC**H_3_**) and 2.04 (s, 3H, 6-OCOC**H_3_**) ppm.

^13^C NMR (125.69 MHz, CDCl_3_, 25 °C): δ = 170.7 (6-O**C**OCH_3_), 170.0 (3-O**C**OCH_3_), 169.6 (4-O**C**OCH_3_), 168.9 (1-O**C**OCH_3_), 91.4 and 91.3 (C-1), 89.1 and 87.5 (C-2), 72.9 and 72.4 (C-3), 72.9 (C-5), 67.75 and 67.69 (C-4), 60.5 (C-6), 21.2, 20.9, 20.8 and 20.7 (1-OCO**C**H_3_, 3-OCO**C**H_3_, 4-OCO**C**H_3_ and 6-OCO**C**H_3_) ppm.

HRMS *m*/*z*: calcd for C_14_H_19_FO_9_Na [M + Na]^+^, 373.0911; found, 373.0669.

**1,3,4,6-tetra-*O*-acetyl-2-deoxy-2-fluoro-α-D-mannopyranose.** Synthesized over two steps, starting from 3,4,6-tri-*O*-acetyl-D-glucal (7.90 g, 29.0 mmol) and SelectFluor (17.49 g, 49.4 mmol) according to the general procedure for fluorination. The intermediate product received was subjected to a further reaction with Ac_2_O (10.30 g, 100.5 mmol) and DMAP (0.28 g, 2.3 mmol) according to the general procedure for acetylation of free hydroxyl groups. This reaction gave the title compound as a colorless oil (3.68 g, 36%). TLC: R_f_: 0.36 (EtOAc/hexane 1:1).

^1^H NMR (499.83 MHz, CDCl_3_, 25 °C): δ = 6.28 (dd, 1H, *J*_1,2_ = 2.1, *J*_1, F_ = 6.5 Hz, H-1), 5.42 (dd, 1H, *J*_4,3_ = 10.2, *J*_4,5_ = 10.2 Hz, H-4), 5.27 (ddd, *J*_3,2_ = 2.7, *J*_3,F_ = 28.0 Hz, 1H, H-3), 4.76 (ddd, 1H, *J*_2,F_ = −48.7 Hz, H-2), 4.28 (dd, 1H, *J*_6a,5_ = 4.5, *J*_6a,6b_ = −12.5 Hz, H-6a), 4.12 (dd, 1H, *J*_6b,5_ = 2.4 Hz, H-6b), 4.06 (ddd, 1H, H-5), 2.17 (s, 3H, 1-OCOC**H_3_**), 2.12 (s, 3H, 3-OCOC**H_3_**), 2.10 (s, 3H, 6-OCOC**H_3_**) and 2.06 (s, 3H, 4-OCOC**H_3_**) ppm.

^13^C NMR (125.69 MHz, CDCl_3_, 25 °C): δ = 170.8 (6-O**C**OCH_3_), 170.3 (3-O**C**OCH_3_), 169.4 (4-O**C**OCH_3_), 168.2 (1-O**C**OCH_3_), 90.4 and 90.1 (C-1), 86.8 and 85. 3 (C-2), 70.9 (C-5), 69.7 and 69.5 (C-3), 65.3 (C-4), 61.9 (C-6), 21.0, 20.83, 20.82 and 20.7 (1-OCO**C**H_3_, 3-OCO**C**H_3_, 4-OCO**C**H_3_ and 6-OCO**C**H_3_) ppm.

HRMS *m*/*z*: calcd for C_14_H_19_FO_9_Na [M + Na]^+^, 373.0911; found, 373.0894.

**2-deoxy-2-fluoro-D-glucopyranose (7)**. Synthesized from 1,3,4,6-tetra-*O*-acetyl-2-deoxy-2-fluoro-D-glucopyranose (2.76 g, 7.9 mmol) and NaOMe (0.44 g, 8.1 mmol) according to the general procedure for deacetylation. This reaction gave the title compound as a colorless oil (1.35 g, 94%, *α*/*β* 53:47). TLC: R_f_: 0.34 (DCM/MeOH 5:1).

*α* anomer: ^1^H NMR (499.83 MHz, CDCl_3_, 25 °C): δ = 5.29 (d, 1H, *J*_1,2_ = 3.8 Hz, H-1), 4.20 (ddd, 1H, *J*_2,3_ = 9.4, *J*_2,F_ = −50.2 Hz, H-2), 3.92 (ddd, 1H, *J*_3,4_ = 9.0, *J*_3,F_ = 13.0 Hz, H-3), 3.80 (dd, 1H, *J*_6a,5_ = 2.5, *J*_6a,6b_ = −11.8 Hz, H-6a), 3.80 (ddd, 1H, *J*_5,4_ = 10.0, *J*_5,6b_ = 5.1 Hz, H-5), 3.71 (dd, 1H, H-6b) and 3.36 (dd, 1H, H-4) ppm.

^13^C NMR (125.69 MHz, CDCl_3_, 25 °C): δ = 92.9 and 91.4 (C-2), 91.6 and 91.4 (C-1), 7.30 and 72.8 (C-3), 72.8 (C-5), 71.5 (C-4) and 62.2 (C-6) ppm.

*β* anomer: ^1^H NMR (499.83 MHz, CDCl_3_, 25 °C): δ = 4.70 (dd, 1H, *J*_1,2_ = 7.8, *J*_1,F_ = 2.5 Hz, H-1), 3.93 (ddd, 1H, *J*_2,3_ = 8.9, *J*_2,F_ = −51.5 Hz, H-2), 3.87 (dd, 1H, *J*_6a,5_ = 2.0, *J*_6a,6b_ = −12.0 Hz, H-6a), 3.67 (dd, 1H, *J*_6b,5_ = 5.8 Hz, H-6b), 3.60 (ddd, 1H, *J*_3,4_ = 8.9, *J*_3,F_ = 15.5 Hz, H-3), 3.34 (ddd, 1H, *J*_4,5_ = 9.3 Hz, H-5), and 3.33 (dd, 1H, H-4) ppm.

^13^C NMR (125.69 MHz, CDCl_3_, 25 °C): δ = 95.8 and 95.6 (C-1), 95.6 and 94.1 (C-2), 78.1 (C-4), 76.5 and 76.3 (C-3), 71.4 (C-5) and 62.6 (C-6) ppm.

HRMS *m*/*z*: calcd for C_6_H_11_FO_5_Na [M + Na]^+^, 205.0489; found, 205.0528.

**2-deoxy-2-fluoro-D-mannopyranose (11)**. Synthesized from 1,3,4,6-tetra-*O*-acetyl-2-deoxy-2-fluoro-D-mannopyranoside (3.68 g, 10.5 mmol) and NaOMe (0.58 g, 10.7 mmol) according to the general procedure for deacetylation. This reaction gave the title compound as a colorless oil (1.90 g, 99%, *α*/*β* 78:22, only the α-form could be characterized). TLC: R_f_: 0.34 (DCM/MeOH 5:1).

^1^H NMR (499.83 MHz, CDCl_3_, 25 °C): δ = 5.21 (dd, 1H, *J*_1,2_ = 2.2, *J*_1,F_ = 7.4 Hz, H-1), 4.55 (ddd, 1H, *J*_2,3_ = 2.0, *J*_2,F_ = −50.2 Hz, H-2), 3.82 (dd, 1H, *J*_6a,5_ = 2.8, *J*_6a, 6b_ = −11.64 Hz, H-6a), 3.78 (ddd, 1H, *J*_3,4_ = 10.0, *J*_3,F_ = 34.76 Hz, H-3), 3.77 (ddd, 1H, *J*_5,4_ = 9.35, *J*_5,6b_ = 5.6 Hz, H-5), 3.71 (dd, 1H, H-6b) and 3.62 (dd, 1H, H-4) ppm.

^13^C NMR (125.69 MHz, CDCl_3_, 25 °C): δ = 93.2 and 93.0 (C-1), 92.8 and 91.4 (C-2), 74.0 (C-5), 71.5 and 71.3 (C-3), 68.9 (C-4) and 62.8 (C-6) ppm.

HRMS *m*/*z*: calcd for C_6_H_11_FO_5_Na [M + Na]^+^, 205.0489; found, 205.0516.

**4,6-*O*-benzylidene-2-deoxy-2-fluoro-D-glucopyranose**. Synthesized from 2-deoxy-2-fluoro-D-glucopyranose (0.86 g, 4.7 mmol), C_6_H_5_CH(OCH_3_)_2_ (1.22 g, 8.0 mmol) and *p*-TsOH (0.11 g, 0.6 mmol) according to the general procedure for installation of 4,6-*O*-benzylidene acetal. This reaction gave the title compound as a colorless oil (0.92 g, 75%, *α*/*β* 52:48). TLC: R_f_: 0.31 (DCM/MeOH 12:1).

*α* anomer: ^1^H NMR (499.83 MHz, CDCl_3_, 25 °C): δ = 7.52–7.35 (m, 5H, arom. H), 5.53 (s, 1H, C**H**Ph), 5.43 (d, 1H, *J*_1,2_ = 3.9 Hz, H-1), 4.44 (ddd, 1H, *J*_2,3_ = 9.1, *J*_2,F_ = −49.0 Hz, H-2), 4.35 (dd, 1H, *J*_6a,5_ = 5.0, *J*_6a,6b_ = −10.6 Hz, H-6a), 4.33 (ddd, 1H, *J*_3,4_ = 9.2, *J*_3,F_ = 12.7 Hz, H-3), 4.11 (ddd, 1H, *J*_5,4_ = 9.9 Hz, *J*_5,6b_ = 10.2 Hz, H-5), 3.72 (dd, 1H, H-6b) and 3.49 (dd, 1H, H-4) ppm.

^13^C NMR (125.69 MHz, CDCl_3_, 25 °C): δ = 137.0–126.4 (arom. C), 102.2 (**C**HPh), 91.7 and 90.2 (C-2), 91.3 and 91.1 (C-1), 80.84 and 80.77 (C-4), 69.3 and 69.1 (C-3), 68.7 (C-6) and 62.1 (C-5) ppm.

*β* anomer: ^1^H NMR (499.83 MHz, CDCl_3_, 25 °C): δ = 7.52–7.35 (m, 5H, arom. H), 5.53 (s, 1H, C**H**Ph), 4.89 (dd, 1H, *J*_1,2_ = 7.7, *J*_1,F_ = 3.8 Hz, H-1), 4.24 (ddd, 1H, *J*_2,3_ = 8.7, *J*_2,F_ = −50.2 Hz, H-2), 4.30 (dd, 1H, *J*_6a,5_ = 5.1, *J*_6a,6b_ = −10.4 Hz, H-6a), 4.02 (ddd, 1H, *J*_3,4_ = 9.4, *J*_3,F_ = 14.8 Hz, H-3), 3.77 (dd, 1H, *J*_6b,5_ = 10.2 Hz, H-6b), 3.56 (dd, 1H, *J*_4,5_ = 9.5 Hz, H-4) and 3.49 (ddd, 1H, H-5) ppm.

^13^C NMR (125.69 MHz, CDCl_3_, 25 °C): δ = 137.0–126.4 (arom. C), 102.1 (**C**HPh), 95.3 and 95.1 (C-1), 94.8 and 93.3 (C-2), 80.25 and 80.18 (C-4), 72.4 and 72.3 (C-3), 69.1 (C-6) and 66.4 (C-5) ppm.

HRMS *m*/*z*: calcd for C_13_H_15_FO_5_Na [M + Na]^+^, 293.0802; found, 293.0806.

**4,6-*O*-benzylidene-2-deoxy-2-fluoro-D-mannopyranose**. Synthesized from 2-deoxy-2-fluoro-D-mannopyranose (1.08 g, 5.9 mmol), C_6_H_5_CH(OCH_3_)_2_ (1.35 g, 8.9 mmol) and *p*-TsOH (0.12 g, 0.6 mmol) according to the general procedure for installation of 4,6-*O*-benzylidene acetal. This reaction gave the title compound as a colorless oil (1.51 g, 94%, *α*/*β* 81:19). TLC: R_f_: 0.31 (DCM/MeOH 12:1).

*α* anomer: ^1^H NMR (499.83 MHz, CDCl_3_, 25 °C): δ = 7.52–7.35 (m, 5H, arom. H), 5.59 (s, 1H, C**H**Ph), 5.39 (dd, 1H, *J*_1,2_ = 1.8, *J*_1,F_ = 7.8 Hz, H-1), 4.81 (ddd, 1H, *J*_2,3_ = 2.9, *J*_2,F_ = −49.0 Hz, H-2), 4.27 (dd, 1H, *J*_6a,5_ = 5.0, *J*_6a,6b_ = −10.4 Hz, H-6a), 4.21 (ddd, 1H, *J*_3,4_ = 10.5, *J*_3,F_ = 27.7 Hz, H-3), 4.08 (ddd, 1H, *J*_5,6b_ = 10.4 Hz, H-5), 3.92 (dd, 1H, H-4) and 3.81 (dd, 1H, H-6b) ppm.

^13^C NMR (125.69 MHz, CDCl_3_, 25 °C): δ = 129.5–126.4 (arom. C), 102.4 (**C**HPh), 93.2 and 93.0 (C-1), 90.9 and 89.5 (C-2), 79.13 and 79.11 (C-4), 68.9 (C-6), 67.9 and 67.7 (C-3) and 63.6 (C-5) ppm.

*β* anomer: ^1^H NMR (499.83 MHz, CDCl_3_, 25 °C): δ = 7.52–7.35 (m, 5H, arom. H), 5.57 (s, 1H, C**H**Ph), 4.87 (dd, 1H, *J*_1,2_ = 0.03, *J*_1,F_ = 18.7 Hz, H-1), 4.80 (ddd, 1H, *J*_2,3_ = 2.9, *J*_2,F_ = −50.3 Hz, H-2), 4.37 (dd, 1H, *J*_6a,5_ = 5.1, *J*_6a,6b_ = −10.6 Hz, H-6a), 3.94 (ddd, 1H, *J*_3,4_ = 10.2, *J*_3,F_ = 27.9 Hz, H-3), 3.85 (dd, 1H, *J*_6b,5_ = 2.3 Hz, H-6b), 3.83 (dd, 1H, *J*_4,5_ = 11.0 Hz, H-4) and 3.44 (ddd, 1H, H-5) ppm.

^13^C NMR (125.69 MHz, CDCl_3_, 25 °C): δ = 129.5–126.4 (arom. C), 102.3 (**C**HPh), 93.8 and 93.6 (C-1), 91.9 and 90.4 (C-2), 78.18 and78.17 (C-4), 70.4 and 70.3 (C-3), 68.5 (C-6) and 66.9 (C-5) ppm.

HRMS *m*/*z*: calcd for C_13_H_15_FO_5_Na [M + Na]^+^, 293.0802; found, 293.0804.

**Benzyl 3-*O*-benzyl-4,6-*O*-benzylidene-2-deoxy-2-fluoro-D-glucopyranoside (8)**. Synthesized from 4,6-*O*-benzylidene-2-deoxy-2-fluoro-D-glucopyranose (1.90 g, 7.0 mmol), NaH (0.67 g, 28.1 mmol) and BnBr (4.61 g, 26.9 mmol) according to the general procedure for alkylation of free hydroxyl groups. This reaction gave the title compound as a white solid (2.28 g, 72%, *α*/*β* 40:60). TLC: R_f_: 0.32 (EtOAc/Hex 1:4).

*α* anomer: ^1^H NMR (499.83 MHz, CDCl_3_, 25 °C): δ =7.50−7.23 (m, 15H, arom. H), 5.55 (s, 1H, C**H**Ph), 5.12 (d, 1H, *J*_1,2_ = 3.9 Hz, H-1), 4.86 and 4.82 (each d, each 1H, *J* = −11.5 Hz, 3-OC**H_2_**Ph), 4.77 and 4.63 (each d, each 1H, *J* = −12.1 Hz, 1-OC**H_2_**Ph), 4.52 (ddd, 1H, *J*_2,3_ = 9.3, *J*_2,F_ = −48.2 Hz, H-2), 4.23 (dd, 1H, *J*_6a,5_ = 4.9, *J*_6a,6b_ = −10.3 Hz, H-6a), 4.17 (ddd, 1H, *J*_3,4_ = 9.1, *J*_3,F_ = 11.8 Hz, H-3), 3.92 (ddd, 1H, *J*_5,4_ = 9.8, *J*_5,6b_ = 10.3 Hz, H-5), 3.72 (dd, 1H, H-6b) and 3.61 (dd, 1H, H-4) ppm.

^13^C NMR (125.69 MHz, CDCl_3_, 25 °C): δ = 138.4–126.2 (arom. C), 101.5 (**C**HPh), 96.5 and 96.3 (C-1), 91.3 and 89.7 (C-2), 81.33 and 81.26 (C-4), 77.0 and 76.8 (C-3), 74.9 (3-O**C**H_2_Ph), 70.1 (1-O**C**H_2_Ph), 69.0 (C-6) and 62.7 (C-5) ppm.

*β* anomer: ^1^H NMR (499.83 MHz, CDCl_3_, 25 °C): δ = 7.50–7.23 (m, 15H, arom. H), 5.56 (s, 1H, C**H**Ph), 4.93 and 4.69 (each d, each 1H, *J* = −12.0 Hz, 1-OC**H_2_**Ph), 4.85 and 4.83 (each d, each 1H, *J* = −11.8 Hz, 3-OC**H_2_**Ph), 4.65 (dd, 1H, *J*_1,2_ = 7.6, *J*_1,F_ = 4.0 Hz, H-1), 4.42 (ddd, 1H, *J*_2,3_ = 8.4, *J*_2,F_ = −49.6 Hz, H-2), 4.37 (dd, 1H, *J*_6a,5_ = 5.0, *J*_6a,6b_ = −10.5 Hz, H-6a), 3.82 (ddd, 1H, *J*_3,4_ = 9.4, *J*_3,F_ = 15.1 Hz, H-3), 3.79 (dd, 1H, *J*_6b,5_ = 10.0 Hz, H-6b), 3.69 (dd, 1H, *J*_4,5_ = 9.5 Hz, H-4) and 3.41 (ddd, 1H, H-5) ppm.

^13^C NMR (125.69 MHz, CDCl_3_, 25 °C): δ = 138.4–126.2 (arom. C), 101.5 (**C**HPh), 100.2 and 100.0 (C-1), 93.8 and 92.3 (C-2), 80.6 and 80.5 (C-4), 79.1 and 79.0 (C-3), 74.5 (3-O**C**H_2_Ph), 71.3 (1-O**C**H_2_Ph), 68.7 (C-6) and 66.3 (C-5) ppm.

HRMS *m*/*z*: calcd for C_27_H_27_FO_5_Na [M + Na]^+^, 473.1741; found, 473.1715.

**Benzyl 3-*O*-benzyl-4,6-*O*-benzylidene-2-deoxy-2-fluoro-**α**-D-mannopyranoside (12)**. Synthesized from 4,6-*O*-benzylidene-2-deoxy-2-fluoro-D-mannopyranose (1.80 g, 6.7 mmol), NaH (0.67 g, 16.7 mmol) and BnBr (2.88 g, 16.8 mmol) according to the general procedure for alkylation of free hydroxyl groups. This reaction gave the title compound as a white solid (1.96 g, 66%). TLC: R_f_: 0.65 (EtOAc/Hex 1:4).

^1^H NMR (499.83 MHz, CDCl_3_, 25 °C): δ = 7.51–7.24 (m, 15H, arom. H), 5.63 (s, 1H, C**H**Ph), 5.04 (dd, 1H, *J*_1,2_ = 1.8, *J*_1,F_ = 7.9 Hz, H-1), 4.62 and 4.74 (each d, each 1H, *J* = −12.0 Hz, 3-OC**H_2_**Ph), 4.77 (ddd, 1H, *J*_2,3_ = 2.7, *J*_2,F_ = −48.9 Hz, H-2), 4.72 and 4.52 (each d, each 1H, *J* = −11.8 Hz, 1-OC**H_2_**Ph), 4.26 (dd, 1H, *J*_6a,5_ = 4.8, *J*_6a,6b_ = −10.3 Hz, H-6a), 4.13 (dd, 1H, *J*_4,3_ = 10.0, *J*_4,5_ = 9.5 Hz, H-4), 3.90 (ddd, 1H, *J*_5,6b_ = 10.4 Hz, H-5), and 3.85 (dd, 1H, H-6b) ppm.

^13^C NMR (125.69 MHz, CDCl_3_, 25 °C): δ = 138.2–126.2 (arom. C), 101.8 (**C**HPh), 97.7 and 97.4 (C-1), 89.3 and 87.9 (C-2), 78.91 and 78.90 (C-4), 74.6 and 74.4 (C-3), 73.3 (3-O**C**H_2_Ph), 69.7 (1-O**C**H_2_Ph), 68.8 (C-6) and 64.2 (C-5) ppm.

HRMS *m*/*z*: calcd for C_27_H_27_FO_5_Na [M + Na]^+^, 473.1741; found, 473.1721.

**Benzyl 3,4-di-*O*-benzyl-2-deoxy-2-fluoro-D-glucopyranoside (9)**. Synthesized from benzyl 3-*O*-benzyl-4,6-*O*-benzylidene-2-deoxy-2-fluoro-D-glucopyranoside (0.11 g, 0.2 mmol), borane/THF (0.12 g, 1.4 mmol) and Cu(OTf)_2_ (0.01 g, 0.03 mmol) according to the general procedure for selective ring-opening of the benzylidene acetal to yield the 4-OBn/6-OH substrate. This reaction gave the title compound as a colorless oil (0.06 g, 53%, ratio *α*/*β* 40:60). TLC: R_f_: 0.46 (EtOAc/Hex 1:2).

*α* anomer: ^1^H NMR (499.83 MHz, CDCl_3_, 25 °C): δ = 7.40–7.25 (m, 15H, arom. H), 5.09 (d, 1H, *J*_1,2_ = 3.9 Hz, H-1), 4.91 and 4.77 (each d, each 1H, *J* = −11.0 Hz, 3-OC**H_2_**Ph), 4.89 and 4.64 (each d, each 1H, *J* = −11.0 Hz, 4-OC**H_2_**Ph), 4.74 and 4.61 (each d, each 1H, *J* = −12.1 Hz, 1-OC**H_2_**Ph), 4.48 (ddd, 1H, *J*_2,3_ = 12.3, *J*_2,F_ = −49.4 Hz, H-2), 4.16 (ddd, 1H, *J*_3,4_ = 9.0, *J*_3,F_ = 9.6 Hz, H-3), 3.74 (ddd, 1H, *J*_5,4_ = 10.2, *J*_5,6a_ = 3.0, *J*_5,6b_ = 3.9 Hz, H-5), 3.74 (ddd, 1H, *J*_6a,6b_ = −11.9 Hz, *J*_6a,OH_ = 5.3 Hz, H-6a), 3.69 (ddd, 1H, *J*_6b,OH_ = 7.8 Hz, H-6b), 3.57 (dd, 1H, H-4) and 1.57 (dd, 1H, 6-OH) ppm.

^13^C NMR (125.69 MHz, CDCl_3_, 25 °C): δ = 138.5–127.9 (arom. C), 95.9 and 95.7 (C-1), 92.1 and 90.6 (C-2), 80.8 and 80.7 (C-3), 76.7 and 76.6 (C-4), 75.3 (4-O**C**H_2_Ph), 75.3 (3-O**C**H_2_Ph), 71.1 (C-5), 69.9 (1-O**C**H_2_Ph) and 61.8 (C-6) ppm.

*β* anomer: ^1^H NMR (499.83 MHz, CDCl_3_, 25 °C): δ = 7.40–7.25 (m, 15H, arom. H), 4.90 and 4.75 (each d, each 1H, *J* = −11.1 Hz, 3-OC**H_2_**Ph), 4.90 and 4,72 (each d, each 1H, *J* = −12.1 Hz, 1-OC**H_2_**Ph), 4.88 and 4.64 (each d, each 1H, *J* = −11.0 Hz, 4-OC**H_2_**Ph), 4.60 (dd, 1H, *J*_1,2_ = 7.75, *J*_1,F_ = 2.9 Hz, H-1), 4.39 (ddd, 1H, *J*_2,3_ = 8.7, *J*_2,F_ = −50.9 Hz, H-2), 3.85 (ddd, 1H, *J*_6a,5_ = 2.7, *J*_6a,6b_ = −12.1, *J*_6a,OH_ = 6.0 Hz, H-6a), 3.78 (ddd, 1H, *J*_3,4_ = 9.0, *J*_3,F_ = 15.1 Hz, H-3), 3.68 (ddd, 1H, *J*_6b,5_ = 4.6, *J*_6b,OH_ = 7.7 Hz, H-6b), 3.59 (dd, 1H, *J*_4,5_ = 9.7 Hz, H-4), 3.36 (ddd, 1H, H-5) and 1.73 (dd, 1H, 6-OH) ppm.

^13^C NMR (125.69 MHz, CDCl_3_, 25 °C): δ = 138.5–127.9 (arom. C), 99.8 and 99.6 (C-1), 94.2 and 92.7 (C-2), 83.4 and 83.3 (C-3), 76.7 (C-4), 75.3 (C-5), 75.3 (6-O**C**H_2_Ph), 75.0 (3-O**C**H_2_Ph), 71.5 (1-O**C**H_2_Ph) and 62.0 (C-6) ppm.

HRMS *m*/*z*: calcd for C_27_H_29_FO_5_Na [M + Na]^+^, 475.1897; found, 475.1890.

**Benzyl 3,4-di-*O*-benzyl-2-deoxy-2-fluoro-**α**-D-mannopyranoside (13)**. Synthesized from benzyl 3-*O*-benzyl-4,6-*O*-benzylidene-2-deoxy-2-fluoro-D-mannopyranoside (0.11 g, 0.2 mmol), borane/THF (0.12 g, 1.4 mmol) and Cu(OTf)_2_ (0.01 g, 0.03 mmol) according to the general procedure for selective ring-opening of the benzylidene acetal to yield the 4-OBn/6-OH substrate. This reaction gave the title compound as a colorless oil (0.06 g, 53%). TLC: R_f_: 0.26 (EtOAc/Hex 1:3).

^1^H NMR (499.83 MHz, CDCl_3_, 25 °C): δ = 7.40–7.25 (m, 15H, arom. H), 5.04 (dd, 1H, *J*_1,2_ = 2.0, *J*_1,F_ = 7.3 Hz, H-1), 4.93 and 4.67 (each d, each 1H, *J* = −10.9 Hz, 4-OC**H_2_**Ph), 4.76 and 4.72 (each d, each 1H, *J* = −11.6 Hz, 3-OC**H_2_**Ph), 4.75 (ddd, 1H, *J*_2,3_ = 2.0 Hz, *J*_2,F_ = −49.8 Hz, H-2), 4.71 and 4.51 (each d, each 1H, *J* = −11.8 Hz, 1-OC**H_2_**Ph), 3.96 (ddd, 1H, *J*_3,4_ = 9.8, *J*_3,F_ = 29.6 Hz, H-3), 3.93 (ddd, 1H, *J*_4,5_ = 9.7, *J*_4,F_ = −1.0 Hz, H-4), 3.83 (ddd, 1H, *J*_6a,5_ = 2.75, *J*_6a,6b_ = −11.9, *J*_6a,OH_ = 5.2 Hz, H-6a), 3.78 (ddd, 1H, *J*_6b,5_ = 4.3, *J*_6b,OH_ = 8.2 Hz, H-6b), 3.73 (ddd, 1H, H-5) and 1.84 (dd, 1H, 6-OH) ppm.

^13^C NMR (125.69 MHz, CDCl_3_, 25 °C): δ = 138.2–127.9 (arom. C), 96.9 and 96.6 (C-1), 87.7 and 86.3 (C-2), 78.7 amd 78.6 (C-3), 75.6 (4-O**C**H_2_Ph), 74.3 (C-4), 72.4 (3-O**C**H_2_Ph and C-5), 69.6 (1-O**C**H_2_Ph) and 62.1 (C-6) ppm.

HRMS *m*/*z*: calcd for C_27_H_29_FO_5_Na [M + Na]^+^, 475.1897; found, 475.1873.

**Benzyl 3,4-di-*O*-benzyl-2-deoxy-2-fluoro-6-*O*-propargyl-D-glucopyranoside**. Synthesized from benzyl 3,4-di-*O*-benzyl-2-deoxy-2-fluoro-D-glucopyranoside (0.32 g, 0.7 mmol), NaH (0.03 g, 1.3 mmol) and propargyl bromide (0.15 g, 1.3 mmol) according to the general procedure for alkylation of free hydroxyl groups. This reaction gave the title compound as a colorless oil (0.30 g, 85%, *α*/*β* 54:46). TLC: R_f_: 0.56 (EtOAc/hexane 1:3).

*α* anomer: ^1^H NMR (499.83 MHz, CDCl_3_, 25 °C): δ = 7.40–7.25 (m, 15H, arom. H), 5.11 (d, 1H, *J_1,2_* = 3.9 Hz, H-1), 4.90 and 4.76 (each d, each 1H, *J* = −11.1 Hz, 3-OC**H_2_**Ph) 4.87 and 4.69 (each d, each 1H, *J* = −11.8 Hz, 4-OC**H_2_**Ph), 4.75 and 4.62 (each d, each 1H, *J* = −12.1 Hz, 1-OC**H_2_**Ph), 4.52 (ddd, 1H, *J*_2,3_ = 9.3, *J*_2,F_ = −49.5 Hz, H-2), 4.24 (dd, 1H, *J*_CH2a,CH_ = 2.6, *J*_CH2a,CH2b_ = −16.0 Hz, 6-OC**H_2a_**C≡CH), 4.14 (dd, 1H, *J*_CH2b,CH_ = 2.5 Hz, 6-OC**H_2b_**C≡CH), 4.14 (ddd, 1H, *J*_3,4_ = 9.1, *J*_3,F_ = 12.3 Hz, H-3), 3.85 (ddd, 1H, *J*_5,4_ = 10.0, *J*_5,6a_ = 3.5, *J*_5,6b_ = 2.0 Hz, H-5), 3.83 (dd, 1H, *J*_6a,6b_ = −10.4 Hz, H-6a), 3.66 (dd, 1H, H-4), 3.62 (dd, 1H, H-6b) and 2.39 (dd, 1H, 6-OCH_2a_C≡C**H**) ppm.

^13^C NMR (125.69 MHz, CDCl_3_, 25 °C): δ = 138.6–127.8 (arom. C), 96.0 and 95.9 (C-1), 92.7 and 90.5 (C-2), 80.9 and 80.8 (C-3), 79.5 (6-OCH_2_**C**≡CH), 76.8 (C-4), 75.3 (6-OCH_2_C≡**C**H), 75.2 (4-O**C**H_2_Ph), 74.8 (3-O**C**H_2_Ph), 71.0 (1-O**C**H_2_Ph), 70.3 (C-5), 67.9 (C-6) and 58.7 (6-O**C**H_2_C≡CH) ppm.

*β* anomer: ^1^H NMR (499.83 MHz, CDCl_3_, 25 °C): δ = 7.40–7.25 (m, 15H, arom. H), 4.94 and 4.68 (each d, each, 1H, *J* = −11.7 Hz, 1-OC**H_2_**Ph), 4.89 and 4.74 (each d, each 1H, *J* = −11.2 Hz, 3-OC**H_2_**Ph), 4.88 and 4.68 (each d, each 1H, *J* = −10.6 Hz, 4-OC**H_2_**Ph), 4.53 (dd, 1H, *J*_1,2_ = 7.8, *J*_1,F_ = 2.7 Hz, H-1), 4.41 (ddd, 1H, *J_2,3_* = 8.6, *J*_2,F_ = −51.1 Hz, H-2), 4.21 (dd, 1H, *J*_CH2a,CH_ = 2.4, *J*_CH2a,CH2b_ = −15.8 Hz, 6-OC**H_2a_**C≡CH), 4.17 (dd, 1H, *J*_CH2b,CH_ = 2.4 Hz, 6-OC**H_2b_**C≡CH), 3.81 (dd, 1H, *J*_6a,5_ = 4.4, *J*_6a,6b_ = −10.9 Hz, H-6a), 3.78 (dd, 1H, *J*_6b,5_ = 1.9 Hz, H-6b), 3.75 (ddd, 1H, *J*_3,4_ = 8.9, *J*_3,F_ = 15.3 Hz, H-3), 3.65 (dd, 1H, *J*_4,5_ = 9.9 Hz, H-4), 3.46 (ddd, 1H, H-5) and 2.38 (dd, 1H, 6-OCH_2a_C≡C**H**) ppm.

^13^C NMR (125.69 MHz, CDCl_3_, 25 °C): δ = 138.6–127.8 (arom. C), 99.5 and 99.3 (C-1), 94.2 and 92.0 (C-2), 83.5 and 83.4 (C-3), 79.7 (6-OCH_2_**C**≡CH), 76.8 (C-4), 75.3 (6-OCH_2_C≡**C**H), 75.1 (4-O**C**H_2_Ph), 75.0 (C-5), 74.9 (3-O**C**H_2_Ph), 69.9 (1-O**C**H_2_Ph), 68.2 (C-6) and 58.8 (6-O**C**H_2_C≡CH) ppm.

HRMS *m*/*z*: calcd for C_30_H_31_FO_5_Na [M + Na]^+^, 513.2054; found, 513.2196.

**Benzyl 3,4-di-*O*-benzyl-2-deoxy-2-fluoro-6-*O*-propargyl-α-D-mannopyranoside**. Synthesized from benzyl 3,4-di-*O*-benzyl-2-deoxy-2-fluoro-D-mannopyranoside (0.27 g, 0.6 mmol), NaH (0.03 g, 1.2 mmol) and propargyl bromide (0.13 g, 1.1 mmol) according to the general procedure for alkylation of free hydroxyl groups. This reaction gave the title compound as a colorless oil (0.30 g, 81%). TLC: R_f_: 0.56 (EtOAc/hexane 1:3).

^1^H NMR (499.83 MHz, CDCl_3_, 25 °C): δ = 7.40–7.25 (m, 15H, arom. H), 5.06 (dd, 1H, *J*_1,2_ = 1.9, *J*_1,F_ = 7.3 Hz, H-1), 4.90 and 4.70 (each d, each 1H, *J* = −10.8 Hz, 4-OC**H_2_**Ph), 4.75 and 4.71 (each d, each 1H, *J* = −11.6 Hz, 3-OC**H_2_**Ph), 4.73 (ddd, 1H, *J*_2,3_ = 2.4, *J*_2,F_ = −49.7 Hz, H-2), 4.71 and 4.50 (each d, each 1H, *J* = −11.8 Hz, 1-OC**H_2_**Ph), 4.27 (dd, 1H, *J*_CH2a,CH_ = 2.4, *J*_CH2a,CH2b_ = −16.0 Hz, 6-OC**H_2a_**C≡CH), 4.20 (dd, 1H, *J*_CH2b,CH_ = 2.4 Hz, 6-OC**H_2b_**C≡CH), 3.96 (ddd, 1H, *J*_4,3_ = 9.6, *J*_4,5_ = 9.9, *J*_4,F_ = −0.7 Hz, H-4), 3.92 (ddd, 1H, *J*_3,F_ = 31.0 Hz, H-3), 3.89 (dd, 1H, *J*_6a,5_ = 4.4, *J*_6a,6b_ = −10.6 Hz, H-6a), 3.84 (ddd, 1H, *J*_5,6b_ = 2.0 Hz, H-5), 3.72 (dd, 1H, H-6b) and 2.39 (dd, 1H, 6-OCH_2a_C≡C**H**) ppm.

^13^C NMR (125.69 MHz, CDCl_3_, 25 °C): δ = 138.4–127.9 (arom. C), 96.9 and 96.7 (C-1), 87.7 and 86.2 (C-2), 79.8 (6-OCH_2_**C**≡CH), 78.8 and 78.7 (C-3), 75.5 (4-O**C**H_2_Ph), 74.9 (6-OCH_2a_C≡**C**H), 74.3 (C-4), 72.4 (3-O**C**H_2_Ph), 71.7 (C-5), 69.6 (1-O**C**H_2_Ph), 68.3 (C-6) and 58.8 (6-O**C**H_2_C≡CH) ppm.

HRMS *m*/*z*: calcd for C_30_H_31_FO_5_Na [M + Na]^+^, 513.2054; found, 513.2083.

**Benzyl 3,4-di-*O*-benzyl-2-deoxy-2-fluoro-6-*O*-carboranylmethyl-D-glucopyranoside**. Synthesized from benzyl 3,4-di-*O*-benzyl-2-deoxy-2-fluoro-6-*O*-propargyl-D-glucopyranoside (0.29 g, 0.6 mmol) and B_10_H_14_ (0.13 g, 1.1 mmol) according to the general procedure for attachment of decaborane. This reaction gave the title compound as a colorless oil (0.18 g, 49%, *α*/*β* 56:44). TLC: R_f_: 0.56 (EtOAc/hexane 1:3).

*α* anomer: ^1^H NMR (499.83 MHz, CDCl_3_, 25 °C): δ = 7.39–7.22 (m, 15H, arom. H), 5.06 (d, 1H, *J*_1,2_ = 3.9 Hz, H-1), 4.91 and 4.75 (each d, each 1H, *J* = −10.9 Hz, 3-OC**H_2_**Ph), 4.89 and 4.56 (each d, each 1H, *J* = −11.2 Hz, 4-OC**H_2_**Ph), 4.71 and 4.61 (each d, each 1H, *J* = −12.1 Hz, 1-OC**H_2_**Ph), 4.46 (ddd, 1H, *J*_2,3_ = 9.3, *J*_2,F_ = −49.4 Hz, H-2), 4.14 (ddd, 1H, *J*_3,4_ = 8.8, *J*_3,F_ = 12.1 Hz, H-3), 3.88 and 3.83 (each d, each 1H, *J* = −10.7 Hz, 6-OC**H_2_**-carborane), 3.85 (br s, 1H, carborane-C**H**), 3.76 (ddd, 1H, *J*_5,4_ = 10.1, *J*_5,6a_ = 4.2, *J*_5,6b_ = 1.9 Hz, H-5), 3.65 (dd, 1H, *J*_6a,6b_ = −10.7 Hz, H-6a), 3.48 (dd, 1H, H-6b) 3.43 (dd, 1H, H-4) and 2.89–1.44 (br m, 10H, carborane-B**H**) ppm.

^13^C NMR (125.69 MHz, CDCl_3_, 25 °C): δ = 138.2–127.9 (arom. C), 95.8 and 95.6 (C-1), 92.0 and 90.5 (C-2), 80.9 and 80.8 (C-3), 76.6 and 76.5 (C-4), 75.4 (3-O**C**H_2_Ph), 75.2 (6-O**C**H_2_Ph), 72.9 (6-O**C**H_2_-carborane), 72.7 (carborane-**C**), 70.6 (C-6), 70.5 (C-5), 70.0 (1-O**C**H_2_Ph) and 57.6 (carborane-**C**H) ppm.

*β* anomer: ^1^H NMR (499.83 MHz, CDCl_3_, 25 °C): δ = 7.39–7.22 (m, 15H, arom. H), 4.91 and 4.72 (each d, each 1H, *J* = −11.0 Hz, 3-OC**H_2_**Ph), 4.89 and 4.56 (each d, each 1H, *J* = −11.1 Hz, 4-OC**H_2_**Ph), 4.87 and 4.69 (each d, each 1H, *J* = −12.1 Hz, 1-OC**H_2_**Ph), 4.52 (dd, 1H, *J*_1,2_ = 7.8, *J*_1,F_ = 2.8 Hz, H-1), 4.37 (ddd, 1H, *J*_2,3_ = 8.7, *J*_2,F_ = −50.8 Hz, H-2), 3.89 and 3.79 (each d, each 1H, *J* = −10.5 Hz, 6-OC**H_2_**-carborane), 3.78 (br s, 1H, carborane-C**H**), 3.75 (ddd, 1H, *J*_3,4_ = 8.7, *J*_3,F_ = 15.2 Hz, H-3), 3.64 (dd, 1H, *J*_6a,5_ = 4.9, *J*_6a,6b_ = −11.3 Hz, H-6a), 3.62 (dd, 1H, *J*_6b,5_ = 1.9 Hz, H-6b), 3.45 (dd, 1H, *J*_4,5_ = 9.9 Hz, H-4) 3.37 (ddd, 1H, H-5) and 2.89−1.44 (br m, 10H, carborane-B**H**) ppm.

^13^C NMR (125.69 MHz, CDCl_3_, 25 °C): δ = 138.2–127.9 (arom. C), 99.5 and 99.3 (C-1), 94.1 and 92.6 (C-2), 83.4 and 83.3 (C-3), 76.5 and 76.4 (C-4), 75.2 (6-O**C**H_2_Ph), 75.1 (3-O**C**H_2_Ph), 74.8 (C-5), 73.0 (6-O**C**H_2_-carborane), 72.7 (carborane-**C**), 71.2 (1-O**C**H_2_Ph), 70.7 (C-6) and 57.6 (carborane-**C**H) ppm.

^11^B NMR (160.36 MHz, CDCl_3_, 25 °C): δ = −0.53, −2.67, −4.59, −8.84, −11.43 and −12.95 ppm.

HRMS *m*/*z*: calcd for C_30_H_41_B_10_FO_5_Na [M + Na]^+^, 633.3767; found, 633.3821.

**Benzyl 3,4-di-*O*-benzyl-2-deoxy-2-fluoro-6-*O*-carboranylmethyl-α-D-mannopyranoside**. Synthesized from benzyl 3,4-di-*O*-benzyl-2-deoxy-2-fluoro-6-*O*-propargyl-D-mannopyranoside (0.44 g, 0.9 mmol) and B_10_H_14_ (0.16 g, 1.3 mmol) according to the general procedure for attachment of decaborane. This reaction gave the title compound as a colorless oil (0.23 g, 42%). TLC: R_f_: 0.56 (EtOAc/hexane 1:3).

^1^H NMR (499.83 MHz, CDCl_3_, 25 °C): δ = 7.40–7.25 (m, 15H, arom. H), 4.99 (dd, 1H, *J*_1,2_ = 2.3, *J*_1,F_ = 7.2 Hz, H-1), 4.91 and 4.58 (each d, each 1H, *J* = −11.1 Hz, 4-OC**H_2_**Ph), 4.74 and 4.70 (each d, each 1H, *J* = −11.5 Hz, 3-OC**H_2_**Ph), 4.72 (ddd, 1H, *J*_2,3_ = 2.3, *J*_2,F_ = −49.7 Hz, H-2), 4.67 and 4.50 (each d, each 1H, *J* = −11.9 Hz, 1-OC**H_2_**Ph), 3.98 and 3.87 (each d, each 1H, *J* = −10.4Hz, 6-OC**H_2_**-carborane), 3.92 (ddd, 1H, *J*_3,4_ = 9.7, *J*_3,F_ = 29.8 Hz, H-3), 3.88 (br s, 1H, carborane-C**H**), 3.78 (dd, 1H, *J*_4,5_ = 9.8 Hz, H-4), 3.75 (dd, 1H, *J*_6a,5_ = 4.5, *J*_6a,6b_ = −11.3 Hz, H-6a), 3.72 (ddd, 1H, *J*_5,6b_ = 1.7 Hz, H-5), 3.55 (dd, 1H, H-6b) and 2.94–1.44 (br m, 10H, carborane-B**H**) ppm.

^13^C NMR (125.69 MHz, CDCl_3_, 25 °C): δ = 138.1–128.0 (arom. C), 96.7 and 96.5 (C-1), 87.4 and 86.0 (C-2), 78.8 and 78.6 (C-3), 75.4 (1-O**C**H_2_Ph), 73.8 (C-4), 730 (6-O**C**H_2_-carborane), 72.9 (carborane-**C**), 72.4 (3-O**C**H_2_Ph), 72.0 (C-5), 70.9 (C-6), 69.7 (4-O**C**H_2_Ph) and 57.6 (carborane-**C**H) ppm.

^11^B NMR (160.36 MHz, CDCl_3_, 25 °C): δ = −0.49, −2.73, −4.57, −8.90, −11.45 and −13.00 ppm.

HRMS *m*/*z*: calcd for C_30_H_41_B_10_FO_5_Na [M + Na]^+^, 633.3767; found, 633.3790.

**2-deoxy-2-fluoro-6-*O*-carboranylmethyl-D-glucopyranose (1)**. Synthesized from benzyl 3,4-di-*O*-benzyl-2-deoxy-2-fluoro-6-*O*-carboranylmethyl-D-glucopyranoside (0.15 g, 0.2 mmol) and 10% Pd/C (0.16 g, 1.5 mmol) according to the general procedure for deprotection of benzyl groups. This reaction gave the title compound as a colorless oil (0.07 g, 85%, *α*/*β* 50:50). TLC: R_f_: 0.46 (DCM/MeOH 6:1).

*α* anomer: ^1^H NMR (499.83 MHz, CD_3_OD, 25 °C): δ = 5.27 (d, 1H, *J*_1,2_ = 3.8 Hz, H-1), 4.57 (br s, 1H, carborane-C**H**), 4.18 (ddd, 1H, *J*_2,3_ = 9.1, *J*_2,F_ = −50.1 Hz, H-2), 4.05 and 4.01 (each d, each 1H, *J* = −11.0 Hz, 6-OC**H_2_**-carborane), 3.89 (ddd, 1H, *J*_3,4_ = 8.93, *J*_3,F_ = 13.09 Hz, H-3), 3.87 (ddd, 1H, *J*_5,4_ = 10.1, *J*_5,6a_ = 5.0, *J*_5,6b_ = 1.9 Hz, H-5), 3.75 (dd, 1H, *J*_6a,6b_ = −11.3 Hz, H-6a), 3.74 (dd, 1H, H-6b), 3.34 (dd, 1H, H-4) and 3.00–1.39 (br m, 10H, carborane-B**H**) ppm.

^13^C NMR (125.69 MHz, CD_3_OD, 25 °C): δ = 92.7 and 91.2 (C-2), 91.6 and 91.4 (C-1), 75.3 (carborane-**C**), 73.9 (6-O**C**H_2_-carborane), 72.9 and 72.8 (C-3), 72.2 (C-5), 72.1 (C-6), 71.4 and 71.3 (C-4) and 60.6 (carborane-**C**H) ppm.

*β* anomer: ^1^H NMR (499.83 MHz, CD_3_OD, 25 °C): δ = 4.68 (dd, 1H, *J*_1,2_ = 7.8, *J*_1,F_ = 2.6 Hz, H-1), 4.61 (br s, 1H, carborane-C**H**), 4.04 and 4.03 (each d, each 1H, *J* = −11.0 Hz, 6-OC**H_2_**-carborane), 3.90 (ddd, 1H, *J*_2,3_ = 9.1, *J*_2,F_ = −51.3 Hz, H-2), 3.80 (dd, 1H, *J*_6a,5_ = 2.0, *J*_6a,6b_ = −11.4 Hz, H-6a), 3.72 (dd, 1H, *J*_6b,5_ = 5.2 Hz, H-6b), 3.58 (ddd, 1H, *J*_3,4_ = 9.0, *J*_3,F_ = 15.3 Hz, H-3), 3.41 (ddd, 1H, *J*_5,4_ = 10.0 Hz, H-5), 3.31 (dd, 1H, H-4) and 3.00–1.39 (br m, 10H, carborane-B**H**) ppm.

^13^C NMR (125.69 MHz, CD_3_OD, 25 °C): δ = 95.8 and 95.6 (C-1), 95.4 and 94.0 (C-2), 77.0 (C-5), 76.4 and 76.2 (C-3), 75.3 (carborane-**C**), 73.9 (6-O**C**H_2_-carborane), 72.1 (C-6), 71.2 and 71.1 (C-4) and 60.6 (carborane-**C**H) ppm.

^11^B NMR (160.36 MHz, CD_3_OD, 25 °C): δ = −2.27, −4.20, −8.52, −10.69, −11.78 and −12.31 ppm.

HRMS *m*/*z*: calcd for C_9_H_23_B_10_FO_5_Na [M + Na]^+^, 363.2358; found, 363.2391.

**2-deoxy-2-fluoro-6-*O*-carboranylmethyl-D-mannopyranose (3)**. Synthesized from benzyl 3,4-di-*O*-benzyl-2-deoxy-2-fluoro-6-*O*-carboranylmethyl-D-mannopyranoside (0.15 g, 0.2 mmol) and 10% Pd/C (0.16 g, 1.5 mmol) according to the general procedure for deprotection of benzyl groups. This reaction gave the title compound as a colorless oil (0.06 g, 85%, *α*/*β* 80:20). TLC: R_f_: 0.46 (DCM/MeOH 6:1).

*α* anomer: ^1^H NMR (499.83 MHz, CDCl_3_, 25 °C): δ = 5.20 (dd, 1H, *J*_1,2_ = 2.0, *J*_1,F_ = 7.2 Hz, H-1), 4.58 (br s, 1H, carborane-C**H**), 4.56 (ddd, 1H, *J*_2,3_ = 2.6, *J*_2,F_ = −50.0 Hz, H-2), 4.08 and 4.03 (each d, each 1H, *J* = −11.0 Hz, 6-OC**H_2_**-carborane), 3.83 (ddd, 1H, *J*_5,4_ = 10.1, *J*_5,6a_ = 5.0, *J*_5,6b_ = 1.6 Hz, H-5), 3.82 (dd, 1H, *J*_6a,6b_ = −11.2 Hz, H-6a), 3.80 (ddd, 1H, *J*_3,F_ = 30.1 Hz, H-3), 3.73 (dd, 1H, H-6b), 3.65 (dd, 1H, H-4) and 3.01−1.43 (br m, 10H, carborane-B**H**) ppm.

^13^C NMR (125.69 MHz, CD_3_OD, 25 °C): δ = 93.2 and 93.0 (C-1), 92.8 and 91.4 (C-2), 75.2 (carborane-**C**), 73.9 (6-O**C**H_2_-carborane), 73.4 (C-5), 72.3 (C-6), 71.3 and 71.2 (C-3), 68.5 (C-4) and 60.3 (carborane-**C**H) ppm.

*β* anomer: ^1^H NMR (499.83 MHz, CD_3_OD, 25 °C): δ = 4.80 (dd, 1H, *J*_1,2_ = 0.15, *J*_1,F_ = 19.8 Hz, H-1), 4.60 (ddd, 1H, *J*_2,3_ = 3.3, *J*_2,F_ = −51.6 Hz, H-2), 4.58 (br s, 1H, carborane-C**H**), 4.09 and 4.04 (each d, each 1H, *J* = −11.1 Hz, 6-OC**H_2_**-carborane), 3.79 (dd, 1H, *J*_6a,5_ = 2.5, *J*_6a,6b_ = −9.6 Hz, H-6a), 3.77 (ddd, 1H, *J*_3,4_ = 7.0, *J*_3,F_ = 20.3 Hz, H-3), 3.59 (dd, 1H, *J*_4,5_ = 9.6 Hz, H-4), 3.52 (dd, 1H, *J*_6b,5_ = 2.4 Hz, H-6b), 3.35 (ddd, 1H, H-5) and 3.01–1.43 (br m, 10H, carborane-B**H**) ppm.

^11^B NMR (160.36 MHz, CD_3_OD, 25 °C): δ = −2.29, −4.18, −8.55, −10.73, −11.73 and −12.33 ppm.

HRMS *m*/*z*: calcd for C_9_H_23_B_10_FO_5_Na [M + Na]^+^, 363.2358; found, 363.2364.

**Benzyl 3,6-di-*O*-benzyl-2-deoxy-2-fluoro-D-glucopyranoside (10)**. Synthesized from benzyl 3-*O*-benzyl-4,6-*O*-benzylidene-2-deoxy-2-fluoro-D-glucopyranoside (0.96 g, 2.1 mmol), Et_3_SiH (1.29 g, 11.1 mmol) and TFA (1.28 g, 11.2 mmol) according to the general procedure for selective ring-opening of the benzylidene acetal to yield the 4-OH/6-OBn substrate. This reaction gave the title compound as an off-yellow oil (0.86 g, 89%, *α*/*β* 40:60). TLC: R_f_: 0.37 (EtOAc/hexane 1:2).

*α* anomer: ^1^H NMR (499.83 MHz, CDCl_3_, 25 °C): δ = 7.39–7.25 (m, 15 H, arom. H), 5.10 (d, 1H, *J*_1,2_ = 3.9 Hz, H-1), 4.93 and 4.69 (each d, each 1H, *J* = −11.6 Hz, 3-OC**H_2_**Ph), 4.77 and 4.62 (each d, each 1H, *J* = −12.1 Hz, 1-OC**H_2_**Ph), 4.60 and 4.55 (each d, each 1H, *J* = −12.2 Hz, 6-OC**H_2_**Ph), 4.50 (ddd, 1H, *J*_2,3_ = 9.3, *J*_2,F_ = −49.5 Hz, H-2), 3.96 (ddd, 1H, *J*_3,4_ = 8.9, *J*_3,F_ = 14.8 Hz, H-3), 3.82 (ddd, 1H, *J*_5,4_ = 9.8, *J*_5,6a_ = 4.5, *J*_5,6b_ = 3.3 Hz, H-5), 3.68 (dd, 1H, *J*_6a,6b_ = −10.6 Hz, H-6a), 3.67 (ddd, 1H, *J*_4,OH_ = 2.16 Hz, H-4) and 2.46 (d, 1H, 4-OH) ppm.

^13^C NMR (125.69 MHz, CDCl_3_, 25 °C): δ = 138.4–127.8 (arom. C), 95.9 and 95.7 (C-1), 91.8 and 90.3 (C-2), 80.2 and 80.1 (C-3), 75.0 (3-O**C**H_2_Ph), 73.7 (6-O**C**H_2_Ph), 71.0 (C-4), 70.3 (C-5), 69.7 (1-O**C**H_2_Ph) and 69.3 (C-6) ppm.

*β* anomer: ^1^H NMR (499.83 MHz, CDCl_3_, 25 °C): δ = 7.39−7.25 (m, 15 H, arom. H), 4.93 and 4.68 (each d, each 1H, *J* = −11.9 Hz, 1-OC**H_2_**Ph), 4.92 and 4.69 (each d, each 1H, *J* = −11.6 Hz, 3-OC**H_2_**Ph), 4.61 and 4.58 (each d, each 1H, *J* = −12.3 Hz, 6-OC**H_2_**Ph), 4.55 (dd, 1H, *J*_1,2_ = 7.6, *J*_1,F_ = 2.8 Hz, H-1), 4.39 (ddd, 1H, *J*_2,3_ = 8.7, *J*_2,F_ = −51.1 Hz, H-2), 3.77 (dd, 1H, *J*_6a,5_ = 3.6, *J*_6a,6b_ = −10.5 Hz, H-6a), 3.72 (dd, 1H, *J*_6b,5_ = 5.3 Hz, H-6b), 3.65 (ddd, 1H, *J*_4,3_ = 8.8, *J*_4,5_ = 9.6, *J*_4,OH_ = 2.3 Hz, H-4), 3.55 (ddd, 1H, *J*_3,F_ = 14.8 Hz, H-3), 3.45 (ddd, 1H, H-5) and 2.30 (d, 1H, 4-OH) ppm.

^13^C NMR (125.69 MHz, CDCl_3_, 25 °C): δ = 138.4–127.8 (arom. C), 99.4 and 99.2 (C-1), 93.8 and 92.3 (C-2), 82.7 and 82.6 (C-3), 74.6 (3-O**C**H_2_Ph), 74.4 (C-5), 73.8 (6-O**C**H_2_Ph), 70.9 (1-O**C**H_2_Ph), 70.4 (C-4) and 70.0 (C-6) ppm.

HRMS *m*/*z*: calcd for C_27_H_29_FO_5_Na [M + Na]^+^, 475.1897; found, 475.1912.

**Benzyl 3,6-di-*O*-benzyl-2-deoxy-2-fluoro-α-D-mannopyranoside (14)**. Synthesized from benzyl 3-*O*-benzyl-4,6-*O*-benzylidene-2-deoxy-2-fluoro-D-mannopyranoside (0.98 g, 2.2 mmol), Et_3_SiH (1.29 g, 11.1 mmol) and TFA (1.28 g, 11.2 mmol) according to the general procedure for selective ring-opening of the benzylidene acetal to yield the 4-OH/6-OBn substrate. This reaction gave the title compound as a colorless oil (0.82 g, 83%). TLC: R_f_: 0.26 (EtOAc/hexane 1:3).

^1^H NMR (499.83 MHz, CDCl_3_, 25 °C): δ = 7.40–2.24 (m, 15H, arom. H), 5.06 (dd, 1H, *J*_1,2_ = 1.9, *J*_1,F_ = 7.5 Hz, H-1), 4.76 and 4.63 (each d, each 1H, *J* = −11.6, 3-OC**H_2_**Ph), 4.74 and 4.52 (each d, each 1H, *J* = −11.8 Hz, 1-OC**H_2_**Ph), 4.74 (ddd, 1H, *J*_2,3_ = 2.5, *J*_2,F_ = −49.8 Hz, H-2), 4.64 and 4.59 (each d, each 1H, *J* = −12.1 Hz, 6-OC**H_2_**Ph), 4.01 (ddd, 1H, *J*_4,3_ = 9.3, *J*_4,5_ = 9.8, *J*_4,OH_ = 2.2 Hz, H-4), 3.83 (ddd, 1H, *J*_5,6a_ = 6.0, *J*_5,6b_ = 2.9 Hz, H-5), 3.77 (dd, 1H, *J*_6a,6b_ = −12.00 Hz, H-6a), 3.76 (ddd, 1H, *J*_3,F_ = 29.8 Hz, H-3), 3.75 (dd, 1H, H-6b) and 2.54 (d, 1H, 4-OH) ppm.

^13^C NMR (125.69 MHz, CDCl_3_, 25 °C): δ = 138.2–127.8 (arom. C), 96.9 and 96.6 (C-1), 86.8 and 85.4 (C-2), 78.3 and 78.1 (C-3), 73.7 (6-O**C**H_2_Ph), 72.1 (3-O**C**H_2_Ph), 71.5 (C-5), 70.0 (C-6), 69.4 (1-O**C**H_2_Ph) and 67.8 (C-4) ppm.

HRMS *m*/*z*: calcd for C_27_H_29_FO_5_Na [M + Na]^+^, 475.1897; found, 475.1917.

**Benzyl 3,6-di-*O*-benzyl-2-deoxy-2-fluoro-4-*O*-propargyl-D-glucopyranoside**. Synthesized from benzyl 3,6-di-*O*-benzyl-2-deoxy-2-fluoro-D-glucopyranoside (0.83 g, 1.8 mmol), NaH (0.08 g, 3.5 mmol) and propargyl bromide (0.39 g, 3.3 mmol) according to the general procedure for alkylation of free hydroxyl groups. This reaction gave the title compound as a colorless oil (0.75 g, 84%, *α*/*β* 44:56). TLC: R_f_: 0.60 (EtOAc/hexane 1:2).

*α* anomer: ^1^H NMR (499.83 MHz, CDCl_3_, 25 °C): δ = 7.40–7.25 (m, 15H, arom. H), 5.10 (d, 1H, *J*_1,2_ = 4.1 Hz, H-1), 4.87 and 4.74 (each d, each 1H, *J* = −10.9 Hz, 3-OC**H_2_**Ph), 4.75 and 4.60 (each d, each 1H, *J* = −12.2 Hz, 1-OC**H_2_**Ph), 4.63 and 4.55 (each d, each 1H, *J* = −12.0 Hz, 6-OC**H_2_**Ph), 4.48 (ddd, 1H, *J*_2,3_ = 9.4, *J*_2,F_ = −49.1 Hz, H-2), 4.38 (dd, 1H, *J*_CH2a,CH2b_ = −15.3, *J*_CH2a,CH_ = 2.5 Hz, 4-OC**H_2a_**C≡CH), 4.22 (dd, 1H, *J*_CH2b,CH_ = 2.5 Hz, 4-OC**H_2b_**C≡CH), 4.09 (ddd, 1H, *J*_3,4_ = 8.3, *J*_3,F_ = 12.3, H-3), 3.81 (ddd, 1H, *J*_5,4_ = 10.0, *J*_5,6a_ = 2.0, *J*_5,6b_ = 4.1 Hz, H-5), 3.73 (dd, 1H, *J*_6a,6b_ = −12.0 Hz, H-6a), 3.68 (dd, 1H, H-6b), 3.57 (dd, 1H, H-4) and 2.39 (dd, 1H, 4-OCH_2_C≡C**H**) ppm.

^13^C NMR (125.69 MHz, CDCl_3_, 25 °C): δ = 138.4–127.8 (arom. C), 95.8 and 95.6 (C-1), 91.9 and 90.4 (C-2), 80.8 and 80.7 (C-3), 79.9 (4-OCH_2_**C**≡CH), 76.6 (C-4), 75.2 (3-O**C**H_2_Ph), 74.8 (4-OCH_2_C≡**C**H), 73.6 (6-O**C**H_2_Ph), 70.2 (C-5), 69.8 (1-O**C**H_2_Ph), 68.5 (C-6) and 60.2 (4-O**C**H_2_C≡CH) ppm.

*β* anomer: ^1^H NMR (499.83 MHz, CDCl_3_, 25 °C): δ = 7.40–7.25 (m, 15H, arom. H), 4.93 and 4.68 (each d, each 1H, *J* = −12.1 Hz, 1-OC**H_2_**Ph), 4.87 and 4.72 (each d, each 1H, *J* = −11.0 Hz, 3-OC**H_2_**Ph), 4.61 and 4.58 (each d, each 1H, *J* = −11.9 Hz, 6-OC**H_2_**Ph), 4.51 (dd, 1H, *J*_1,2_ = 7.7, *J*_1,F_ = 2.7 Hz, H-1), 4.39 (dd, 1H, *J*_CH2a,CH2b_ = −15.3, *J*_CH2a,CH_ = 2.5 Hz, 4-OC**H_2a_**C≡CH), 4.38 (ddd, 1H, *J*_2,3_ = 7.8, *J*_2,F_ = −51.0 Hz, H-2), 4.28 (dd, 1H, *J*_CH2b,CH_ = 2.4 Hz, 4-OC**H_2b_**C≡CH), 3.83 (dd, 1H, *J*_6a,5_ = 1.9, *J*_6a,6b_ = −10.8 Hz, H-6a), 3.72 (dd, 1H, *J*_6b,5_ = 5.3 Hz, H-6b), 3.71 (ddd, 1H, *J*_3,4_ = 8.5, *J*_1,F_ = 9.3 Hz, H-3), 3.55 (dd, 1H, *J*_4,5_ = 9.8 Hz, H-4), 3.43 (ddd, 1H, H-5) and 2.41 (dd, 1H, 4-OCH_2_C≡C**H**) ppm.

^13^C NMR (125.69 MHz, CDCl_3_, 25 °C): δ = 138.4–127.8 (arom. C), 99.3 and 99.1 (C-1), 94.1 and 92.6 (C-2), 83.3 and 83.2 (C-3), 79.8 (4-OCH_2_**C**≡CH), 76.7 (C-4), 75.0 (3-O**C**H_2_Ph), 74.8 (C-5), 74.6 (4-OCH_2_C≡**C**H), 73.7 (6-O**C**H_2_Ph), 70.9 (1-O**C**H_2_Ph), 69.0 (C-6) and 60.1 (4-O**C**H_2_C≡CH) ppm.

HRMS *m*/*z*: calcd for C_30_H_31_FO_5_Na [M + Na]^+^, 513.2054; found, 513.2225.

**Benzyl 3,6-di-*O*-benzyl-2-deoxy-2-fluoro-4-*O*-propargyl-α-D-mannopyranoside**. Synthesized from benzyl 3,6-di-*O*-benzyl-2-deoxy-2-fluoro-D-mannopyranoside (0.80 g, 1.8 mmol), NaH (0.08 g, 3.4 mmol) and propargyl bromide (0.38 g, 3.2 mmol) according to the general procedure for alkylation of free hydroxyl groups. This reaction gave the title compound as an off-yellow oil (0.75 g, 86%). TLC: R_f_: 0.40 (EtOAc/hexane 1:3).

^1^H NMR (499.83 MHz, CDCl_3_, 25 °C): δ = 7.40–7.24 (m, 15H, arom. H), 5.05 (dd, 1H, *J*_1,2_ = 1.9, *J*_1,F_ = 7.3 Hz, H-1), 4.72 and 4.67 (each d, each 1H, *J* = −11.9 Hz, 3-OC**H_2_**Ph), 4.72 and 4.50 (each d, each 1H, *J* = −11.8 Hz, 1-OC**H_2_**Ph), 4.71 (ddd, 1H, *J*_2,3_ = 2.4, *J*_2,F_ = −49.8 Hz, H-2), 4.67 and 4.58 (each d, each 1H, *J* = −12.1 Hz, 6-OC**H_2_**Ph), 4.42 (dd, 1H, *J*_CH2a,CH2b_ = −15.3, *J*_CH2a,CH_ = 2.4 Hz, 4-OC**H_2a_**C≡CH), 4.26 (dd, 1H, *J*_CH2b,CH_ = 2.4 Hz, 4-OC**H_2b_**C≡CH), 3.88 (ddd, 1H, *J*_3,4_ = 9.4, *J*_3,F_ = 29.7 Hz, H-3), 3.85 (ddd, 1H, *J*_4,5_ = 10.7, *J*_4,F_ = −1.0 Hz, H-4), 3.82 (ddd, 1H, *J*_5,6a_ = 6.0, *J*_5,6b_ = 2.9 Hz, H-5), 3.79 (dd, 1H, *J*_6a,6b_ = −12.4 Hz, H-6a), 3.79 (dd, 1H, H-6b) and 2.39 (dd, 1H, 4-OCH_2_C≡C**H**) ppm.

^13^C NMR (125.69 MHz, CDCl_3_, 25 °C): δ = 138.4–127.7 (arom. C), 96.7 and 96.4 (C-1), 87.4 and 86.0 (C-2), 80.1 (4-OCH_2_**C**≡CH), 78.8 and 78.7 (C-3), 74.5 (4-OCH_2_C≡**C**H), 74.2 (C-4), 73.6 (6-O**C**H_2_Ph), 72.2 (3-O**C**H_2_Ph), 71.6 (C-5), 69.4 (1-O**C**H_2_Ph), 69.1 (C-6) and 60.3 (4-O**C**H_2_C≡CH) ppm.

HRMS *m*/*z*: calcd for C_30_H_31_FO_5_Na [M + Na]^+^, 513.2054; found, 513.2051.

**Benzyl 3,6-di-*O*-benzyl-2-deoxy-2-fluoro-4-*O*-carboranylmethyl-D-glucopyranoside**. Synthesized from benzyl 3,6-di-*O*-benzyl-2-deoxy-2-fluoro-4-*O*-propargyl-D-glucopyranoside (0.74 g, 1.5 mmol) and B_10_H_14_ (0.32 g, 2.6 mmol) according to the general procedure for attachment of decaborane. This reaction gave the title compound as a colorless oil (0.49 g, 53%, *α*/*β* 40:60). TLC: R_f_: 0.37 (EtOAc/hexane 1:3).

*α* anomer: ^1^H NMR (499.83 MHz, CDCl_3_, 25 °C): δ = 7.40–7.25 (m, 15H, arom. H), 5.09 (d, 1H, *J*_1,2_ = 3.9 Hz, H-1), 4.88 and 4.57 (each d, each 1H, *J* = −11.0 Hz, 3-OC**H_2_**Ph), 4.74 and 4.68 (each d, each 1H, *J* = −12.1 Hz, 1-OC**H_2_**Ph), 4.64 and 4.44 (each d, each 1H, *J* = −12.1 Hz, 6-OC**H_2_**Ph), 4.47 (ddd, 1H, *J*_2,3_ = 9.2, *J*_2,F_ = −49.3 Hz, H-2), 4.16 and 3.71 (each d, each 1H, *J* = −10.2 Hz, 4-OC**H_2_**-carborane), 4.01 (ddd, 1H, *J*_3,4_ = 9.0 Hz, *J*_3,F_ = 11.9 Hz, H-3), 3.72 (ddd, 1H, *J*_5,4_ = 10.3, *J*_5,6a_ = 3.2, *J*_5,6b_ = 2.1 Hz, H-5), 3.59 (dd, 1H, *J*_6a,6b_ = −10.8 Hz, H-6a), 3.53 (dd, 1H, H-6b), 3.46 (dd, 1H, H-4), 3.43 (br s, 1H, carborane-C**H**) and 2.80–1.37 (br m, 10H, carborane-B**H**) ppm.

^13^C NMR (125.69 MHz, CDCl_3_, 25 °C): δ = 137.9–128.1 (arom. C), 95.9 and 95.7 (C-1), 92.2 and 90.7 (C-2), 80.2 and 80.1 (C-3), 76.7 (C-4), 75.1 (3-O**C**H_2_Ph), 73.8 (6-O**C**H_2_Ph), 73.4 (4-O**C**H_2_-carborane), 72.3 (carborane-**C**),70.1 (1-O**C**H_2_Ph), 69.7 (C-5), 67.9 (C-6) and 60.5 (carborane-**C**H) ppm.

*β* anomer: ^1^H NMR (499.83 MHz, CDCl_3_, 25 °C): δ = 7.40–7.25 (m, 15H, arom. H), 4.93 and 4.62 (each d, each 1H, *J* = −12.1 Hz, 1-OC**H_2_**Ph), 4.89 and 4.56 (each d, each 1H, *J* = −11.0 Hz, 3-OC**H_2_**Ph), 4.64 and 4.49 (each d, each 1H, *J* = −12.1 Hz, 6-OC**H_2_**Ph), 4.50 (dd, 1H, *J*_1,2_ = 7.8, *J*_1,F_ = 2.8 Hz, H-1), 4.39 (ddd, 1H, *J*_2,3_ = 8.5, *J*_2,F_ = −50.9 Hz, H-2), 4.17 and 3.78 (each d, each 1H, *J* = −10.2 Hz, 4-OC**H_2_**-carborane), 3.66 (dd, 1H, *J*_6a,5_ = 1.8, *J*_6a,6b_ = −11.2 Hz, H-6a), 3.65 (ddd, 1H, *J*_3,4_ = 8.9, *J*_3,F_ = 6.4 Hz, H-3), 3.63 (dd, 1H, *J*_6b,5_ = 3.8 Hz, H-6b), 3.48 (dd, 1H, *J*_4,5_ = 9.8 Hz, H-4), 3.35 (ddd, 1H, H-5), 3.41 (br s, 1H, carborane-C**H**) and 2.80–1.37 (br m, 10H, carborane-B**H**) ppm.

^13^C NMR (125.69 MHz, CDCl_3_, 25 °C): δ = 137.9–128.1 (arom. C), 99.1 and 99.0 (C-1), 94.3 and 92.8 (C-2), 82.8 and 82.6 (C-3), 76.8 (C-4), 74.7 (3-O**C**H_2_Ph), 74.2 (C-5), 73.8 (6-O**C**H_2_Ph), 73.4 (4-O**C**H_2_-carborane), 72.4 (carborane-**C**), 71.0 (1-O**C**H_2_Ph), 68.3 (C-6) and 60.5 (carborane-**C**H) ppm.

^11^B NMR (160.36 MHz, CDCl_3_, 25 °C): δ = −2.63, −4.59, −9.07, −11.50 and −13.10 ppm.

HRMS *m*/*z*: calcd for C_30_H_41_B_10_FO_5_Na [M + Na]^+^, 633.3767; found, 633.3804.

**Benzyl 3,6-di-*O*-benzyl-2-deoxy-2-fluoro-4-*O*-carboranylmethyl-α-D-mannopyranoside**. Synthesized from benzyl 3,6-di-*O*-benzyl-2-deoxy-2-fluoro-4-*O*-propargyl-D-mannopyranoside (0.70 g, 1.4 mmol) and B_10_H_14_ (0.32 g, 2.6 mmol) according to the general procedure for attachment of decaborane. This reaction gave the title compound as a colorless oil (0.47 g, 54%). TLC: R_f_: 0.61 (EtOAc/hexane 1:2).

^1^H NMR (499.83 MHz, CDCl_3_, 25 °C): δ = 7.40–7.27 (m, 15H, arom. H), 5.06 (dd, 1H, *J*_1,2_ = 1.9, *J*_1,F_ = 7.2 Hz, H-1), 4.73 (ddd, 1H, *J*_2,3_ = 2.2, *J*_2,F_ = −50.1 Hz, H-2), 4.71 and 4.52 (each d, each 1H, *J* = −11.9 Hz, 1-OC**H_2_**Ph), 4.69 and 4.48 (each d, each 1H, *J* = −12.0 Hz, 6-OC**H_2_**Ph), 4.69 and 4.50 (each d, each 1H, *J* = −11.2 Hz, 3-OC**H_2_**Ph), 4.18 and 3.76 (each d, each 1H, *J* = −10.3 Hz, 4-OC**H_2_**-carborane), 3.81 (ddd, 1H, *J*_3,4_ = 9.5, *J*_3,F_ = 30.2 Hz, H-3), 3.77 (dd, 1H, *J*_4,5_ = 10.6 Hz, H-4), 3.70 (ddd, 1H, *J*_5,6a_ = 3.6, *J*_5,6b_ = 1.8 Hz, H-5), 3.66 (dd, 1H, *J*_6a,6b_ = −10.8 Hz, H-6a), 3.59 (dd, 1H, H-6b), 3.54 (br s, 1H, carborane-C**H**) and 2.77–1.34 (br m, 10H, carborane-B**H**) ppm.

^13^C NMR (125.69 MHz, CDCl_3_, 25 °C): δ = 138.5–128.1 (arom. C), 96.6 and 96.4 (C-1), 86.5 and 85.1 (C-2), 78.3 and 78.1 (C-3), 74.6 (C-4), 73.7 (6-O**C**H_2_Ph), 73.4 (4-O**C**H_2_-carborane), 72.5 (carborane-**C**), 71.5 (3-O**C**H_2_Ph), 71.0 (C-5), 69.7 (1-O**C**H_2_Ph), 68.3 (C-6) and 58.0 (carborane-**C**H) ppm.

^11^B NMR (160.36 MHz, CDCl_3_, 25 °C): δ = −2.75, −4.62, −9.07, −11.48 and −13.08 ppm.

HRMS *m*/*z*: calcd for C_30_H_41_B_10_FO_5_Na [M + Na]^+^, 633.3767; found, 633.3821.

**2-deoxy-2-fluoro-4-*O*-carboranylmethyl-D-glucopyranose (2)**. Synthesized from benzyl 3,6-di-*O*-benzyl-2-deoxy-2-fluoro-4-*O*-carboranylmethyl-D-glucopyranoside (0.24 g, 0.4 mmol) and 10% Pd/C (0.24 g, 2.3 mmol) according to the general procedure for deprotection of benzyl groups. This reaction gave the title compound as a colorless oil (0.12 g, 90%, *α*/*β* 46:54). TLC: R_f_: 0.71 (DCM/MeOH 5:1).

*α* anomer: ^1^H NMR (499.83 MHz, CD_3_OD, 25 °C): δ = 5.25 (d, 1H, *J*_1,2_ = 3.8 Hz, H-1), 4.67 (br s, 1H, carborane-C**H**), 4.43 and 4.13 (each d, each 1H, *J* = −10.6 Hz, 4-OC**H_2_**-carborane), 4.15 (ddd, 1H, *J*_2,3_ = 9.3, *J*_2,F_ = −50.0 Hz, H-2), 4.03 (ddd, 1H, *J*_3,4_ = 9.4, *J*_3,F_ = 14.0 Hz, H-3), 3.79 (ddd, 1H, *J*_5,4_ = 10.1 Hz, *J*_5,6a_ = 2.2, *J*_5,6b_ = 3.5 Hz, H-5), 3.73 (dd, 1H, *J*_6a,6b_ = −12.1 Hz, H-6a), 3.66 (dd, 1H, H-6b), 3.34 (dd, 1H, H-4) and 2.93–1.41 (br m, 10H, carborane-B**H**) ppm.

^13^C NMR (125.69 MHz, CD_3_OD, 25 °C): δ = 92.9 and 91.4 (C-2), 91.5 and 91.3 (C-1), 79.4 and 79.3 (C-4), 75.0 (carborane-**C**), 74.3 (4-O**C**H_2_-carborane), 72.9 and 72.8 (C-3), 71.3 (C-5), 61.8 (C-6) and 60.6 (carborane-CH) ppm.

*β* anomer: ^1^H NMR (499.83 MHz, CD_3_OD, 25 °C): δ = 5.66 (dd, 1H, *J*_1,2_ = 7.7, *J*_1,F_ = 2.5 Hz, H-1), 4.67 (br s, 1H, carborane-C**H**), 4.31 and 4.12 (each d, each 1H, *J* = −10.6 Hz, 4-OC**H_2_**-carborane), 3.88 (ddd, 1H, *J*_2,3_ = 9.0, *J*_2,F_ = −51.0 Hz, H-2), 3.79 (dd, 1H, *J*_6a,5_ = 1.7, *J*_6a,6b_ = −12.0 Hz, H-6a), 3.77 (ddd, 1H, *J*_3,4_ = 8.8, *J*_3,F_ = 15.0 Hz, H-3), 3.71 (dd, 1H, *J*_6b,5_ = 4.5 Hz, H-6b), 3.36 (ddd, 1H, *J*_5,4_ = 10.8 Hz, H-5), 3.35 (dd, 1H, H-4) and 2.93–1.41 (br m, 10H, carborane-B**H**) ppm.

^13^C NMR (125.69 MHz, CD_3_OD, 25 °C): δ = 95.7 and 95.5 (C-1), 95.5 and 94.2 (C-2), 79.6 and 79.5 (C-4), 76.4 (C-5), 76.3 and 76.2 (C-3), 75.0 (carborane-**C**), 74.4 (4-O**C**H_2_-carborane), 61.9 (C-6) and 60.6 (carborane-**C**H) ppm.

^11^B NMR (160.36 MHz, CD_3_OD, 25 °C): δ = −2.59, −4.13, −8.59, −10.76 and −12.34 ppm.

HRMS *m*/*z*: calcd for C_9_H_23_B_10_FO_5_Na [M + Na]^+^, 363.2358; found, 363.2340.

**2-deoxy-2-fluoro-4-*O*-carboranylmethyl-D-mannopyranose (4)**. Synthesized from benzyl 3,6-di-*O*-benzyl-2-deoxy-2-fluoro-4-*O*-carboranylmethyl-D-mannopyranoside (0.26 g, 0.4 mmol) and 10% Pd/C (0.26 g, 2.4 mmol) according to the general procedure for deprotection of benzyl groups. This reaction gave the title compound as a white solid (0.10 g, 71%, *α*/*β* 84:16). TLC: R_f_: 0.37 (DCM/MeOH 6:1).

*α* anomer: ^1^H NMR (499.83 MHz, CD_3_OD, 25 °C): δ = 5.20 (dd, 1H, *J*_1,2_ = 1.9, *J*_1,F_ = 7.2 Hz, H-1), 4.64 (br s, 1H, carbrane-C**H**), 4.52 (ddd, 1H, *J*_2,3_ = 2.7, *J*_2,F_ = −50.0 Hz, H-2), 4.39 and 4.12 (each d, each 1H, *J* = −10.6 Hz, 4-OC**H_2_**-carborane), 3.93 (ddd, 1H, *J*_3,4_ = 9.5, *J*_3,F_ = 30.7 Hz, H-3), 3.77 (ddd, 1H, *J*_5,4_ = 9.8, *J*_5,6a_ = 2.1, *J*_5,6b_ = 4.1 Hz, H-5), 3.75 (dd, 1H, *J*_6a,6b_ = −12.0 Hz, H-6a), 3.71 (dd, 1H, H-6b), 3.59 (dd, 1H, H-4) and 3.02–1.38 (br m, 10H, carborane-B**H**) ppm.

^13^C NMR (125.69 MHz, CD_3_OD, 25 °C): δ = 93.3 and 92.8 (C-2), 93.1 and 91.9 (C-1), 77.6 (C-4), 75.0 (carborane-**C**), 74.5 (4-O**C**H_2_-carborane), 72.4 (C-5), 71.6 and 71.5 (C-3), 62.1 (C-6) and 60.5 (carborane-**C**H) ppm.

*β* anomer: ^1^H NMR (499.83 MHz, CD_3_OD, 25 °C): δ = 4.80 (dd, 1H, *J*_1,2_ = 0.1, *J*_1,F_ = 20.0 Hz, H-1), 4.64 (br s, 1H, carborane-C**H**), 4.55 (ddd, 1H, *J*_2,3_ = 2.8, *J*_2,F_ = −51.3 Hz, H-2), 4.40 and 4.11 (each d, each 1H, *J* = −10.6 Hz, 4-OC**H_2_**-carborane), 3.82 (dd, 1H, *J*_6a,5_ = 2.1, *J*_6a,6b_ = −12.1 Hz, H-6a), 3.77 (dd, 1H, *J*_3,4_ = 10.0, H-3), 3.69 (dd, 1H, *J*_6b,5_ = 4.4 Hz, H-6b), 3.54 (dd, 1H, *J*_4,5_ = 9.2 Hz, H-4), 3.32 (ddd, 1H, H-5) and 3.02–1.38 (br m, 10H, carborane-B**H**) ppm.

^13^C NMR (125.69 MHz, CD_3_OD, 25 °C): δ = 94.3 and 94.2 (C-1), 93.4 and 92.5 (C-2), 79.5 (carborane-**C**), 77.2 (C-4),76.6 (C-5), 75.0 (4-O**C**H_2_-carborane), 74.4 and 74.2 (C-3), 62.1 (C-6) and 60.6 (carborane-**C**H) ppm.

^11^B NMR (160.36 MHz, CD_3_OD, 25 °C): δ = −2.59, −4.13, −8.59, −10.76 and −12.34 ppm.

HRMS *m*/*z*: calcd for C_9_H_23_B_10_FO_5_Na [M + Na]^+^, 363.2358; found, 363.2353.

### 4.3. Cytotoxicity Studies

An opaque 96-well plate was used for seeding the CAL 27 cells at a density of 5000 cells per well and left incubated overnight. Stock solutions of the glycoconjugates were prepared in DMSO. The samples studied were prepared from the stock solutions by diluting in cell culture media. The cell culture media containing glycoconjugates **1**–**4** and BSH at four concentrations (5, 25, 50, 125, and 250 μM), 1% *v*/*v* DMSO (negative control for cytotoxicity), or 1% *v*/*v* Triton X-100 (positive control for cytotoxicity) were incubated with the cells for 6 and 24 h at 37 °C, 5% CO_2_ atmosphere, and 95% relative humidity. At predetermined time points, cells were washed twice with 1× DPBS (pH 7.4). The cell viability assay was carried out by adding 50 μL of 1× HBSS and CellTiter-Glo reagent (1:1) to each well and the cell lysis was performed at RT without the presence of light. The luminescence signal of viable cells was measured (n = 4) using the Synergy H1 Hybrid multimode microplate reader (BioTek, Winooski, VT, USA). The following step was the quantification of the total protein content per sample. For this measurement, the Pierce colorimetric bicinchoninic acid (BCA) protein assay (Thermo Fisher Scientific, Waltham, MA, USA) was used following the protocol in our previously published work [7]. Briefly, 25 μL aliquot of the sample from each well was transferred to a new transparent 96-well plate. Then, BCA working reagent (200 μL) was added and the plates were covered in aluminum foil and incubated at 37 °C for 30 min. The absorbance was recorded at 562 nm using the microplate reader (n = 3). BSA standard curve (conc. range 0–2000 μg/mL) was used to determine the protein concentration.

### 4.4. Affinity Studies

The GLUT1 affinity assay was conducted following our previously established protocol [7]. Stock solutions of the glycoconjugates were prepared in DMSO. From the stock solutions, the samples employed in the study were prepared by diluting the stock solutions in glucose-free HBSS-buffer to reach concentrations of 0.1–1800 μM. The cells were incubated at RT for 5 min with glycoconjugates **1**–**4**, BSH or BPA over the concentration range given above. The glucose-free HBSS buffer as such was used as a blank control. In more detail, the solutions included 1.8 μM (0.1 mCi/mL) of [^14^C]-D-glucose in 250 μL of glucose-free HBSS (Hanks’ balanced salt solution). Ice-cold buffer was used for quenching. Cell lysis was performed using 0.1 M NaOH (250 μL) and then mixed with an emulsifier safe cocktail (1.0 mL) (PerkinElmer, Waltham, MA, USA). The radioactivity was measured with a liquid scintillation counter (MicroBeta2 counter, PerkinElmer, Waltham, MA, USA), and the IC_50_ values were calculated through nonlinear regression analysis.

### 4.5. Boron Uptake Studies

The boron uptake studies were performed according to our earlier reported protocol [7]. The samples used in the boron uptake studies were prepared as described above for the affinity studies. The cells were preincubated. Glycoconjugates **1**–**4** at various concentrations (10–400 μM in 250 μL of HBSS) were added, and incubation continued for 5, 30, and 120 min at RT. The reactions were quenched at the designated time points using an ice-cold buffer. Washing and lysis were performed as previously described. A combined sample was prepared by pooling cell lysate from four wells into an Eppendorf tube. The combined sample was centrifuged at +4 °C. From the supernatant of each sample, 800 μL was taken and digested in 1.0 mL of HNO_3_ (TraceMetal grade, Fisher Chemical, Fisher Scientific, Hampton, NH, USA) for 24 h. Milli-Q water (USF Elga Purelab Ultra, Veolia Water Technologies, High Wycombe, UK) was added to bring the sample volume to 10 mL, and the boron content was measured in triplicates using ICP-MS. More details on the ICP method are available in our earlier work. Data analysis was conducted using PerkinElmer Syngistix™ Data Analysis Software for ICP v 1.1 (PerkinElmer, Waltham, MA, USA), and statistical analysis was performed with GraphPad Prism v.5.03 software (GraphPad Software, San Diego, CA, USA).

### 4.6. Plasma Stability Studies

10 mM working solutions of the test compound was prepared in DMSO and a 1 mM working solution of the control compound propantheline was prepared in acetonitrile. 4 µL of working solutions was spiked to 796 µL of pre-incubated plasma to reach a final concentration of 50 µM or 5 µM. The final concentration of solvent was 0.5%. 50 µL aliquots of the spiked plasma were added into new tubes for 1, 4, 8 and 24 h and incubated at 37 °C water bath with shaking at 60 rpm. The assay was performed in duplicate. The reaction was stopped by adding 300 µL of RT quench solution (acetonitrile containing internal standards (IS, 100 nM Alprazolam, 500 nM Labetalol, and 2 µM Ketoprofen)) to the spiked plasma samples at the appointed time points. Time 0 samples were prepared by adding 50 µL of the spiked plasma to new tubes containing 300 µL of RT quench solution and vortexed for 5 min. Samples in the plate were centrifuged at 3220× *g* for 30 min at 4 °C to precipitate protein and then 100 μL of the supernatant was transferred to a new 96-well plate with 100 μL water for LC-MS/MS analysis. All calculations were carried out using Microsoft Excel v. 2407. Peak area ratios were determined from extracted ion chromatograms. Percent compounds remaining at each time point were calculated by the following equation:

Remaining Percentage t h (%) = Peak Area Ratio t h/Peak Area Ratio 0 h × 100

where Peak Area Ratio t h is the peak area ratio of control and test compound at t h; Peak Area Ratio 0 h is the peak area ratio of control and test compound at zero time point.

### 4.7. Plasma Protein Binding Studies

The working solutions of test compounds were prepared in DMSO at the concentrations of 10 mM and the working solution of control compound ketoconazole was prepared in DMSO at the concentration of 200 μM. 3 μL of the working solutions were removed to mix with 597 μL of plasma to achieve a final concentration of 1 μM or 50 μM (0.5% DMSO). The plasma samples were vortexed thoroughly. A total of 50 µL of the spiked plasma sample was transferred to 50 μL of blank buffer, and then either incubated for 6 h (37 °C with 5% CO_2_) or immediately quenched by the addition of 300 μL of RT quench solution (acetonitrile containing internal standards (IS, 200 nM labetalol, 100 nM tolbutamide and 100 nM ketoprofen) to precipitate protein and the mixture was vortex for 5 min.

The dialysis membranes were soaked in ultrapure water for 60 min to separate strips, then in 20% ethanol for 20 min, and finally in dialysis buffer for 20 min. The dialysis apparatus was assembled according to the manufacturer’s instructions. Each cell was filled with 120 μL of spiked plasma sample and dialyzed against equal volume of dialysis buffer (PBS). The assay was performed in duplicate. The dialysis plate was sealed and incubated in an incubator at 37 °C with 5% CO_2_ at 100 rpm for 6 h. At the end of incubation, the seal was removed and 50 μL of samples from both buffer and plasma chambers were transferred to wells of a 96-well plate. 50 μL of blank plasma was added to each buffer sample and an equal volume of PBS was supplemented to the collected plasma sample. 300 μL of room temperature quench solution (acetonitrile containing internal standards (IS, 200 nM labetalol, 100 nM tolbutamide and 100 nM ketoprofen) was added to precipitate protein. Samples in the plate were vortexed for 5 min and centrifuged at 3220× *g* for 30 min at 4 °C 100 μL of the supernatant was transferred to a new 96-well plate with 100 μL water for LC-MS/MS analysis. All calculations were carried out using Microsoft Excel. Determination of the concentrations of test compound and control compound in the buffer and plasma chambers was performed based on the peak area ratios. The percentages of the test compound and control compound bound were calculated as follows:

% Fu = (Peak Area Ratio buffer chamber/Peak Area Ratio plasma chamber) × 100

% Bound = 100 − % Fu

% Recovery = (Peak Area Ratio buffer chamber + Peak Area Ratio plasma chamber)/Peak Area Ratio total sample × 100

% Remaining T6 (%) = Peak Area Ratio T6/Peak Area Ratio total sample × 100

Peak Area Ratio buffer chamber: the concentration for free fraction; Peak Area Ratio plasma chamber: the concentration for both free and bound fraction; Peak Area Ratio total sample: the concentration for starting sample before incubation; Peak Area Ratio T6: the concentration for starting sample after 6 h incubation.

## Figures and Tables

**Table 1:** Comparison of IC_50_ values of compounds **1**–**4**, D-glucose, and some of the glycoconjugates previously synthesized in our research program. The incubation time in the cis-inhibition assay was 5 min.

Compound	Molecular Structure	IC_50_
**1**	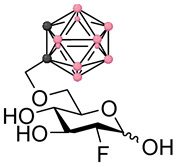	78.26 μM
**2**	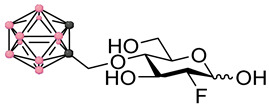	57.54 μM
**3**	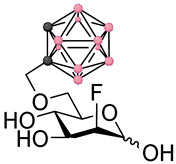	62.62 μM
**4**	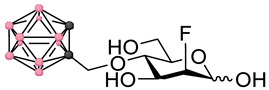	59.54 μM
**6-*O*-Cb-** **D** **-Glc**	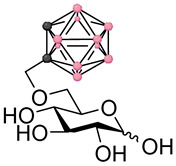	43.96 μM [7]
**6-*O*-Cb-** **D** **-Man**	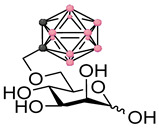	78.08 μM [10]
**D** **-Glc**	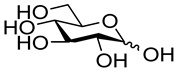	>1 mM

## Data Availability

The ^1^H, ^13^C, and ^11^B NMR spectra are available in the Supporting Information. Other data can be made available separately upon request.

## Supplementary Materials

The following supporting information can be downloaded at: https://www.mdpi.com/article/10.3390/molecules29174263/s1, The supporting information contains representative ^1^H, ^11^B, and ^13^C NMR spectra for all compounds.
