# Supplementary material for: Towards New Delivery Agents for Boron Neutron Capture Therapy: Synthesis and In Vitro Evaluation of a Set of Fluorinated Carbohydrate Derivatives
Source: Molecules. 2024 Sep 9;29(17):4263. doi: 10.3390/molecules29174263 (PMC11397260; doi:10.3390/molecules29174263)
Supplement: Supplementary file 1 [file molecules-29-04263-s001.zip › molecules-3136262-supplementary.pdf]

Supporting information

Towards New Delivery Agents for Boron Neutron Capture Therapy: Synthesis and *In Vitro* Evaluation of a Set of Fluorinated Carbohydrate Derivatives

Jelena Matović,[1,ϕ] Juulia Järvinen,[2,ϕ] Iris K. Sokka,[1] Surachet Imlimthan,[1] Olli Aitio,[3] Mirkka Sarparanta,[1] Jarkko Rautio[2] and Filip S. Ekholm[1*]

[1] Department of Chemistry, University of Helsinki, Finland, P.O. Box 55, FI-00014 Helsinki, Finland

[2] School of Pharmacy, University of Eastern Finland, P.O. Box 1627, FI-70211 Kuopio, Finland

[3] Glykos Finland Ltd., Viikinkaari 6, FI-00790 Helsinki, Finland

[ϕ] Equal contributions

Corresponding author contact: filip.ekholm@helsinki.fi

Contents:

1. Representative NMR spectra of synthesized compounds……………………………………………S2

1. Representative NMR spectra of synthesized compounds

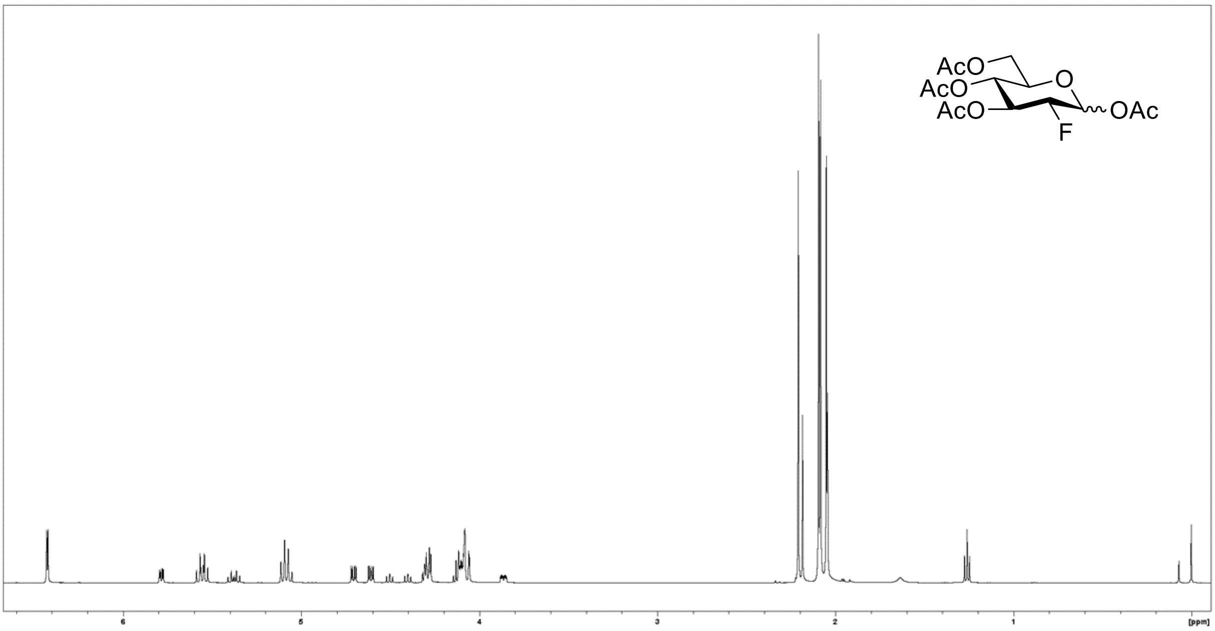

Figure S1. ^{1}H NMR spectrum of **1,3,4,6-tetra-*O*-acetyl-2-deoxy-2-fluoro-D-glucopyranose** (499.83 MHz, 25 °C, $CDCl_3$).

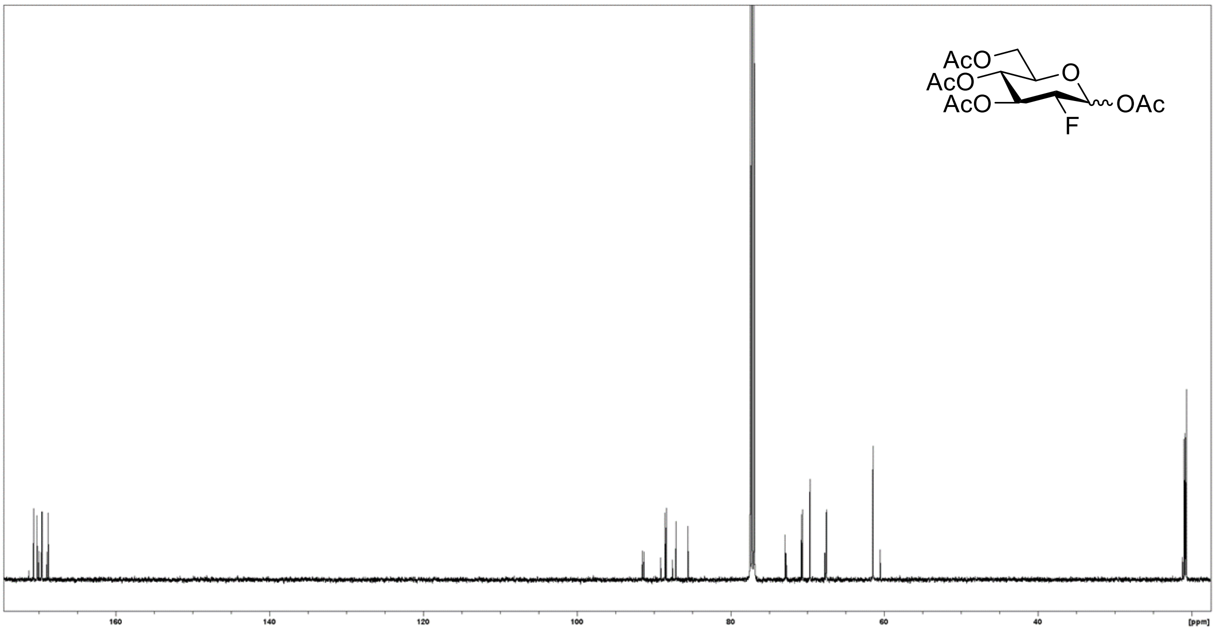

Figure S2. $^{13}C\{^{1}H\}$ NMR spectrum of **1,3,4,6-tetra-*O*-acetyl-2-deoxy-2-fluoro-D-glucopyranose** (125.69 MHz, 25 °C, $CDCl_3$).

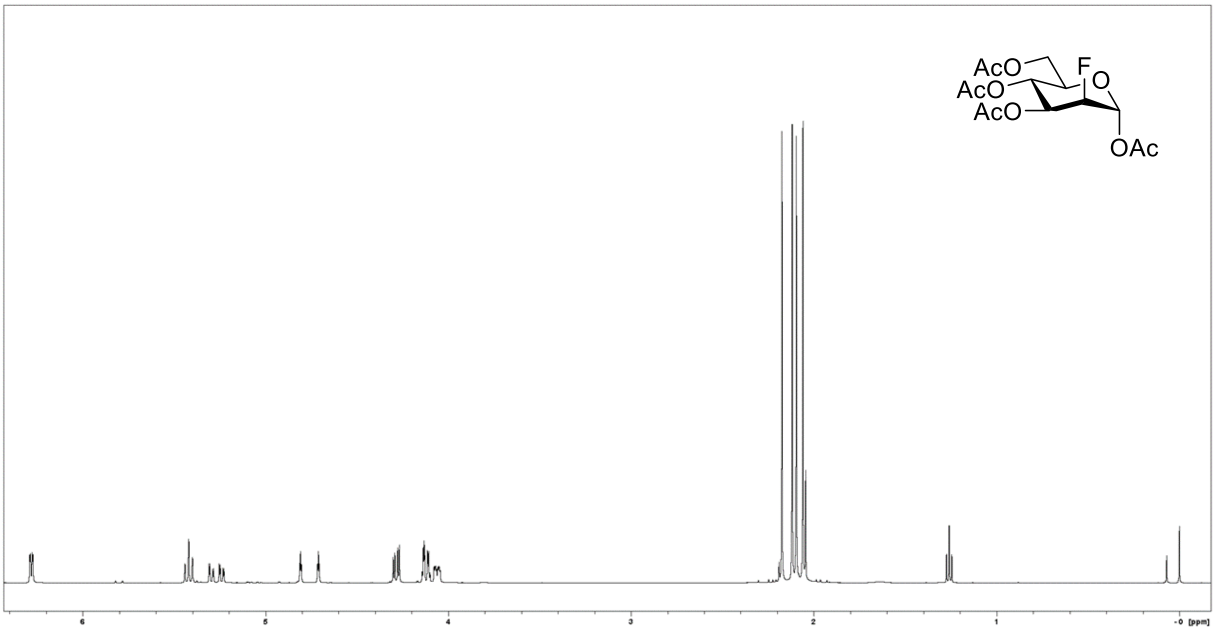

Figure S3. ^{1}H NMR spectrum of **1,3,4,6-tetra-*O*-acetyl-2-deoxy-2-fluoro-α-D-mannopyranose** (499.83 MHz, 25 °C, $CDCl_3$).

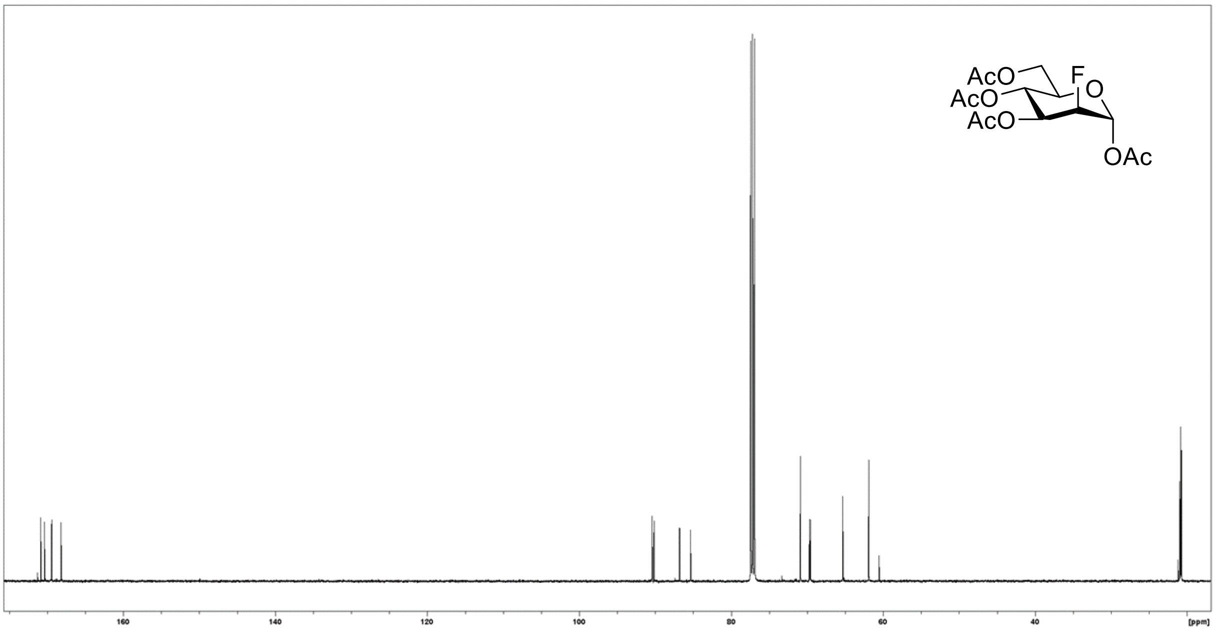

Figure S4. ^{13}C{^{1}H} NMR spectrum of **1,3,4,6-tetra-*O*-acetyl-2-deoxy-2-fluoro-α-D-mannopyranose** (125.69 MHz, 25 °C, $CDCl_3$).

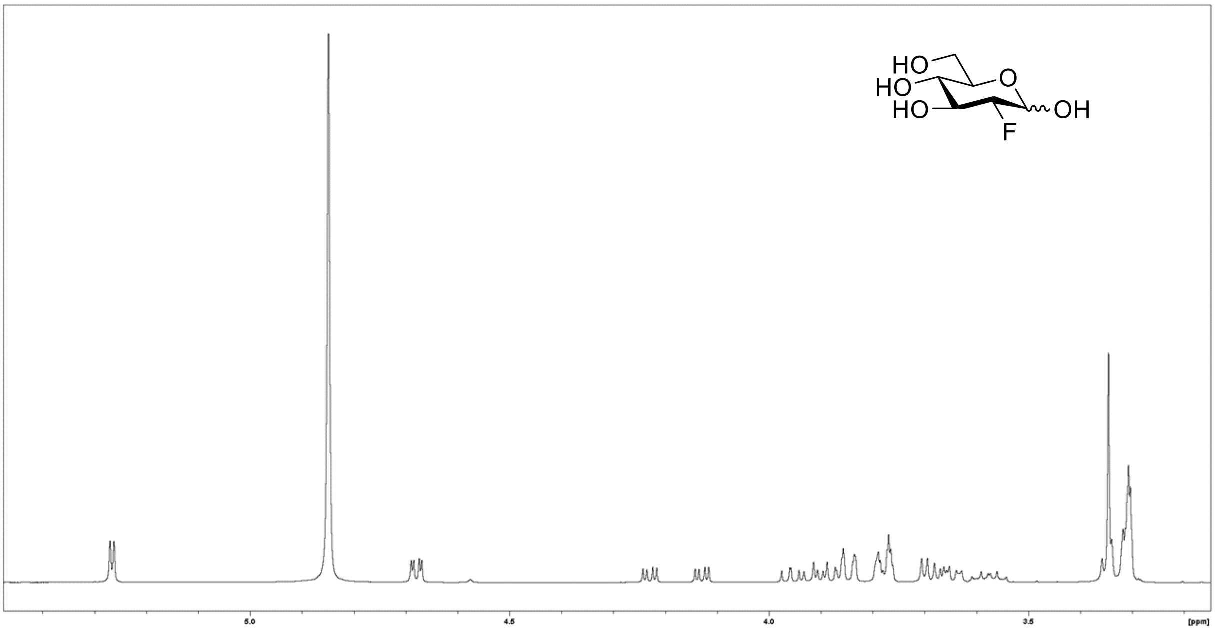

Figure S5. ^{1}H NMR spectrum of **2-deoxy-2-fluoro-D-glucopyranose (7)** (499.83 MHz, 25 °C, CD_3OD).

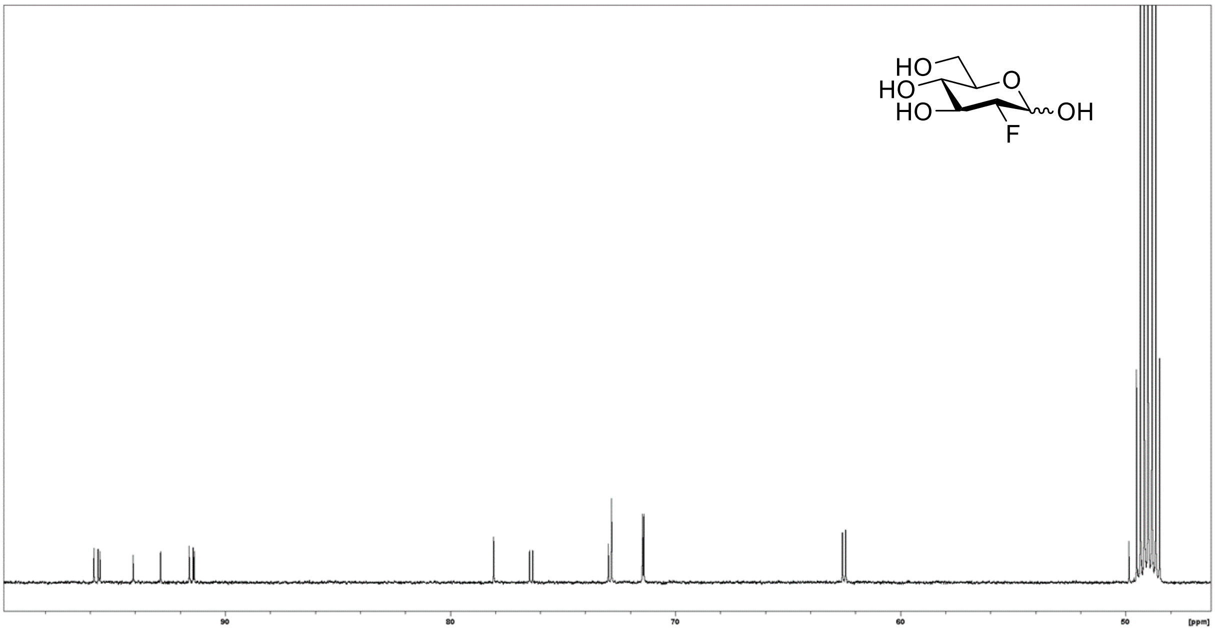

Figure S6. $^{13}C\{^{1}H\}$ NMR spectrum of **2-deoxy-2-fluoro-D-glucopyranose (7)** (125.69 MHz, 25 °C, CD_3OD).

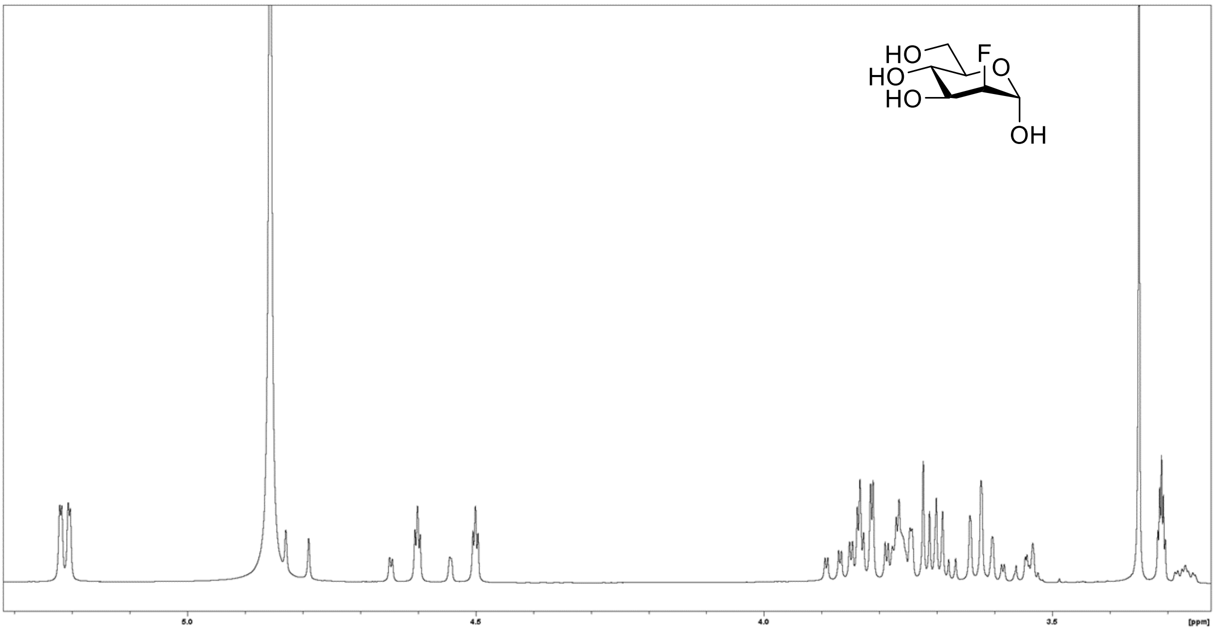

Figure S7. ^{1}H NMR spectrum of **2-deoxy-2-fluoro-α-D-mannopyranose (11)** (499.83 MHz, 25 °C, CD_3OD).

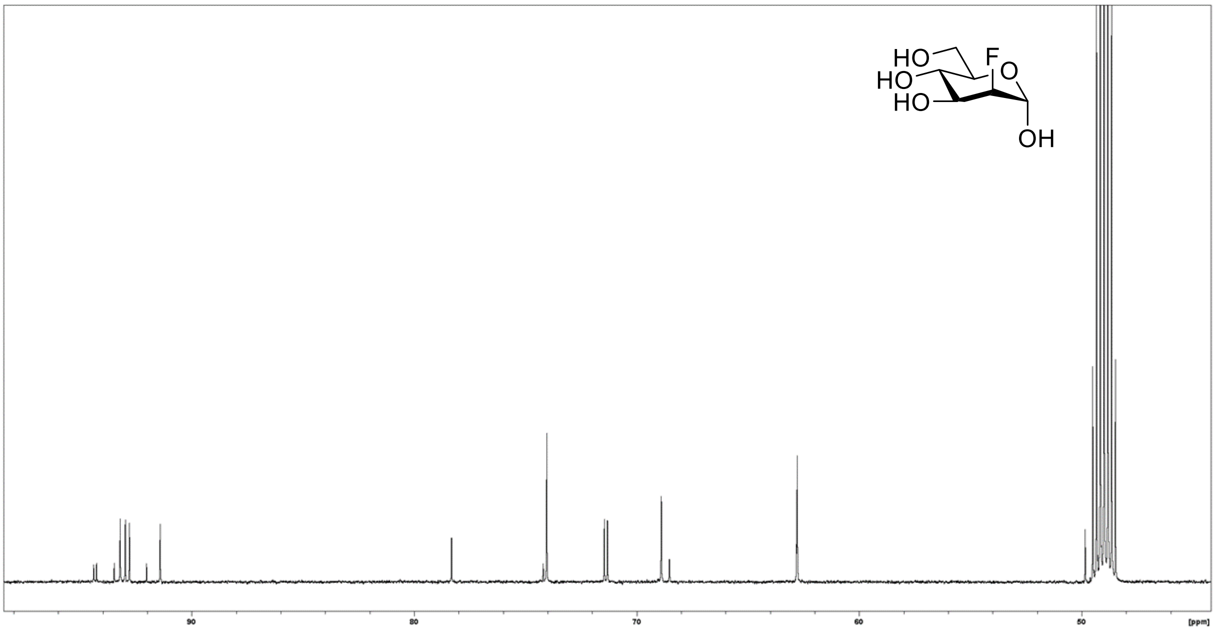

Figure S8. $^{13}C\{^1H\}$ NMR spectrum of **2-deoxy-2-fluoro-α-D-mannopyranose (11)** (125.69 MHz, 25 °C, CD_3OD).

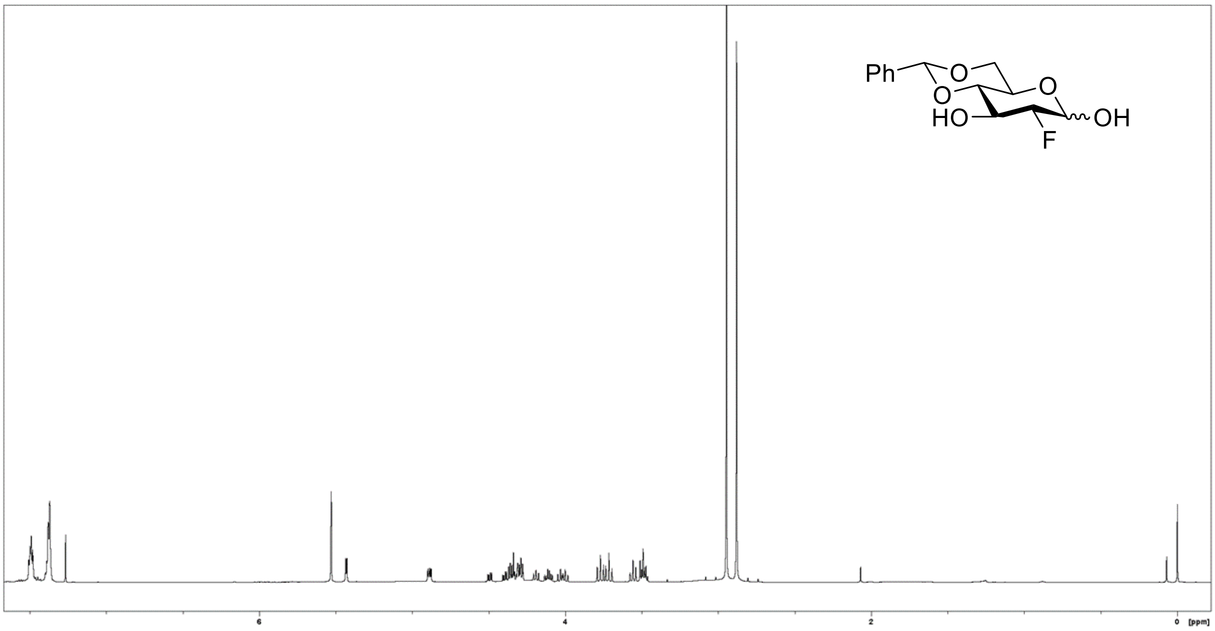

Figure S9. ^{1}H NMR spectrum of **4,6-*O*-benzylidene-2-deoxy-2-fluoro-D-glucopyranose** (499.83 MHz, 25 °C, $CDCl_3$).

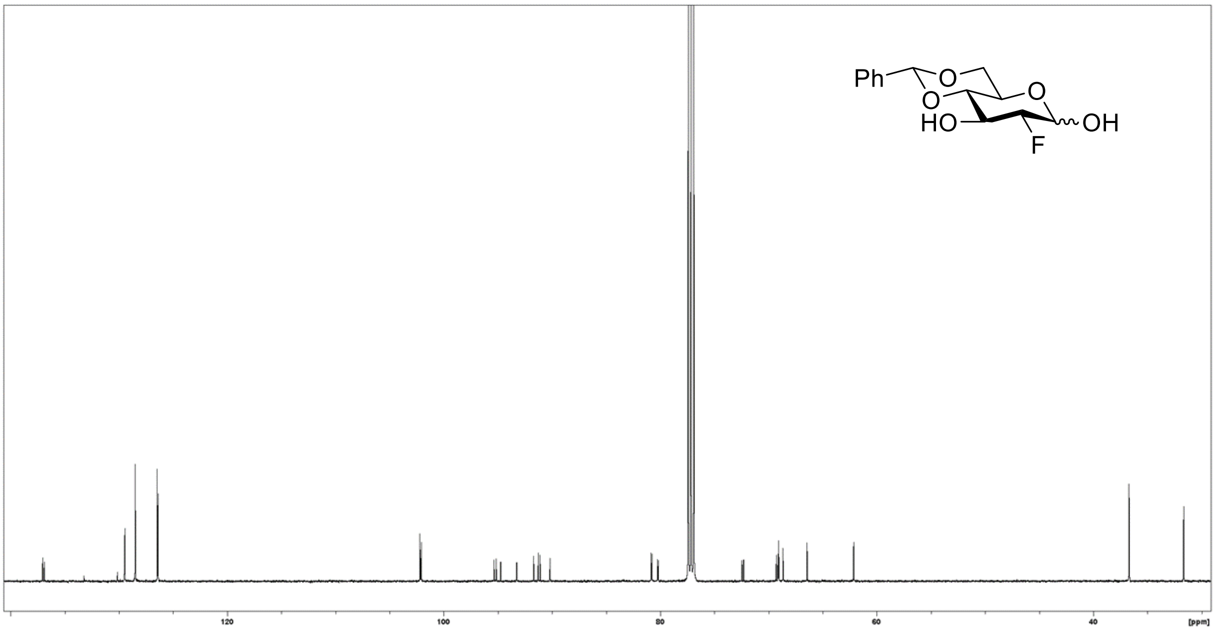

Figure S10. $^{13}C\{^{1}H\}$ NMR spectrum of **4,6-*O*-benzylidene-2-deoxy-2-fluoro-D-glucopyranose** (125.69 MHz, 25 °C, $CDCl_3$).

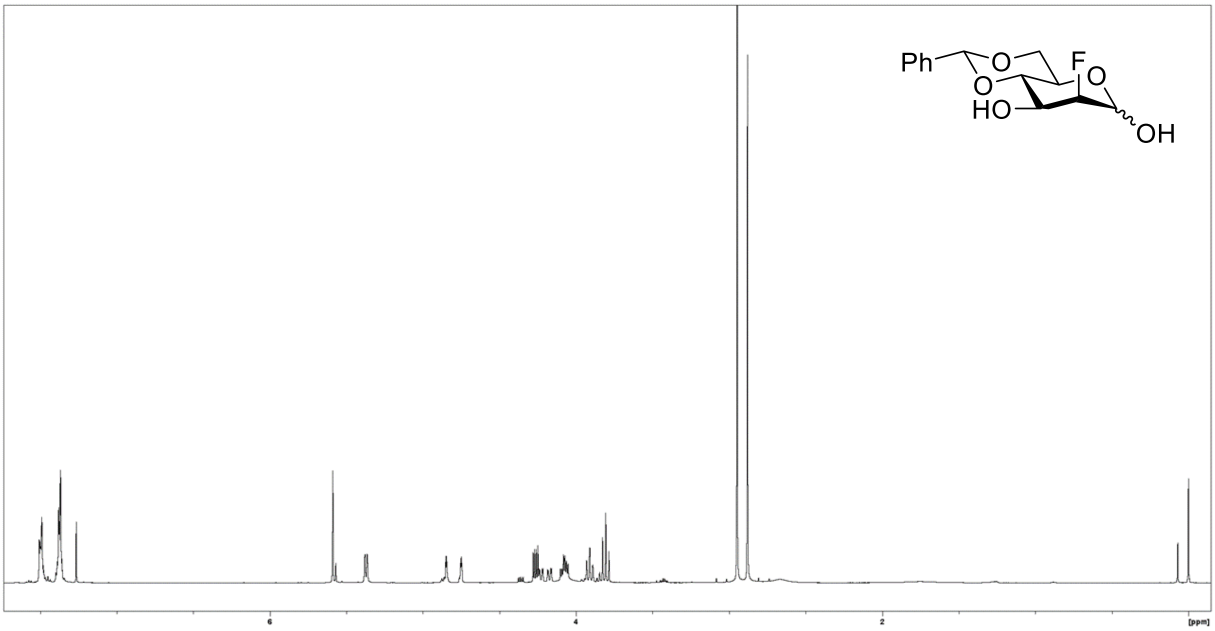

Figure S11. ^{1}H NMR spectrum of **4,6-*O*-benzylidene-2-deoxy-2-fluoro-D-mannopyranose** (499.83 MHz, 25 °C, $CDCl_3$).

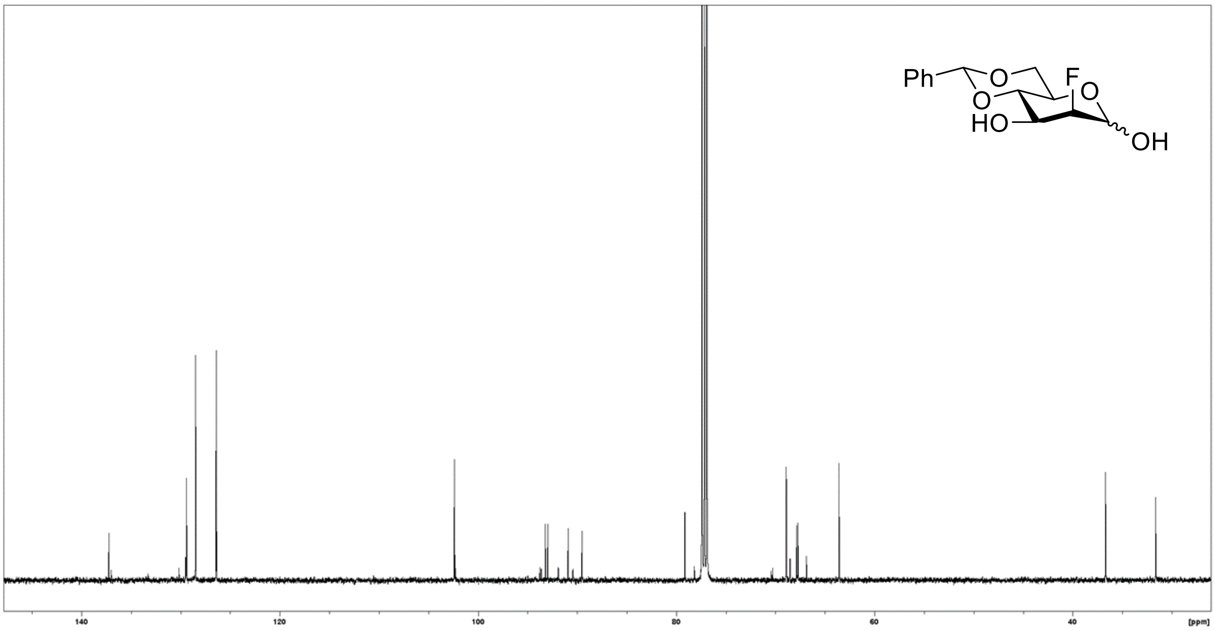

Figure S12. $^{13}C\{^1H\}$ NMR spectrum of **4,6-*O*-benzylidene-2-deoxy-2-fluoro-D-mannopyranose** (125.69 MHz, 25 °C, $CDCl_3$).

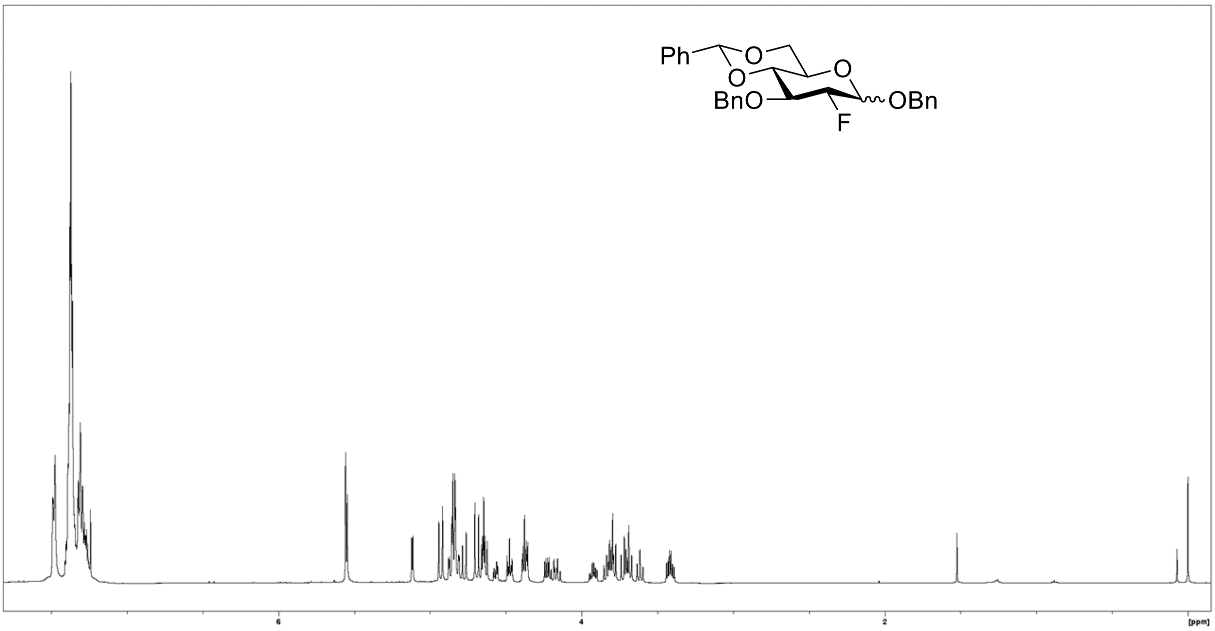

Figure S13. ^{1}H NMR spectrum of **Benzyl 3-*O*-benzyl-4,6-*O*-benzylidene-2-deoxy-2-fluoro-D-glucopyranoside (8)** (499.83 MHz, 25 °C, $CDCl_3$).

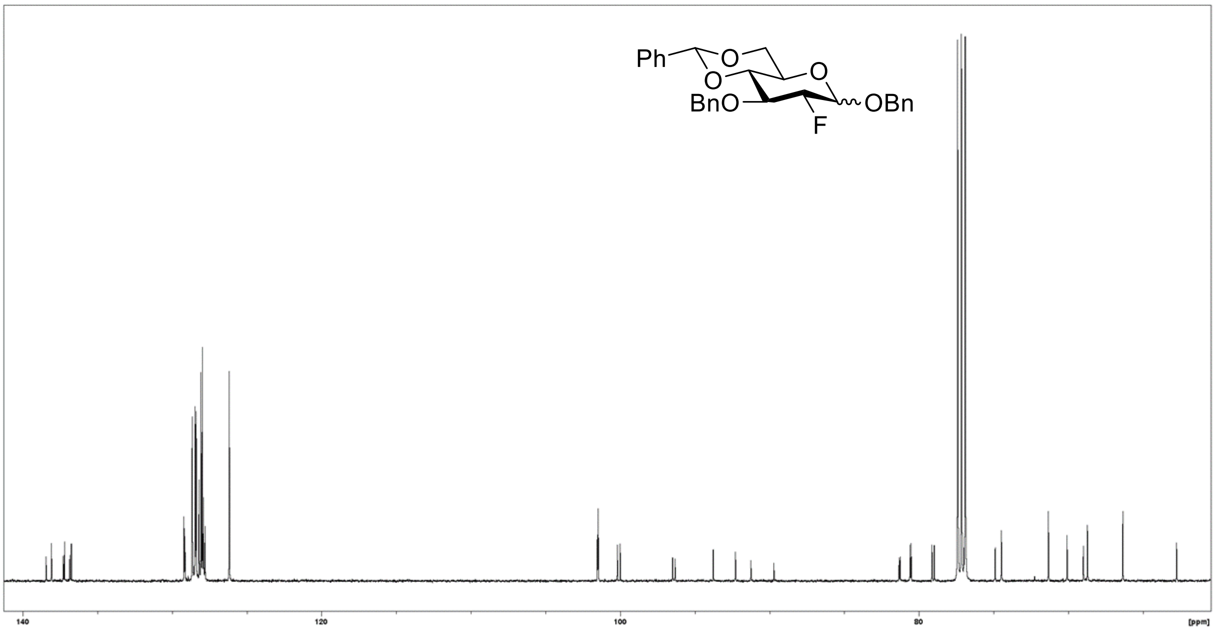

Figure S14. $^{13}C\{^{1}H\}$ NMR spectrum of **Benzyl 3-*O*-benzyl-4,6-*O*-benzylidene-2-deoxy-2-fluoro-D-glucopyranoside (8)** (125.69 MHz, 25 °C, $CDCl_3$).

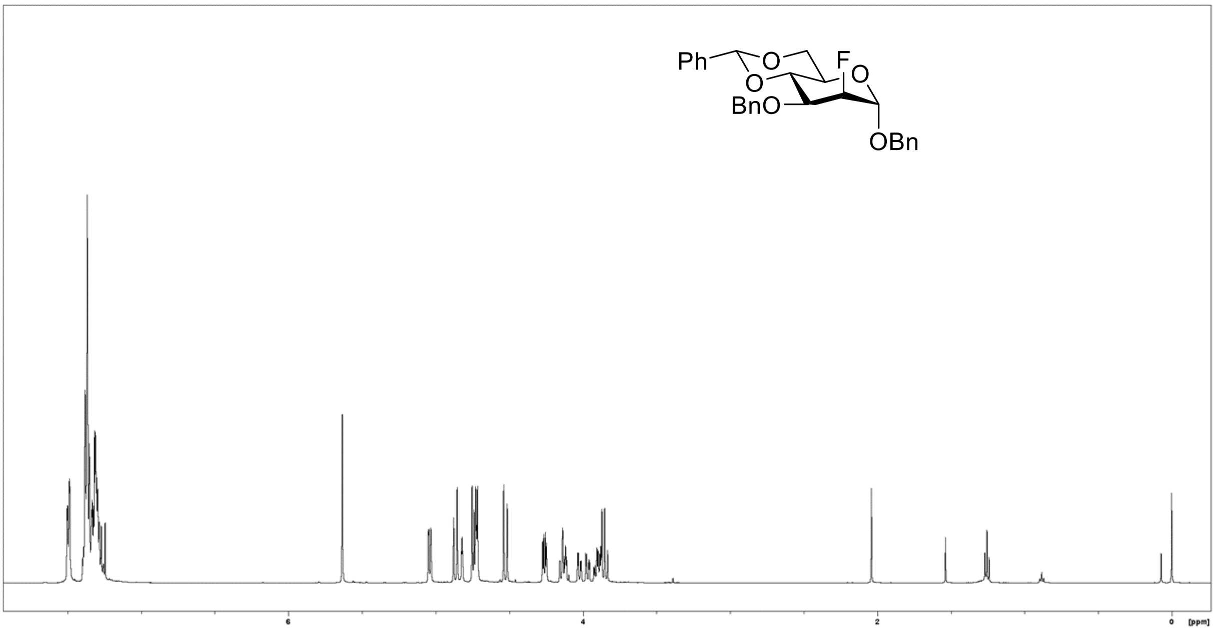

Figure S15. ^{1}H NMR spectrum of **Benzyl 3-*O*-benzyl-4,6-*O*-benzylidene-2-deoxy-2-fluoro-α-D-mannopyranoside (12)** (499.83 MHz, 25 °C, $CDCl_3$).

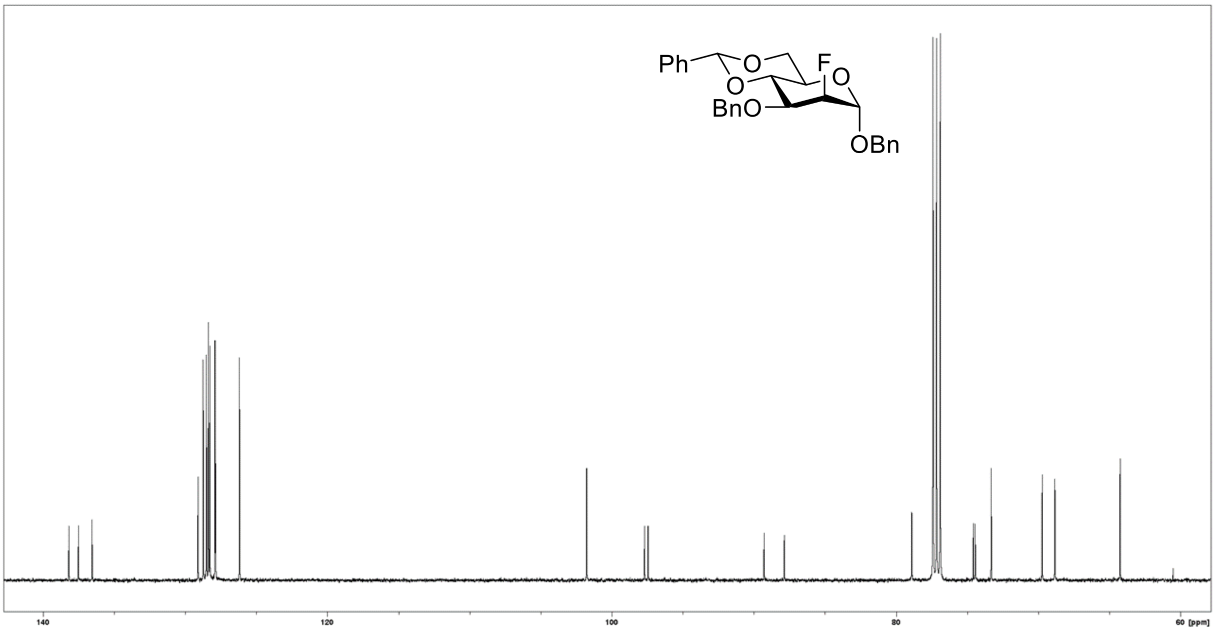

Figure S16. $^{13}C\{^{1}H\}$ NMR spectrum of **Benzyl 3-*O*-benzyl-4,6-*O*-benzylidene-2-deoxy-2-fluoro-α-D-mannopyranoside (12)** (125.69 MHz, 25 °C, $CDCl_3$).

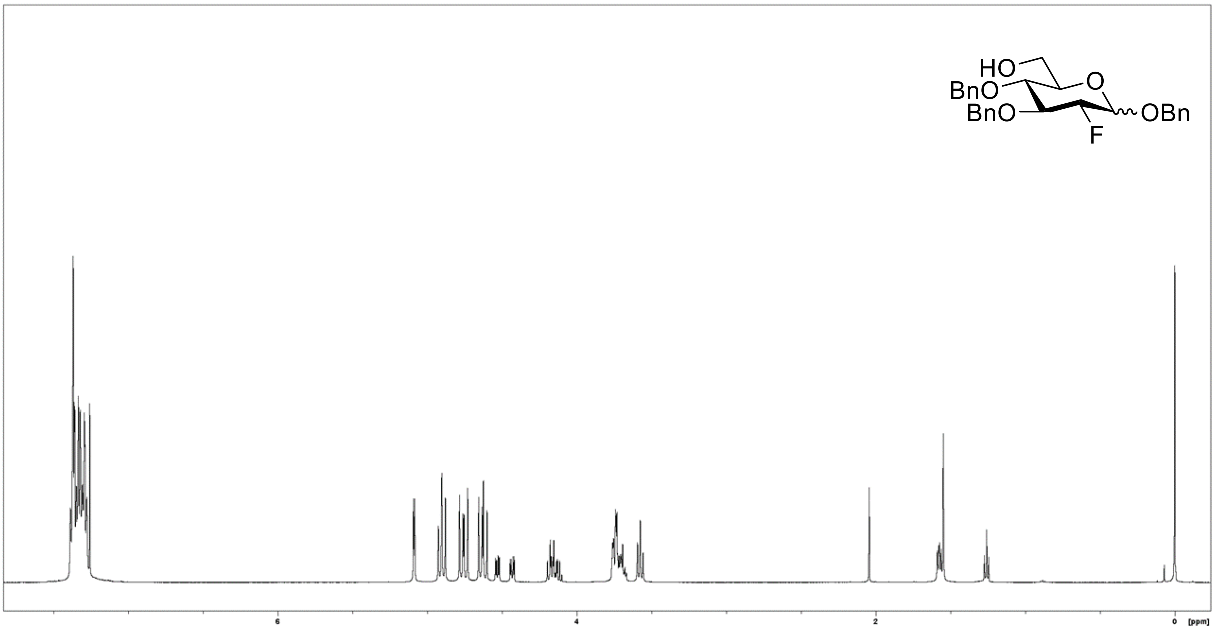

Figure S17. ^{1}H NMR spectrum of **Benzyl 3,4-di-*O*-benzyl-2-deoxy-2-fluoro-D-glucopyranoside (9)** (499.83 MHz, 25 °C, $CDCl_3$).

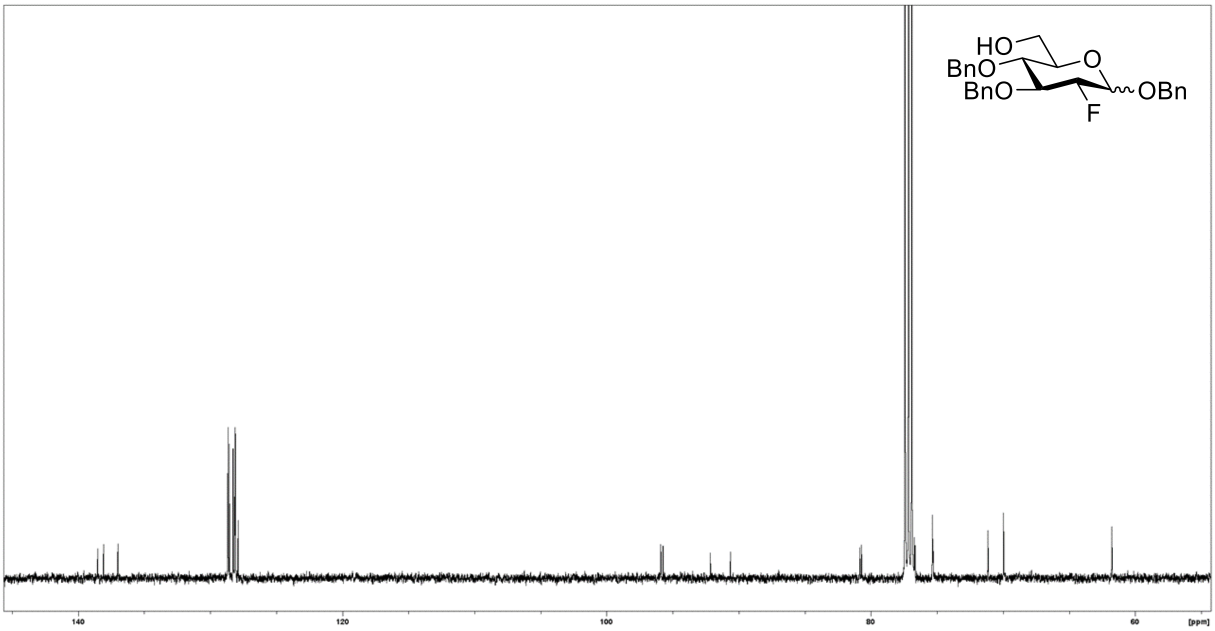

Figure S18. ^{13}C{^{1}H} NMR spectrum of **Benzyl 3,4-di-*O*-benzyl-2-deoxy-2-fluoro-D-glucopyranoside (9)** (125.69 MHz, 25 °C, $CDCl_3$).

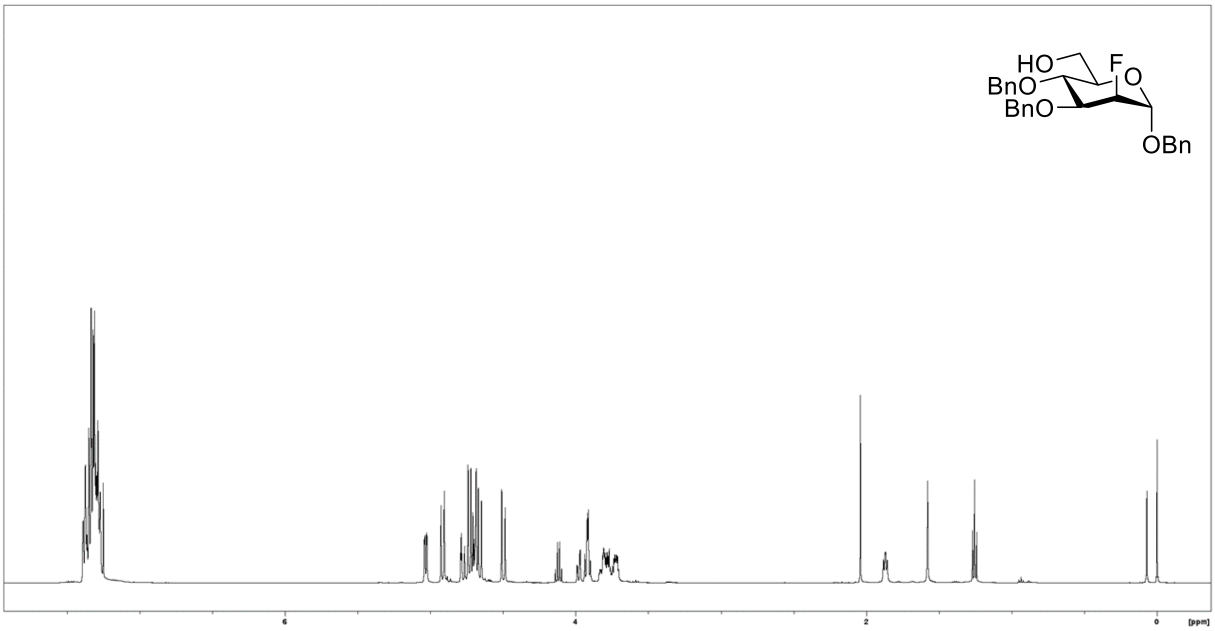

Figure S19. ^{1}H NMR spectrum of **Benzyl 3,4-di-*O*-benzyl-2-deoxy-2-fluoro-α-D-mannopyranoside (13)** (499.83 MHz, 25 °C, $CDCl_3$).

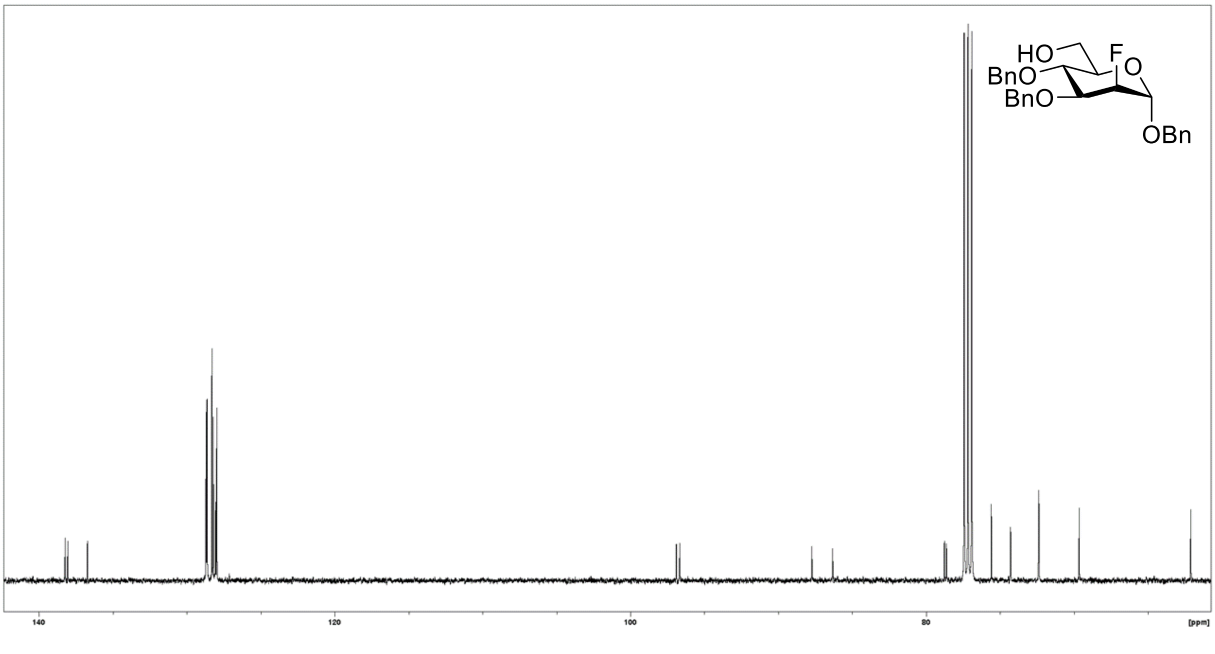

Figure S20. $^{13}C\{^{1}H\}$ NMR spectrum of **Benzyl 3,4-di-*O*-benzyl-2-deoxy-2-fluoro-α-D-mannopyranoside (13)** (125.69 MHz, 25 °C, $CDCl_3$).

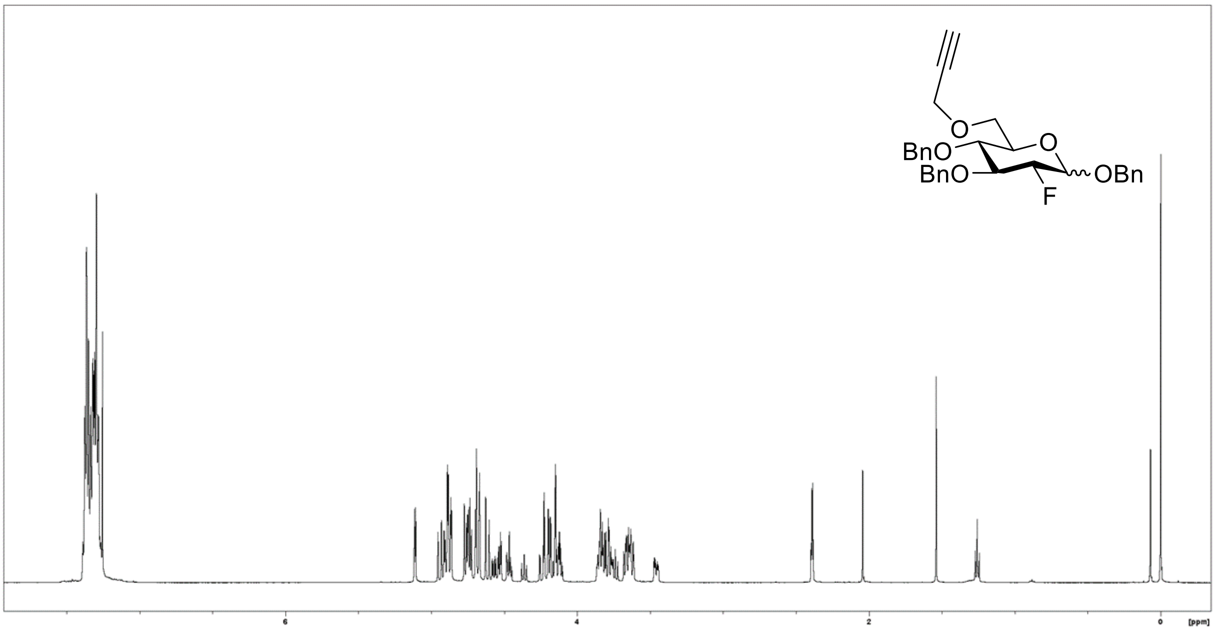

Figure S21. ^{1}H NMR spectrum of **Benzyl 3,4-di-*O*-benzyl-2-deoxy-2-fluoro-6-*O*-propargyl-D-glucopyranoside** (499.83 MHz, 25 °C, $CDCl_3$).

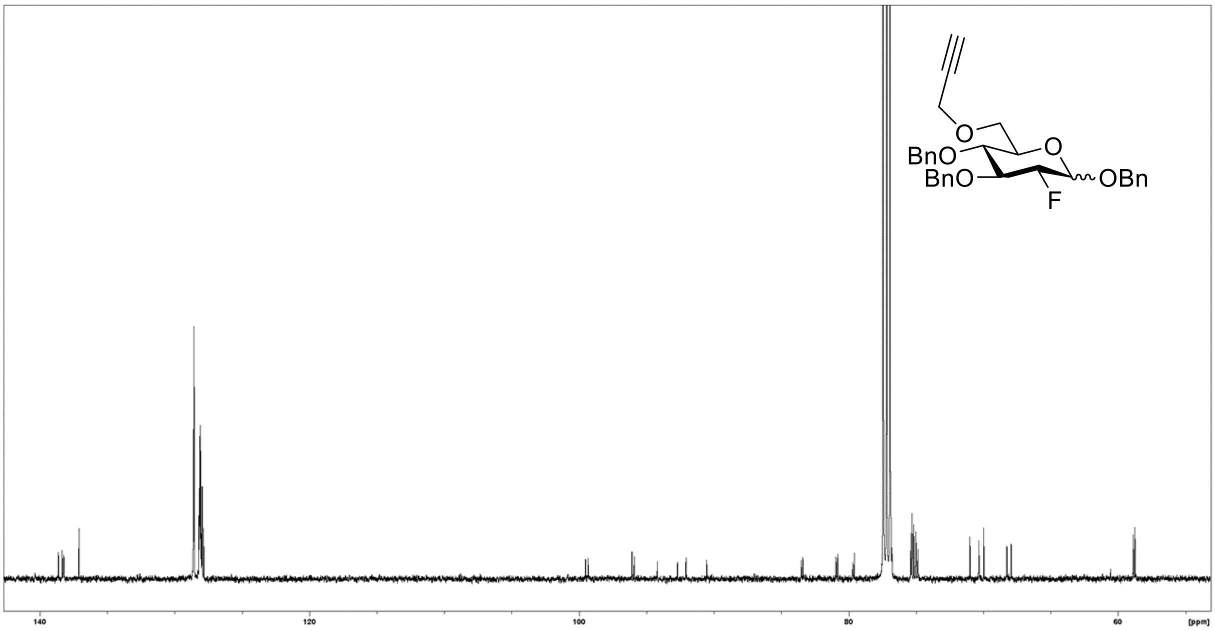

Figure S22. $^{13}C\{^{1}H\}$ NMR spectrum of **Benzyl 3,4-di-*O*-benzyl-2-deoxy-2-fluoro-6-*O*-propargyl-D-glucopyranoside** (125.69 MHz, 25 °C, $CDCl_3$).

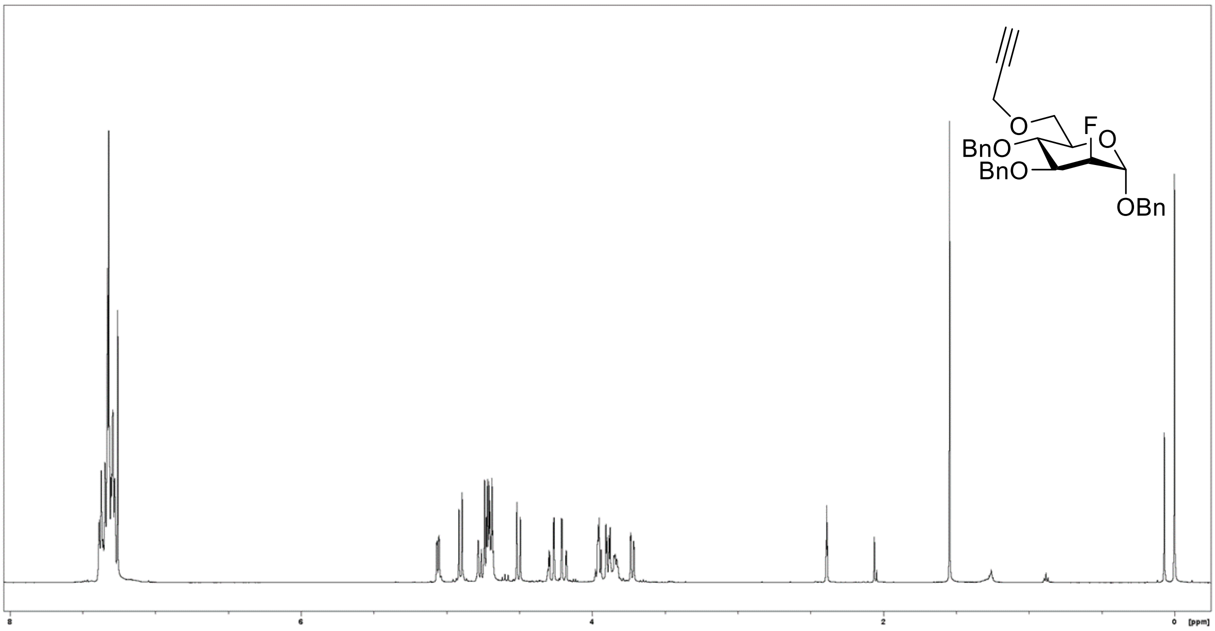

Figure S23. ^{1}H NMR spectrum of **Benzyl 3,4-di-*O*-benzyl-2-deoxy-2-fluoro-6-*O*-propargyl-α-D-mannopyranoside** (499.83 MHz, 25 °C, $CDCl_3$).

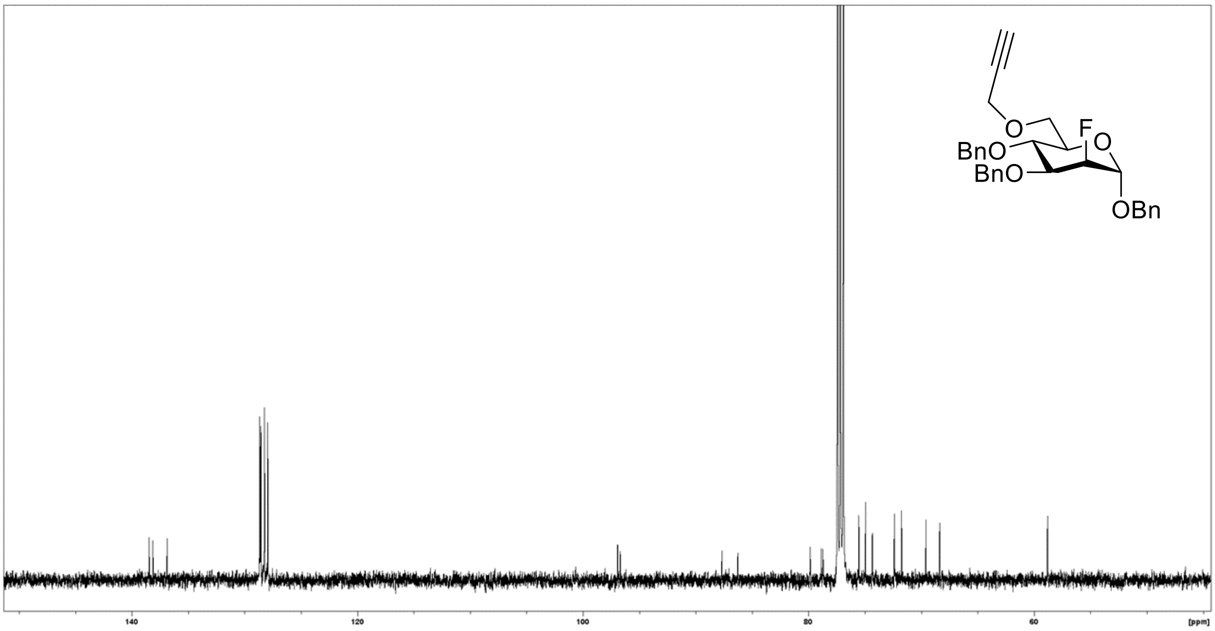

Figure S24. $^{13}C\{^1H\}$ NMR spectrum of **Benzyl 3,4-di-*O*-benzyl-2-deoxy-2-fluoro-6-*O*-propargyl-α-D-mannopyranoside** (125.69 MHz, 25 °C, $CDCl_3$).

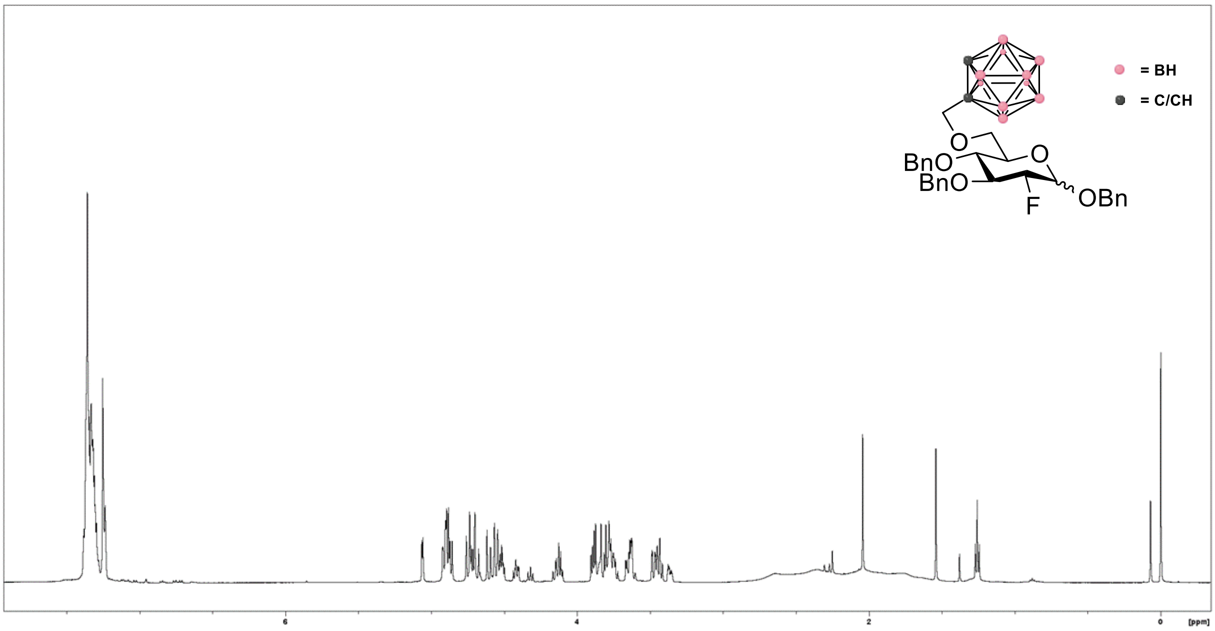

Figure S25. ^{1}H NMR spectrum of **Benzyl 3,4-di-*O*-benzyl-2-deoxy-2-fluoro-6-*O*-carboranylmethyl-D-glucopyranoside** (499.83 MHz, 25 °C, $CDCl_3$).

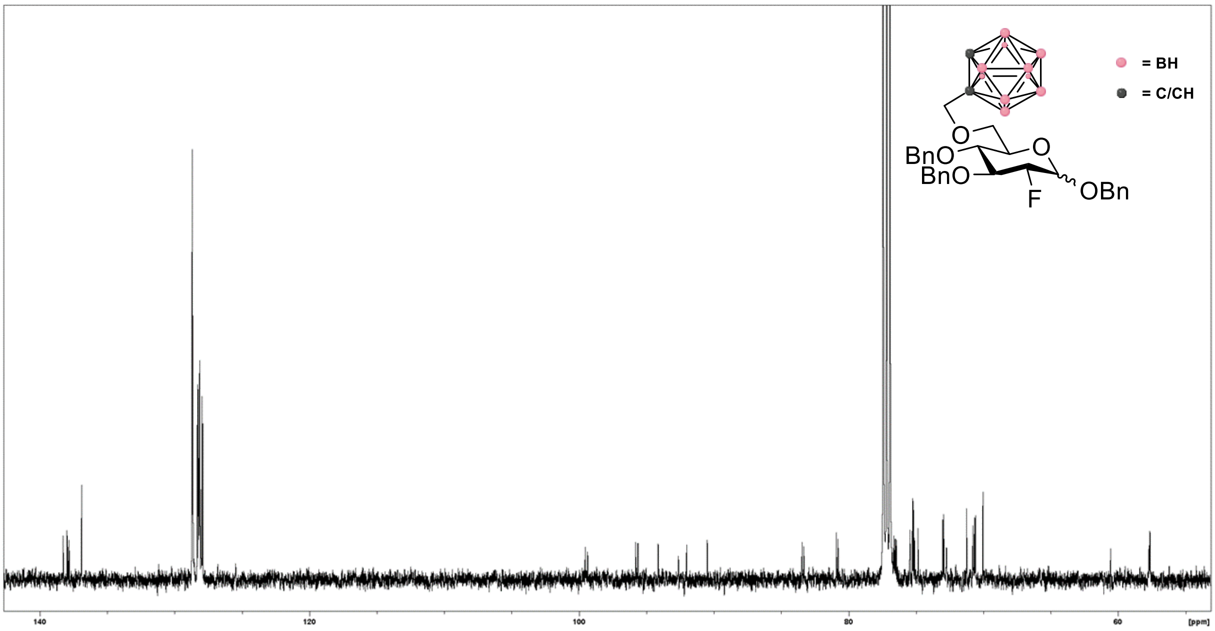

Figure S26. $^{13}C\{^{1}H\}$ NMR spectrum of **Benzyl 3,4-di-*O*-benzyl-2-deoxy-2-fluoro-6-*O*-carboranylmethyl-D-glucopyranoside** (125.69 MHz, 25 °C, $CDCl_3$).

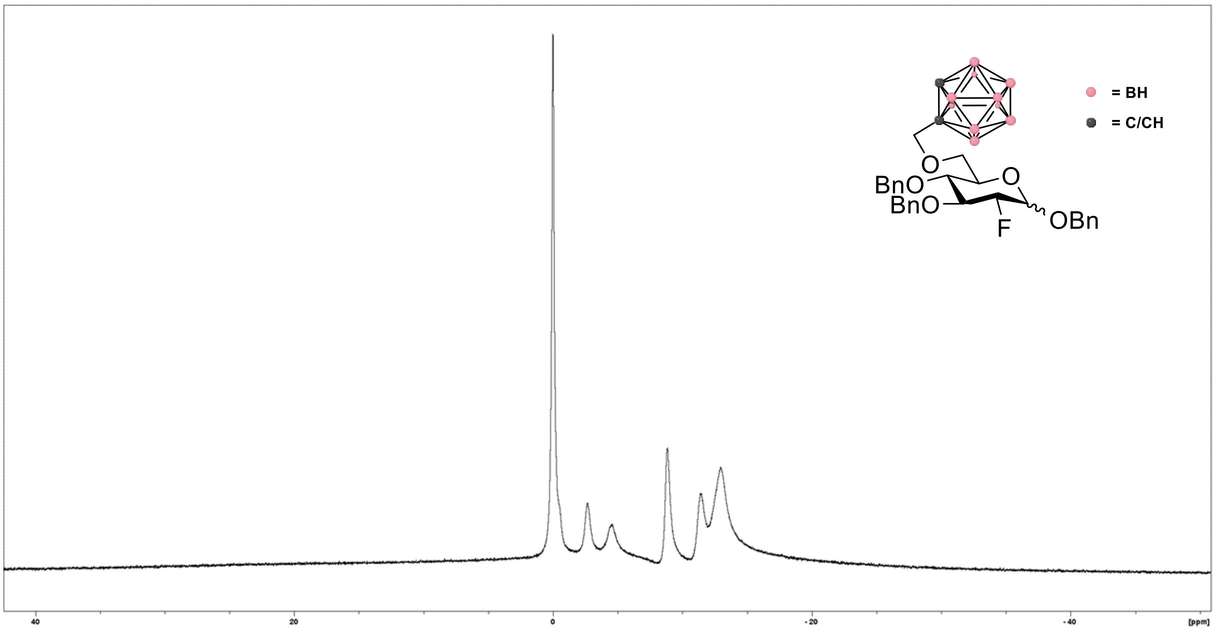

Figure S27. $^{11}B\{^{1}H\}$ NMR spectrum of **Benzyl 3,4-di-*O*-benzyl-2-deoxy-2-fluoro-6-*O*-carboranylmethyl-D-glucopyranoside** (160.36 MHz, 25 °C, $CDCl_3$).

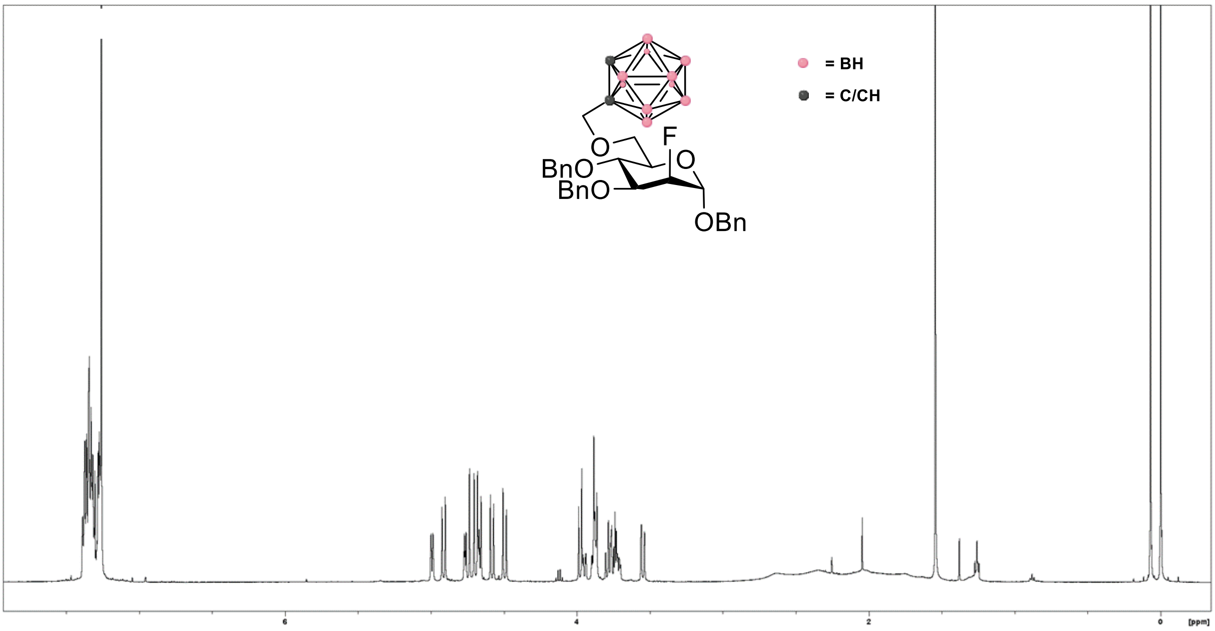

Figure S28. ^{1}H NMR spectrum of **Benzyl 3,4-di-*O*-benzyl-2-deoxy-2-fluoro-6-*O*-carboranylmethyl-α-D-mannopyranoside** (499.83 MHz, 25 °C, $CDCl_3$).

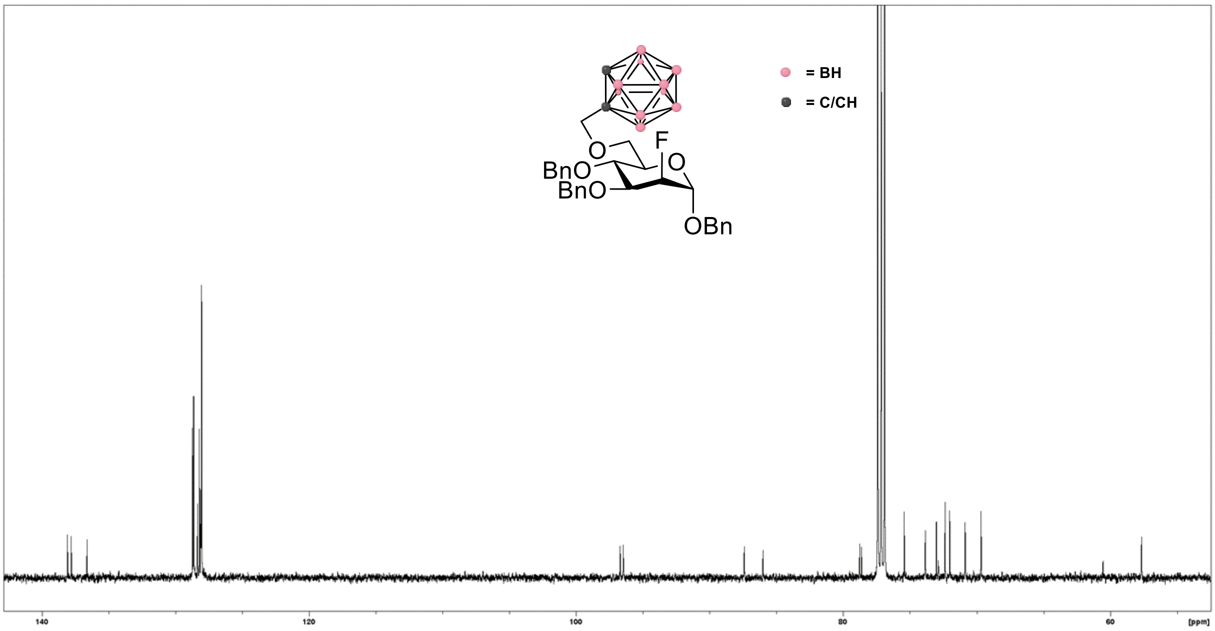

Figure S29. $^{13}C\{^{1}H\}$ NMR spectrum of **Benzyl 3,4-di-*O*-benzyl-2-deoxy-2-fluoro-6-*O*-carboranylmethyl-α-D-mannopyranoside** (125.69 MHz, 25 °C, $CDCl_3$).

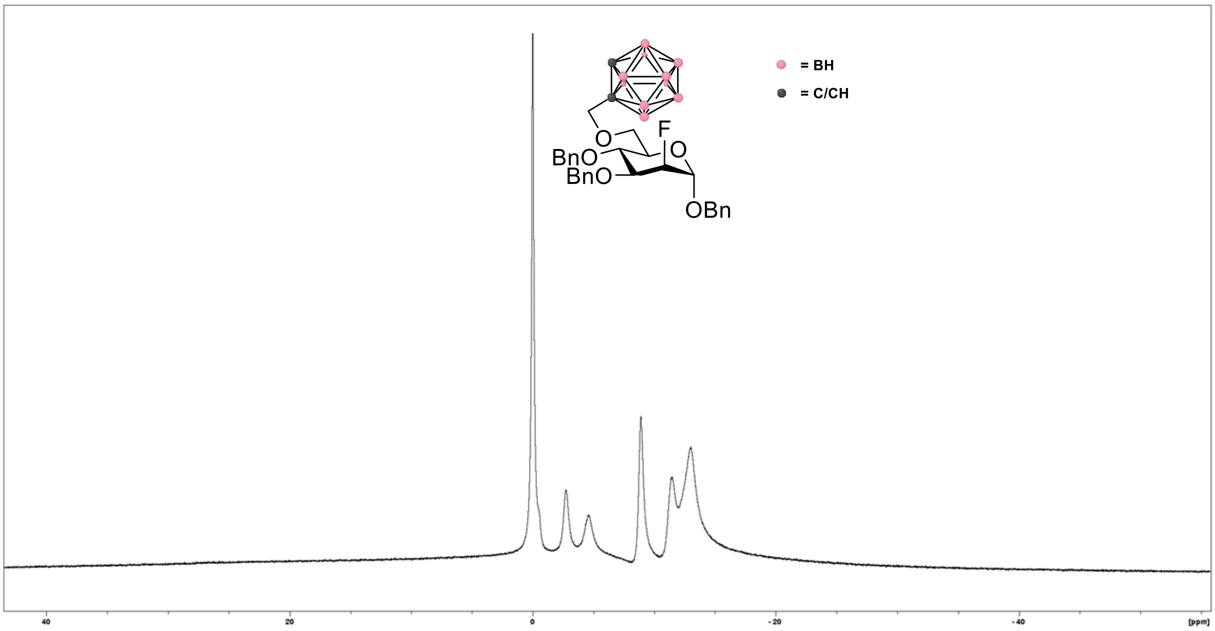

Figure S30. $^{11}B\{^{1}H\}$ NMR spectrum of **Benzyl 3,4-di-*O*-benzyl-2-deoxy-2-fluoro-6-*O*-carboranylmethyl-α-D-mannopyranoside** (160.36 MHz, 25 °C, $CDCl_3$).

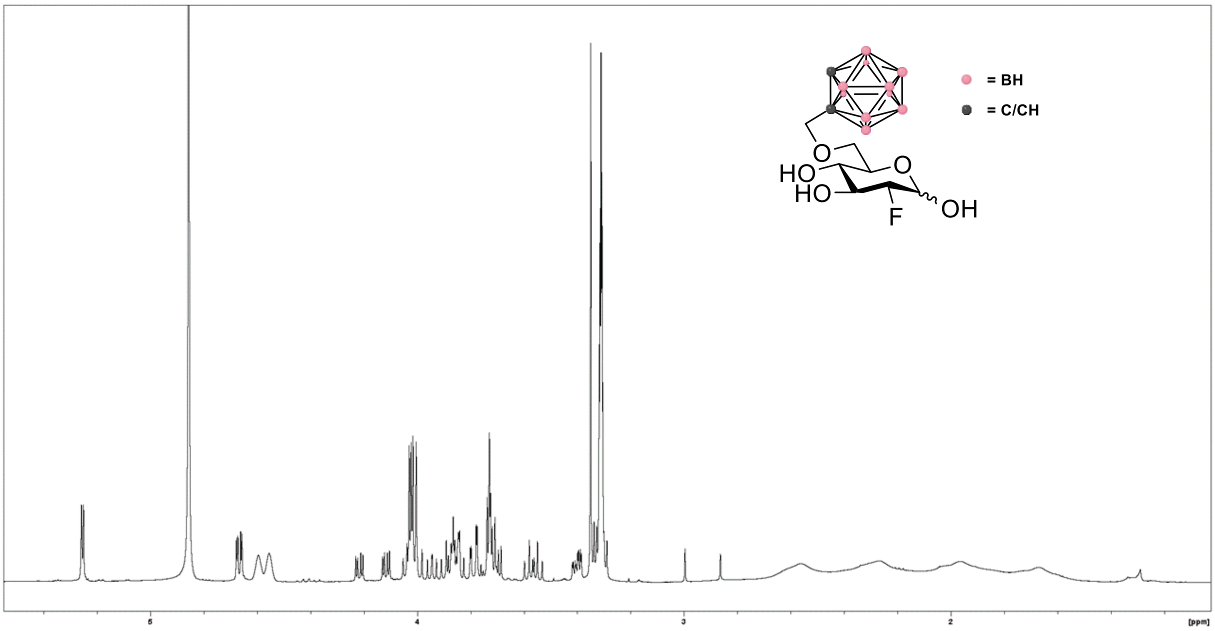

Figure S31. ^{1}H NMR spectrum of **2-deoxy-2-fluoro-6-*O*-carboranylmethyl-D-glucopyranose (1)** (499.83 MHz, 25 °C, CD_3OD).

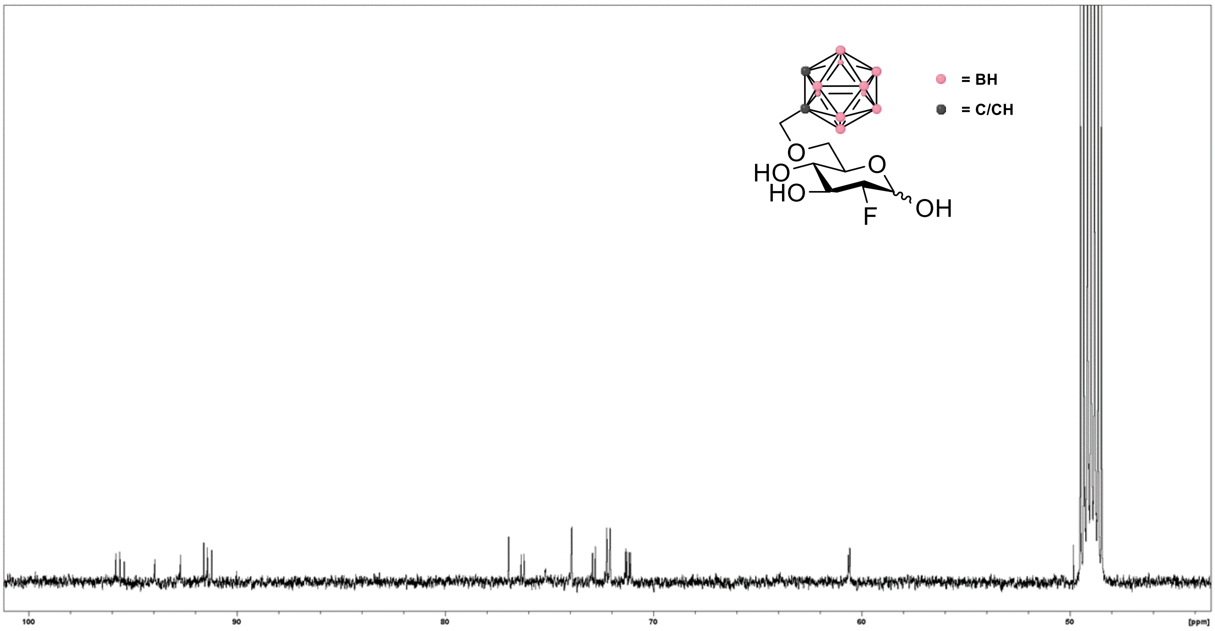

Figure S32. $^{13}C\{^{1}H\}$ NMR spectrum of **2-deoxy-2-fluoro-6-*O*-carboranylmethyl-D-glucopyranose (1)** (125.69 MHz, 25 °C, CD_3OD).

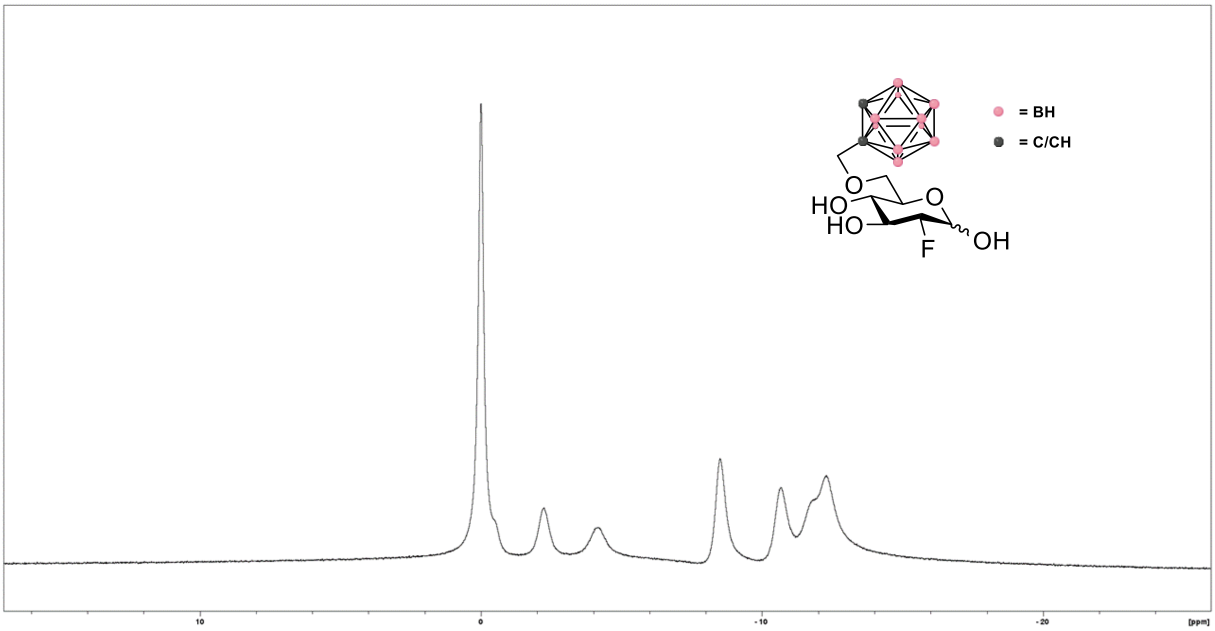

Figure S33. $^{11}B\{^{1}H\}$ NMR spectrum of **2-deoxy-2-fluoro-6-*O*-carboranylmethyl-D-glucopyranose (1)** (160.36 MHz, 25 °C, CD_3OD).

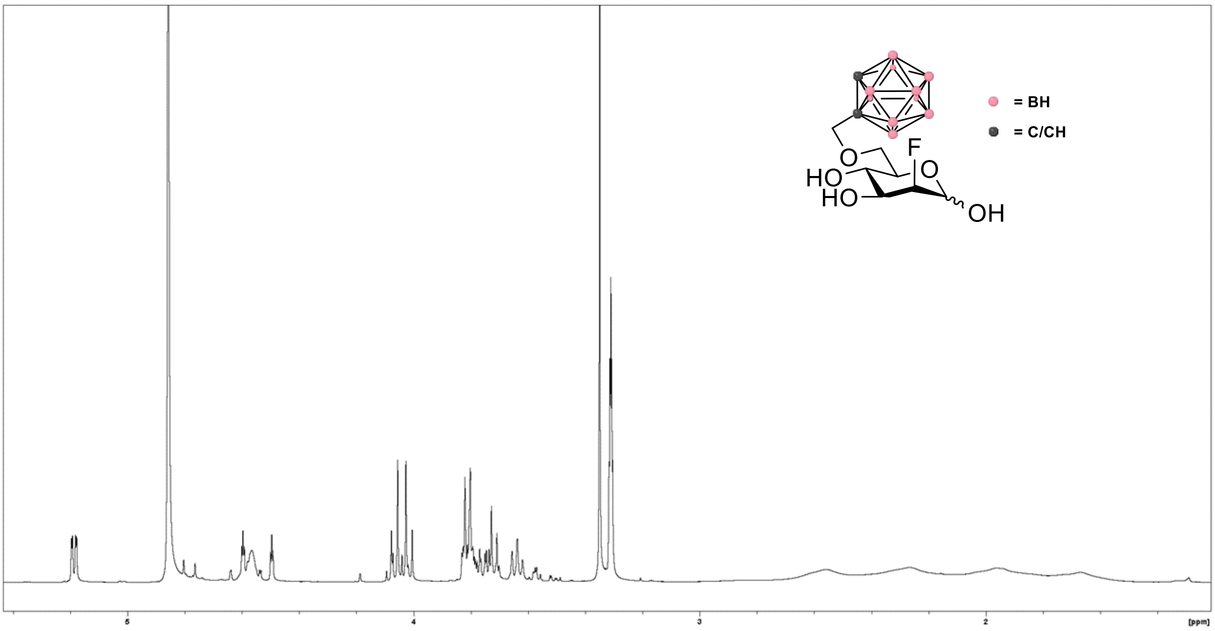

Figure S34. ^{1}H NMR spectrum of **2-deoxy-2-fluoro-6-*O*-carboranylmethyl-D-mannopyranose (3)** (499.83 MHz, 25 °C, CD_3OD).

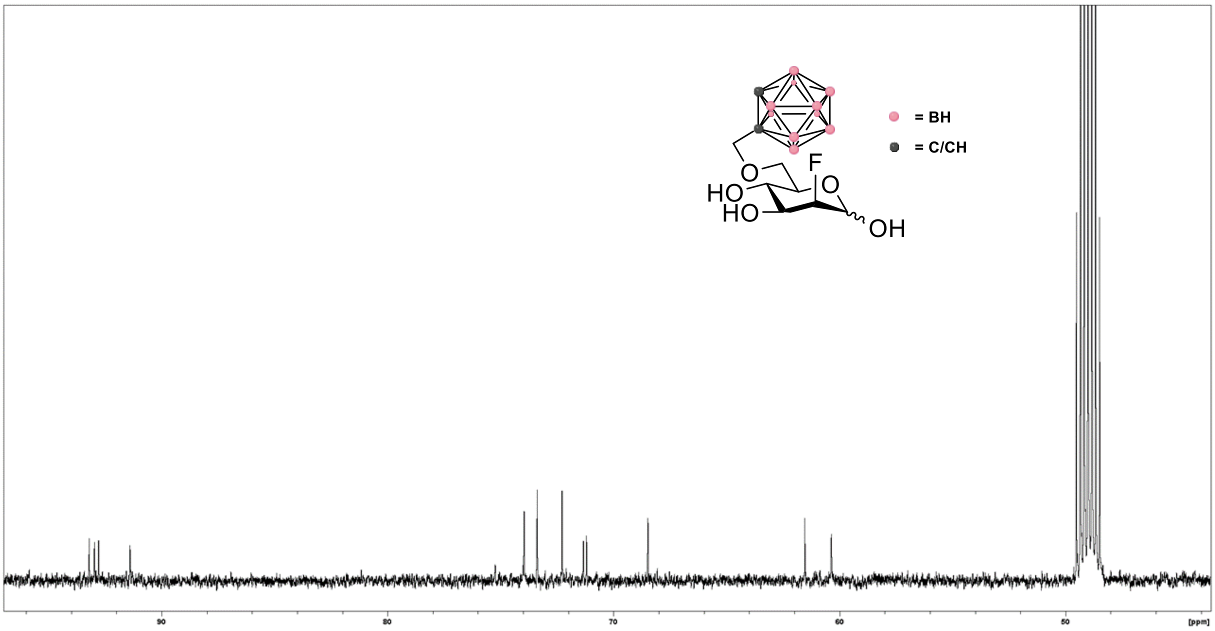

Figure S35. $^{13}C\{^{1}H\}$ NMR spectrum of **2-deoxy-2-fluoro-6-*O*-carboranylmethyl-D-mannopyranose (3)** (125.69 MHz, 25 °C, CD_3OD).

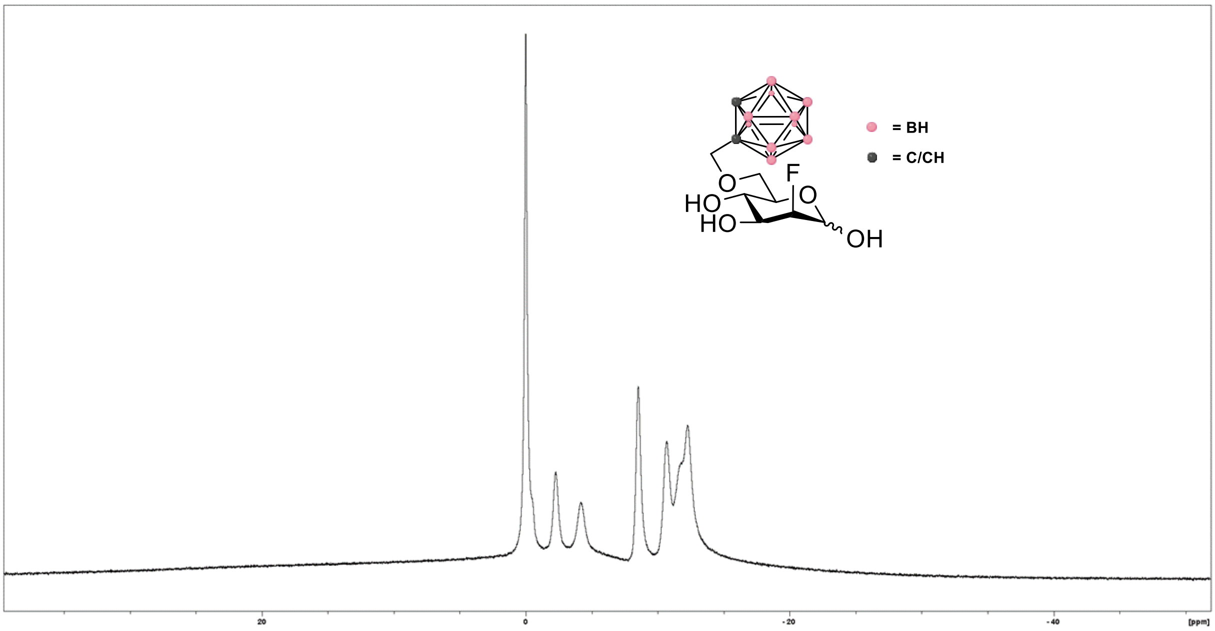

Figure S36. $^{11}B\{^{1}H\}$ NMR spectrum of **2-deoxy-2-fluoro-6-*O*-carboranylmethyl-D-mannopyranose (3)** (160.36 MHz, 25 °C, CD_3OD).

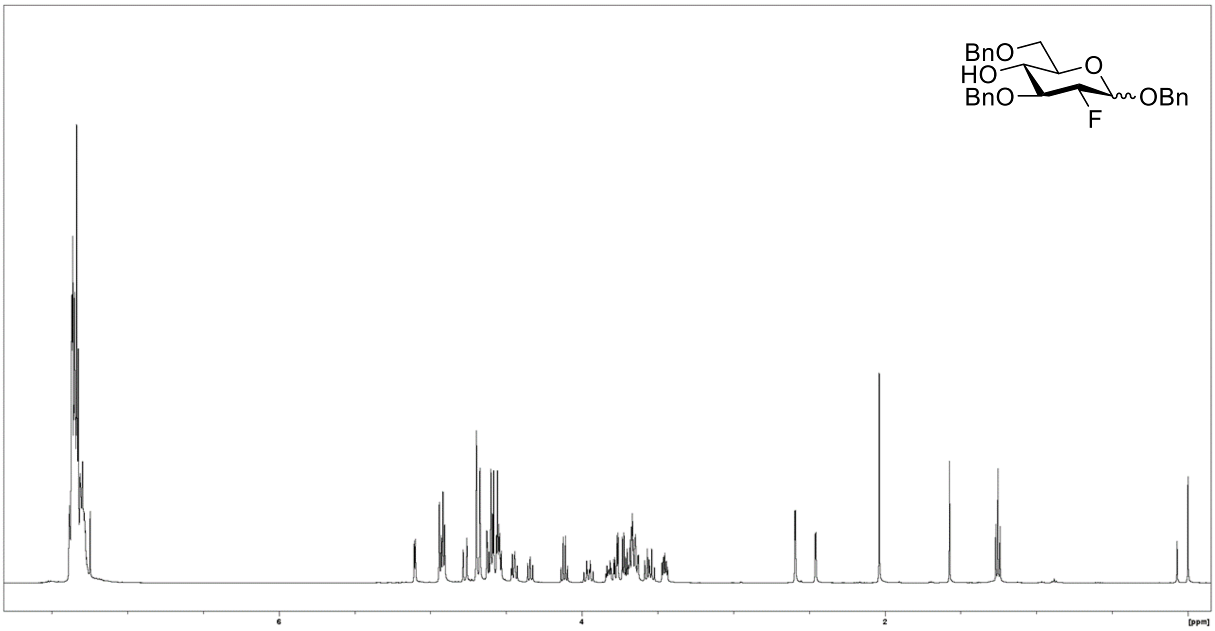

Figure S37. ^{1}H NMR spectrum of **Benzyl 3,6-di-*O*-benzyl-2-deoxy-2-fluoro-D-glucopyranoside (10)** (499.83 MHz, 25 °C, $CDCl_3$).

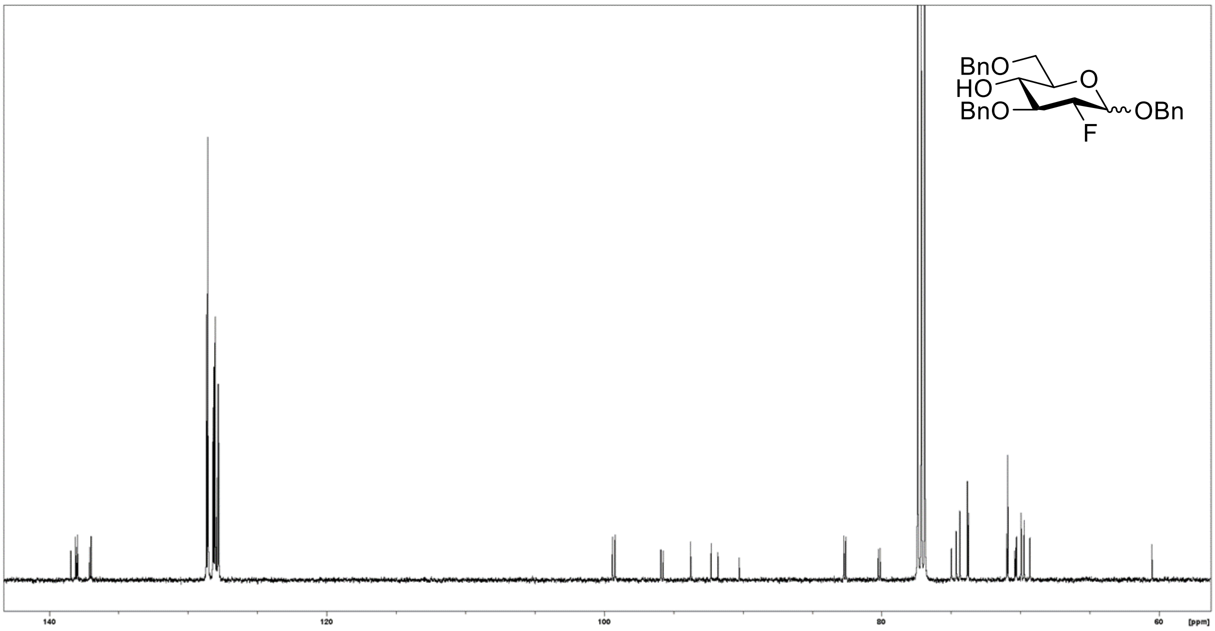

Figure S38. $^{13}C\{^{1}H\}$ NMR spectrum of **Benzyl 3,6-di-*O*-benzyl-2-deoxy-2-fluoro-D-glucopyranoside (10)** (125.69 MHz, 25 °C, $CDCl_3$).

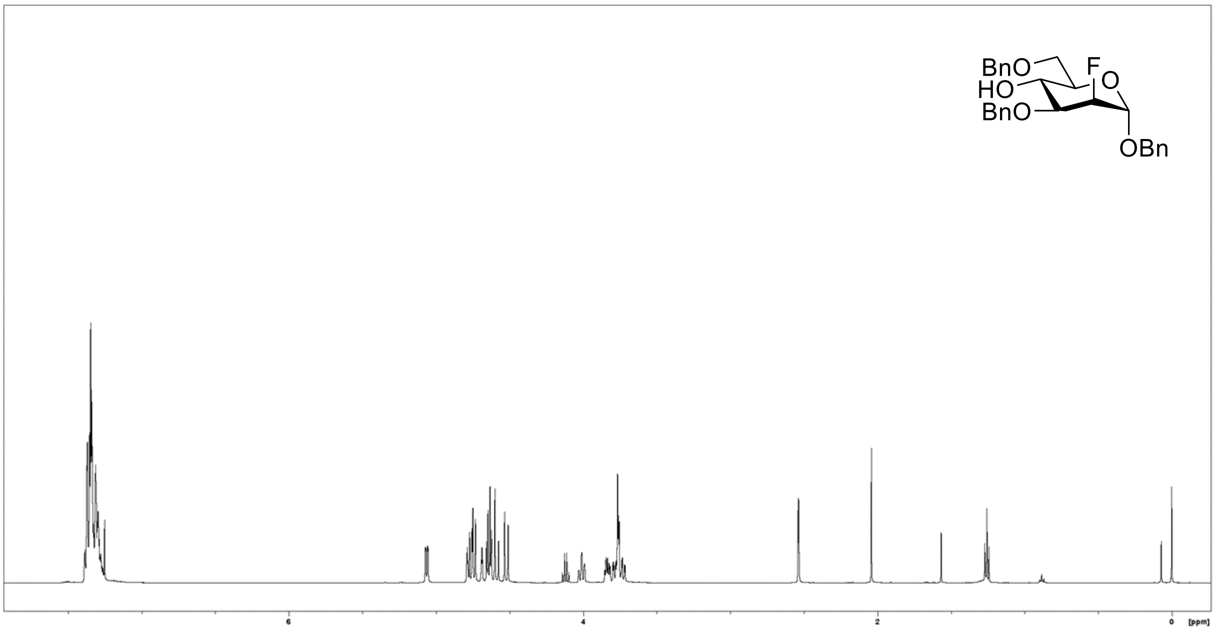

Figure S39. ^{1}H NMR spectrum of **Benzyl 3,6-di-*O*-benzyl-2-deoxy-2-fluoro-α-D-mannopyranoside (14)** (499.83 MHz, 25 °C, $CDCl_3$).

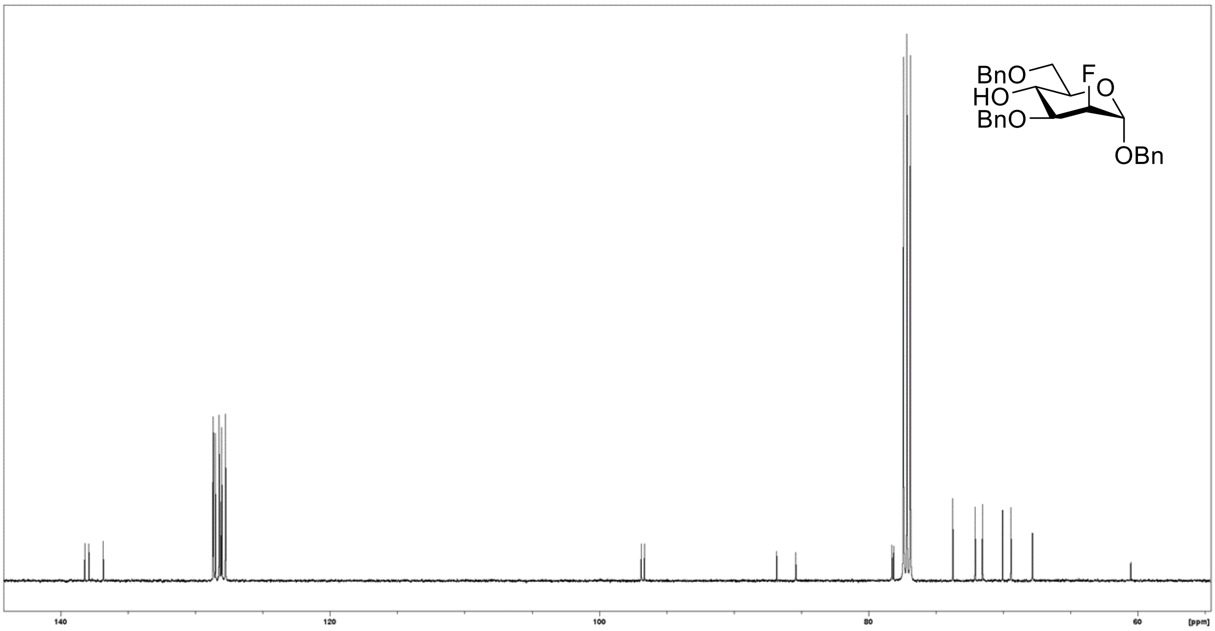

Figure S40. $^{13}C\{^{1}H\}$ NMR spectrum of **Benzyl 3,6-di-*O*-benzyl-2-deoxy-2-fluoro-α-D-mannopyranoside (14)** (125.69 MHz, 25 °C, $CDCl_3$).

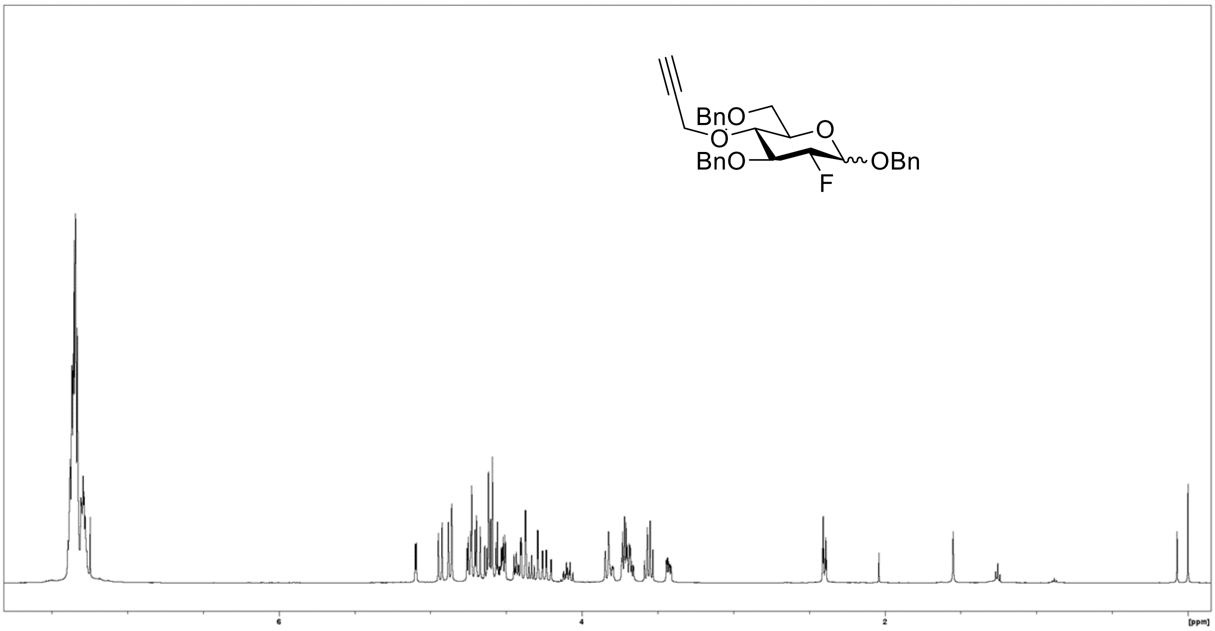

Figure S41. ^{1}H NMR spectrum of **Benzyl 3,6-di-*O*-benzyl-2-deoxy-2-fluoro-4-*O*-propargyl-D-glucopyranoside** (499.83 MHz, 25 °C, $CDCl_3$).

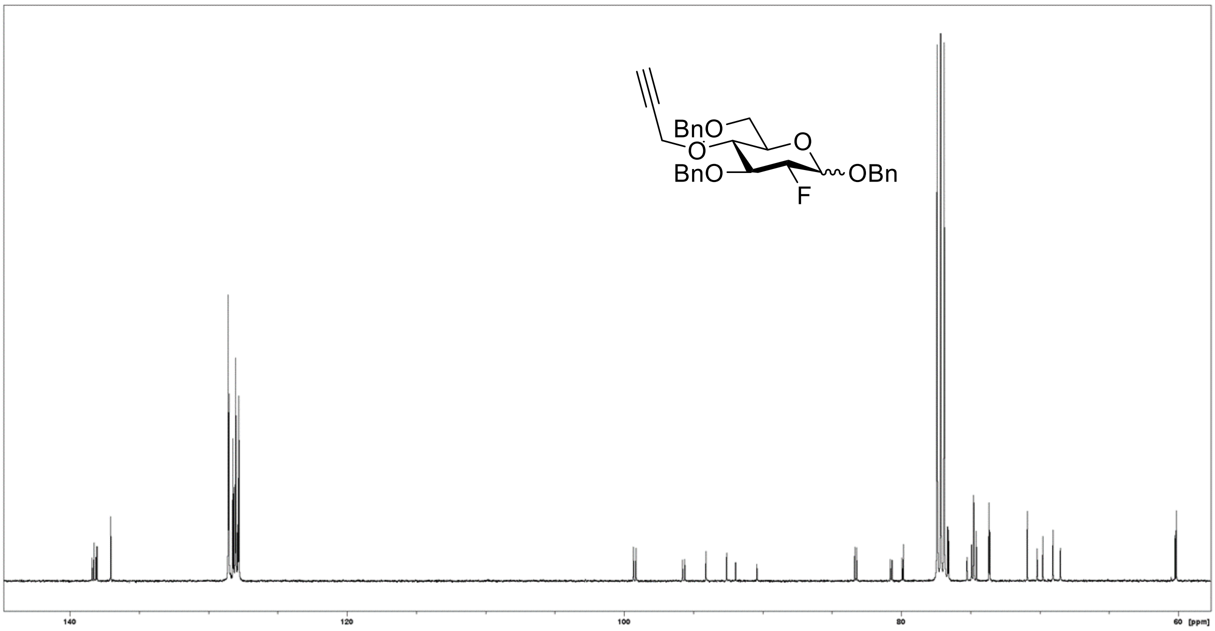

Figure S42. $^{13}C\{^1H\}$ NMR spectrum of **Benzyl 3,6-di-*O*-benzyl-2-deoxy-2-fluoro-4-*O*-propargyl-D-glucopyranoside** (125.69 MHz, 25 °C, $CDCl_3$).

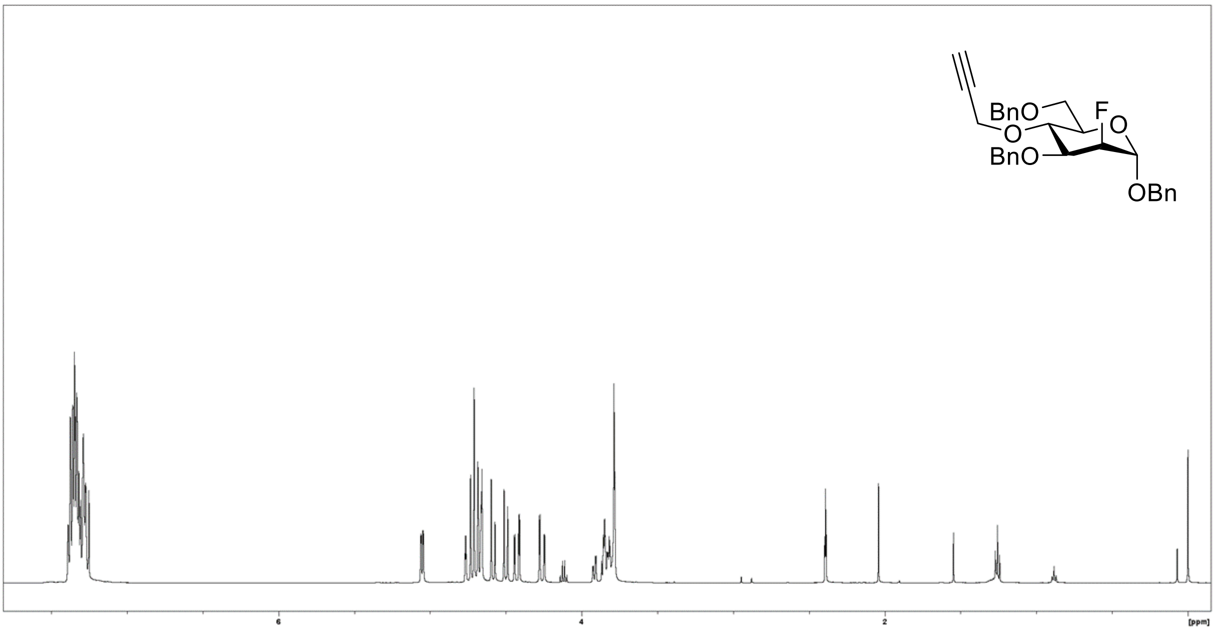

Figure S43. ^{1}H NMR spectrum of **Benzyl 3,6-di-*O*-benzyl-2-deoxy-2-fluoro-4-*O*-propargyl-α-D-mannopyranoside** (499.83 MHz, 25 °C, $CDCl_3$).

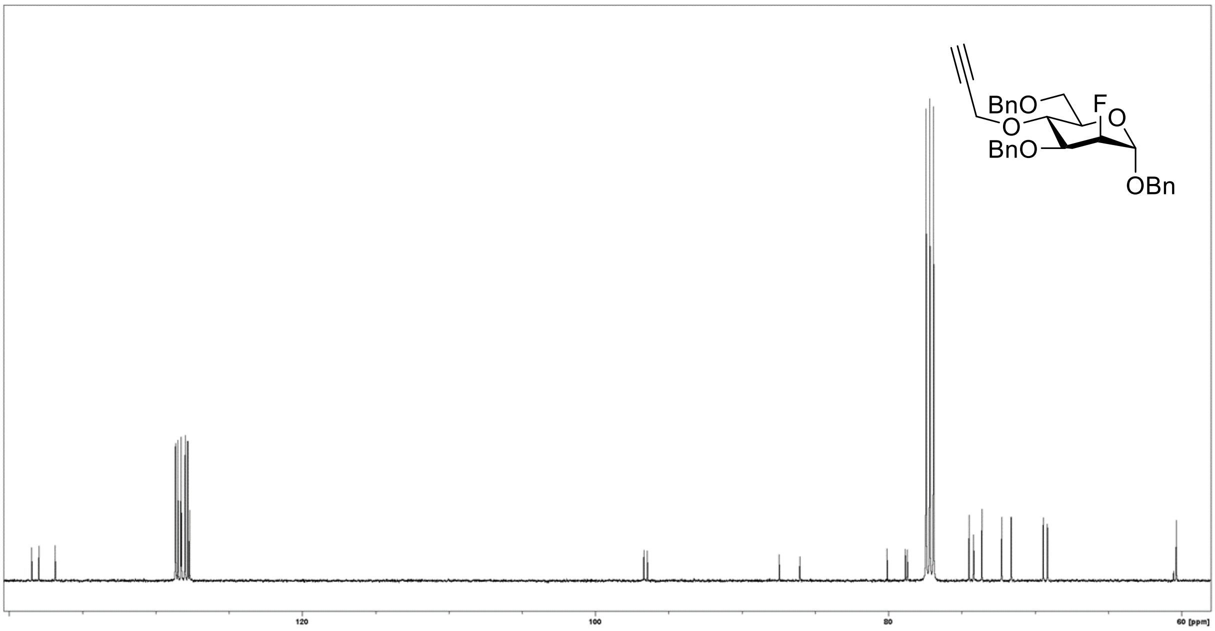

Figure S44. ^{13}C{^{1}H} NMR spectrum of **Benzyl 3,6-di-*O*-benzyl-2-deoxy-2-fluoro-4-*O*-propargyl-α-D-mannopyranoside** (125.69 MHz, 25 °C, $CDCl_3$).

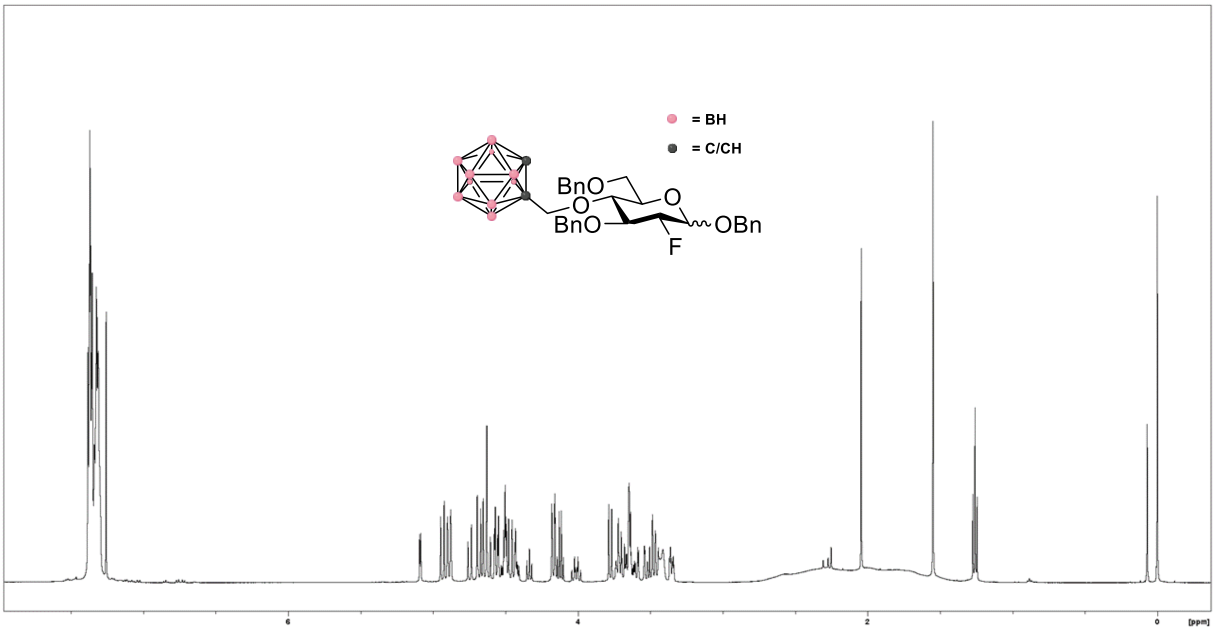

Figure S45. ^{1}H NMR spectrum of **Benzyl 3,6-di-*O*-benzyl-2-deoxy-2-fluoro-4-*O*-carboranylmethyl-D-glucopyranoside** (499.83 MHz, 25 °C, $CDCl_3$).

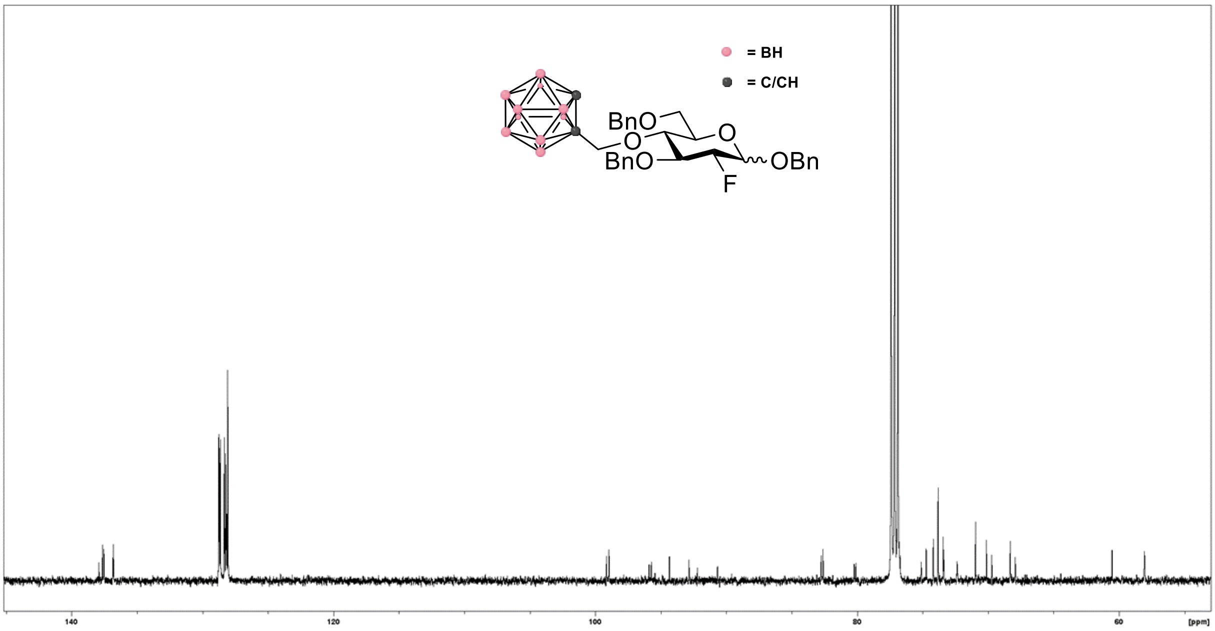

Figure S46. $^{13}C\{^{1}H\}$ NMR spectrum of **Benzyl 3,6-di-*O*-benzyl-2-deoxy-2-fluoro-4-*O*-carboranylmethyl-D-glucopyranoside** (125.69 MHz, 25 °C, $CDCl_3$).

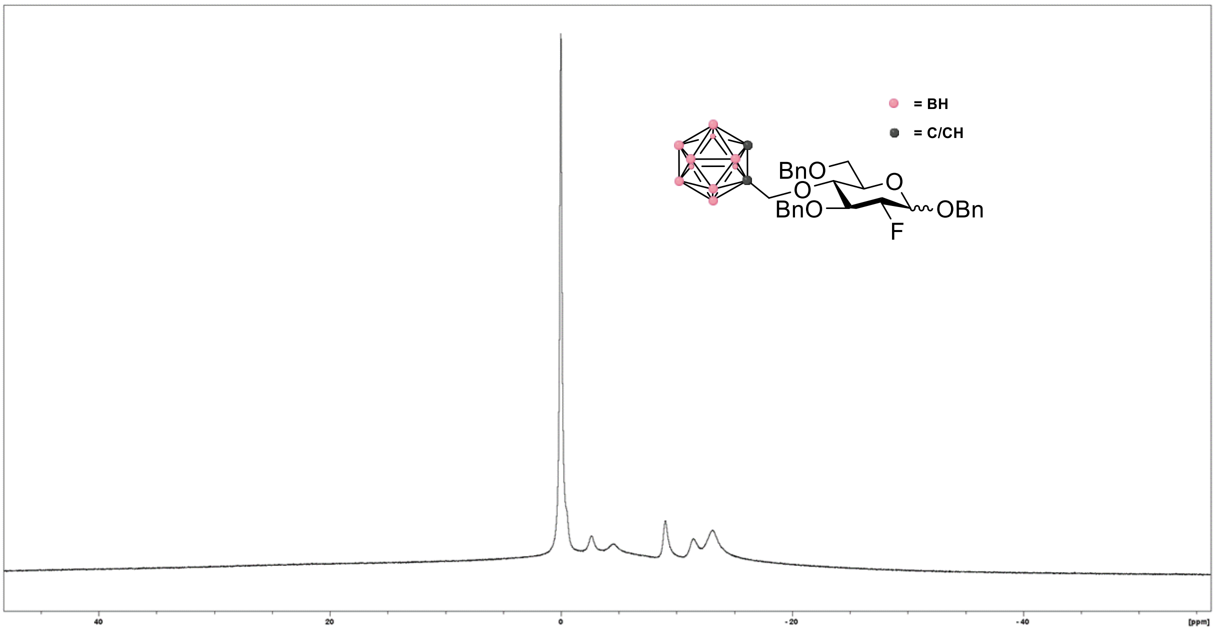

Figure S47. $^{11}B\{^{1}H\}$ NMR spectrum of **Benzyl 3,6-di-*O*-benzyl-2-deoxy-2-fluoro-4-*O*-carboranylmethyl-D-glucopyranoside** (160.36 MHz, 25 °C, $CDCl_3$).

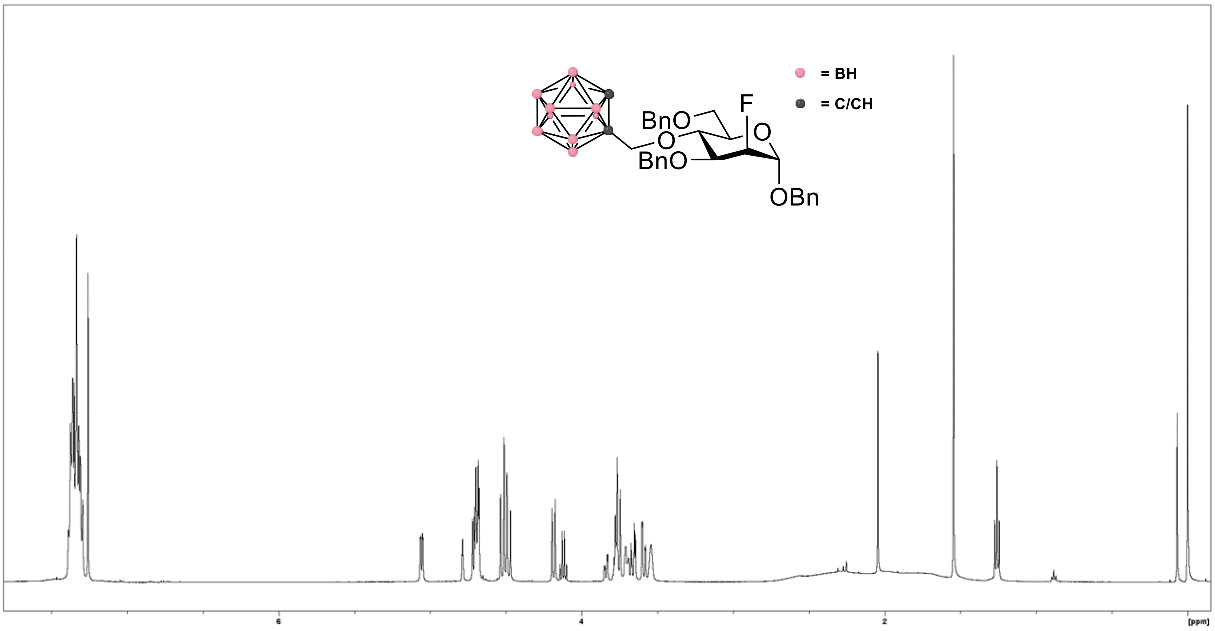

Figure S48. ^{1}H NMR spectrum of **Benzyl 3,6-di-*O*-benzyl-2-deoxy-2-fluoro-4-*O*-carboranylmethyl-α-D-mannopyranoside** (499.83 MHz, 25 °C, $CDCl_3$).

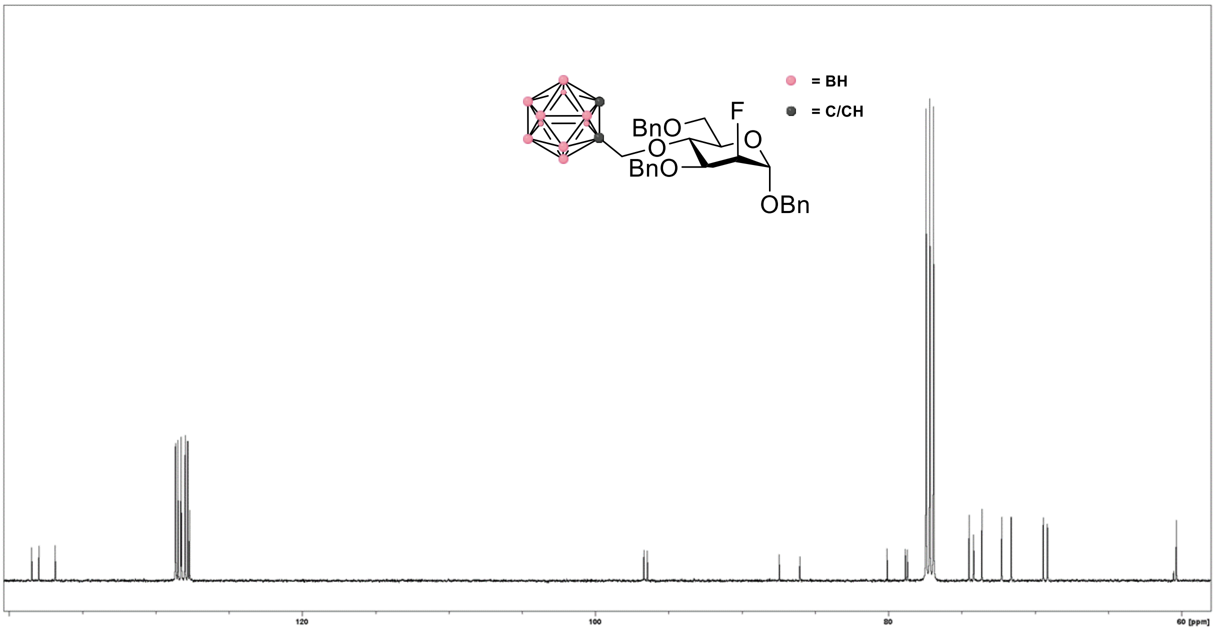

Figure S49. $^{13}C\{^{1}H\}$ NMR spectrum of **Benzyl 3,6-di-*O*-benzyl-2-deoxy-2-fluoro-4-*O*-carboranylmethyl-α-D-mannopyranoside** (125.69 MHz, 25 °C, $CDCl_3$).

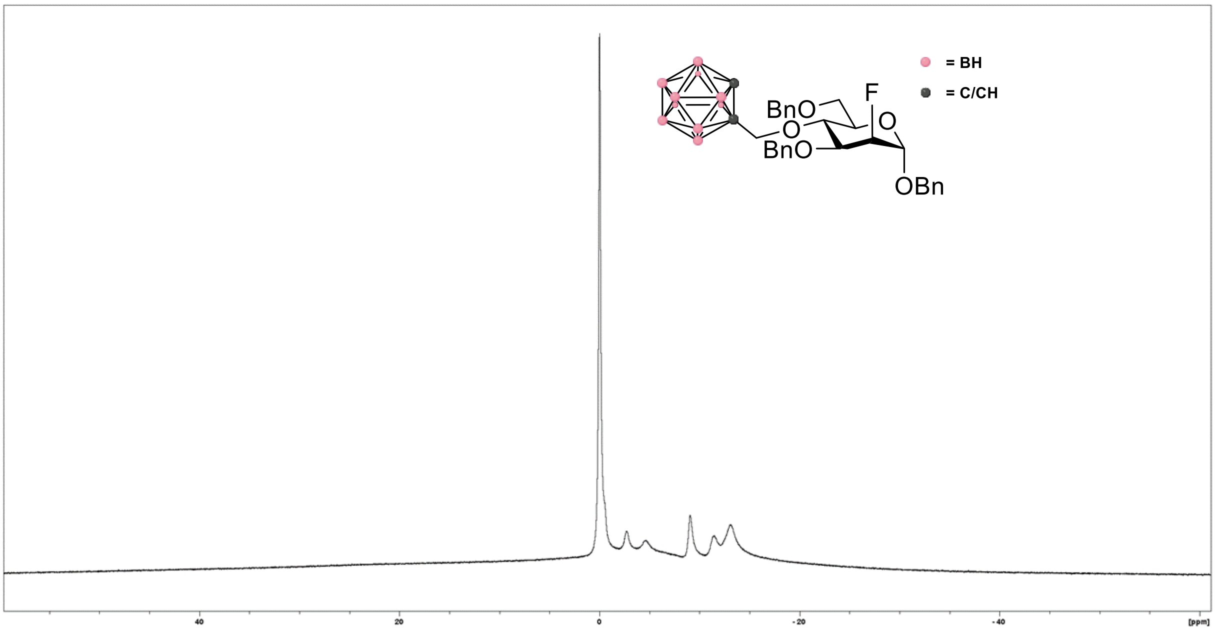

Figure S50. $^{11}B\{^{1}H\}$ NMR spectrum of **Benzyl 3,6-di-*O*-benzyl-2-deoxy-2-fluoro-4-*O*-carboranylmethyl-α-D-mannopyranoside** (160.36 MHz, 25 °C, $CDCl_3$).

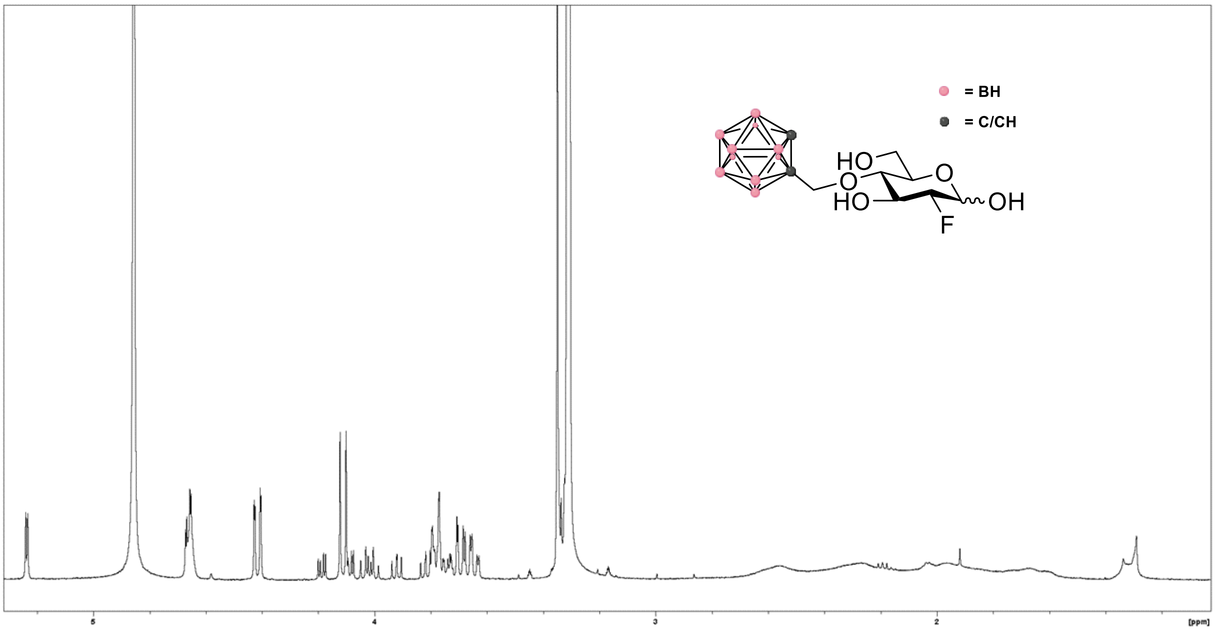

Figure S51. ^{1}H NMR spectrum of **2-deoxy-2-fluoro-4-*O*-carboranylmethyl-D-glucopyranose (2)** (499.83 MHz, 25 °C, CD_3OD).

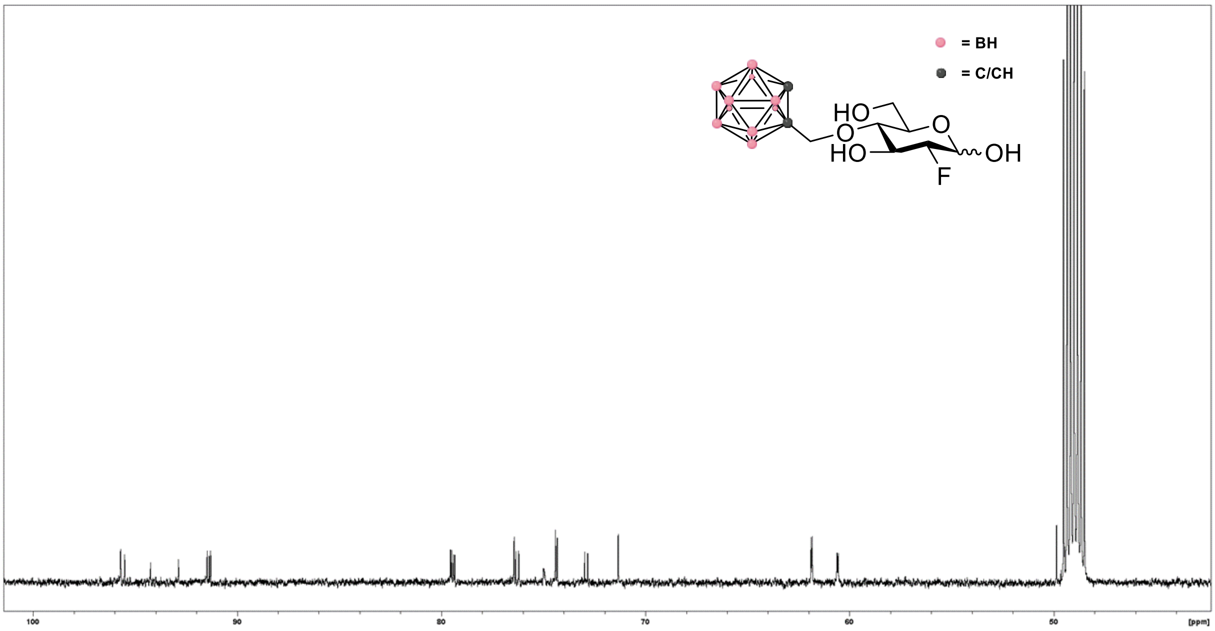

Figure S52. $^{13}C\{^{1}H\}$ NMR spectrum of **2-deoxy-2-fluoro-4-*O*-carboranylmethyl-D-glucopyranose (2)** (125.69 MHz, 25 °C, CD_3OD).

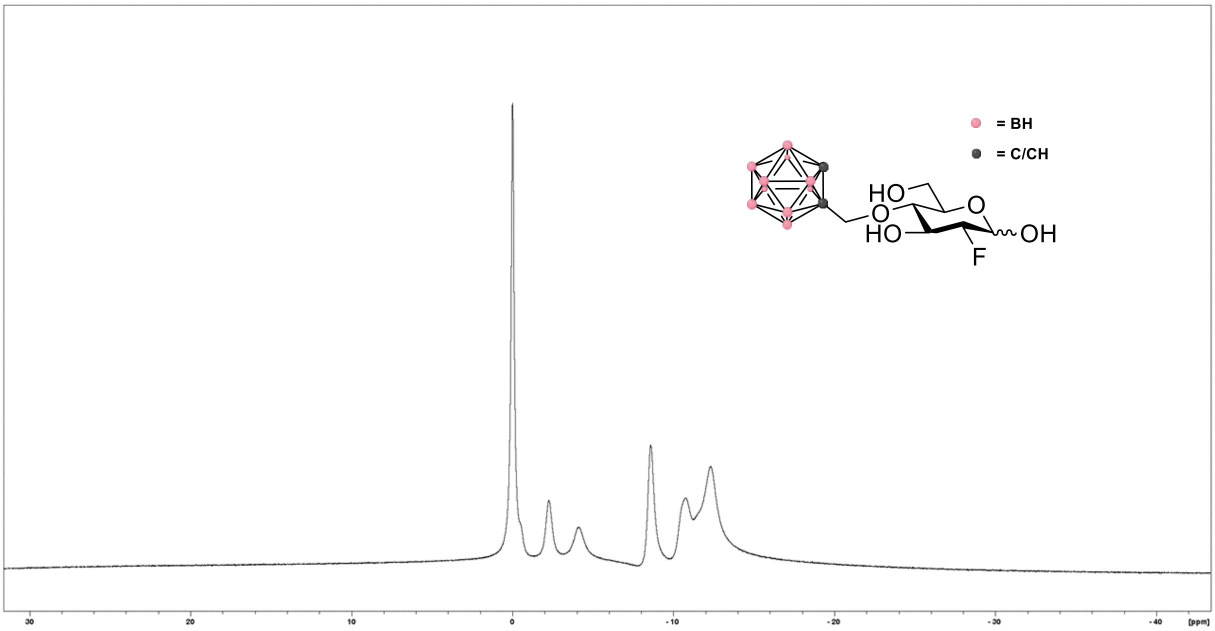

Figure S53. $^{11}B\{^{1}H\}$ NMR spectrum of **2-deoxy-2-fluoro-4-*O*-carboranylmethyl-D-glucopyranose (2)** (160.36 MHz, 25 °C, CD_3OD).

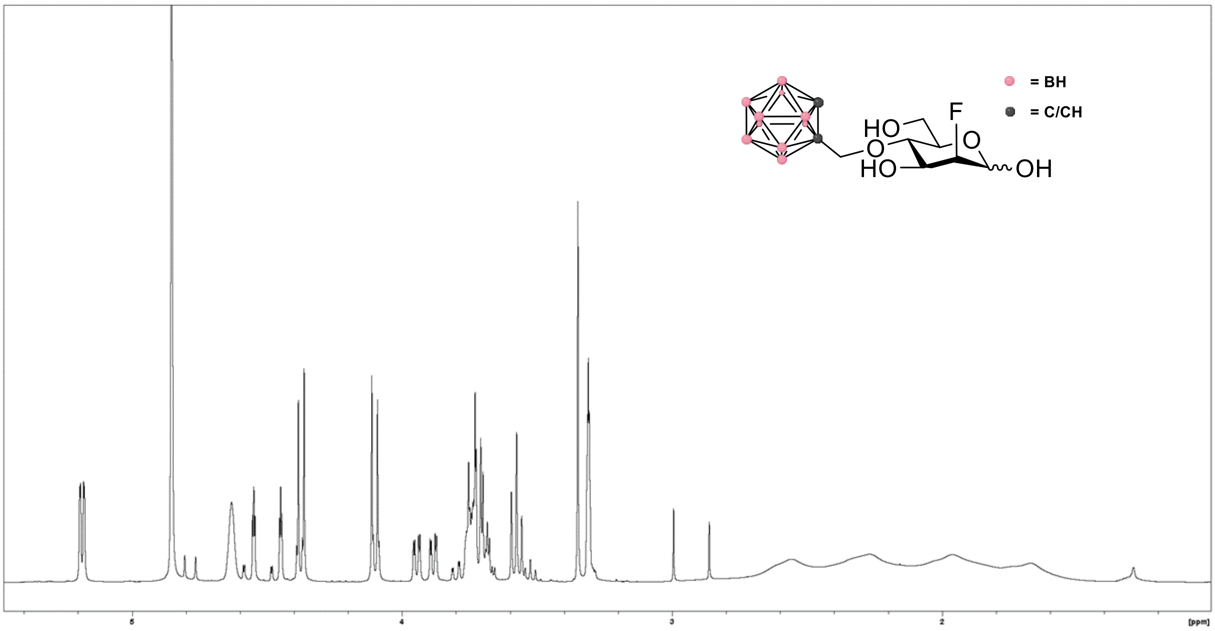

Figure S54. ^{1}H NMR spectrum of **2-deoxy-2-fluoro-4-*O*-carboranylmethyl-D-mannopyranose (4)** (499.83 MHz, 25 °C, CD_3OD).

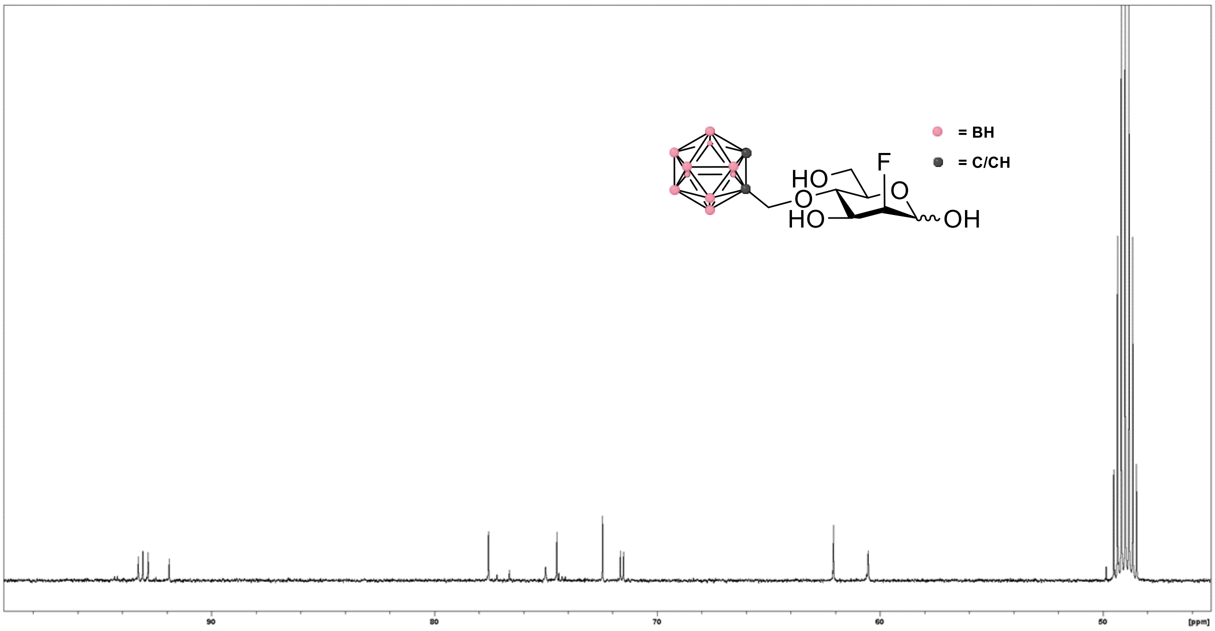

Figure S55. $^{13}C\{^1H\}$ NMR spectrum of **2-deoxy-2-fluoro-4-*O*-carboranylmethyl-D-mannopyranose (4)** (125.69 MHz, 25 °C, CD_3OD).

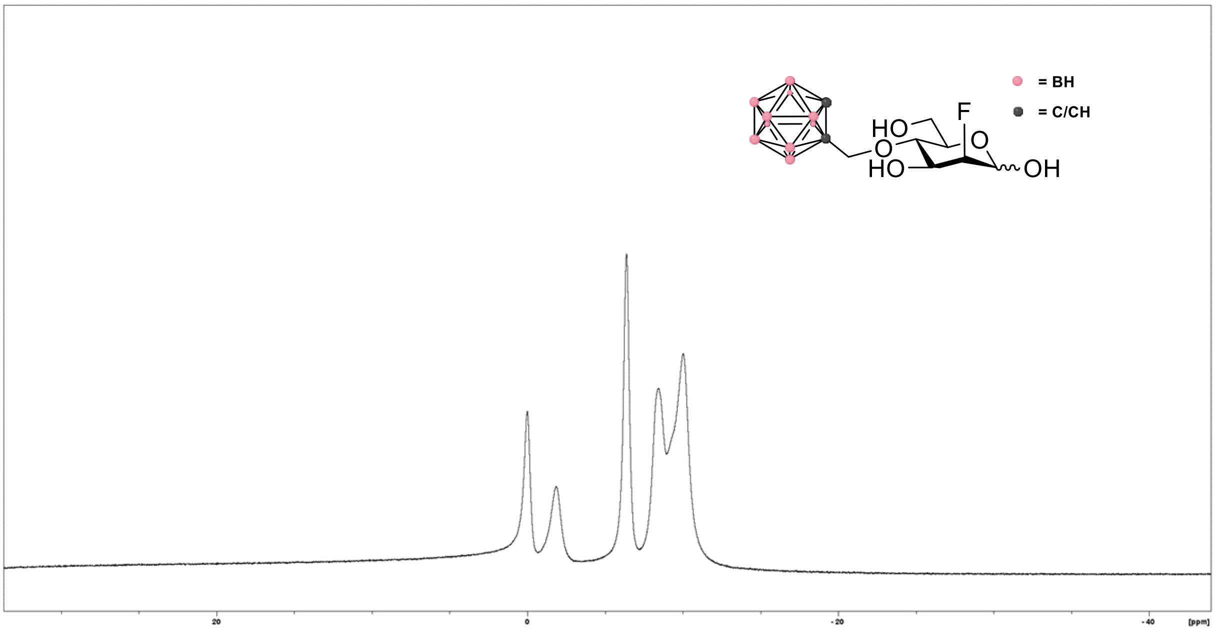

Figure S56. $^{11}B\{^1H\}$ NMR spectrum of **2-deoxy-2-fluoro-4-*O*-carboranylmethyl-D-mannopyranose (4)** (160.36 MHz, 25 °C, CD_3OD).
